# Chemistry and Biological Activity of 11*H*-Indeno[1,2-*b*]quinoxalin-11-ones and Tryptanthrins, Their Oximes, and Related Analogues

**DOI:** 10.3390/molecules31173032

**Published:** 2026-08-28

**Authors:** Igor A. Schepetkin, Mark B. Plotnikov, Anastasia R. Kovrizhina, Andrei I. Khlebnikov

**Affiliations:** 1 Department of Plant Science and Plant Pathology, Montana State University, Bozeman, MT 59717, USA; 2 Department of Pharmacology, Goldberg Research Institute of Pharmacology and Regenerative Medicine, Tomsk National Research Medical Center, Russian Academy of Sciences, Tomsk 634028, Russia; mbp2001@mail.ru; 3 Kizhner Research Center, Tomsk Polytechnic University, Tomsk 634050, Russia; anaskowry@gmail.com

**Keywords:** anticancer, tetracyclic ketoxime, cardioprotection, kinase inhibitor, neuroprotection, 11*H*-indeno[1,2-*b*]quinoxalin-11-one, indolo[2,1-*b*]quinazolin-6,12-dione, kinase inhibitor, neuroprotection

## Abstract

Nitrogen-containing fused tetracyclic systems, exemplified by the synthetic 11*H*-indeno[1,2-*b*]quinoxalin-11-one core and the natural alkaloid tryptanthrin (indolo[2,1-*b*]quinazolin-6,12-dione), constitute structural scaffolds whose rigid, planar architecture enables high-affinity interaction with nucleic acids and kinase active sites. Converting the exocyclic carbonyls at C-11 and C-6, respectively, into oximes has become a productive strategy in medicinal chemistry. This transformation modulates frontier orbital energies, installs N,O- and N,N-chelating pharmacophores, and enables nitric oxide (NO) release. Here, we summarize current knowledge of the synthesis, stereochemical characterization, and diverse biological activities of these tetracyclic ketoximes and related derivatives. Microwave, sonochemical, visible-light photocatalytic, and multicomponent methods now afford efficient, economical routes to the parent ketones and their oximes. X-ray crystallography, spectroscopy, and density functional theory have firmly established the thermodynamic preference for the *E*-oxime configuration and clarified how this geometry, along with potential target-induced isomerization, shapes binding. The oximes bind c-Jun N-terminal kinases (JNK1–3) with high affinity, a property that accounts for their neuroprotective effects in models of cerebral ischemia and Alzheimer-like pathology, their dual JNK inhibition and NO-mediated cardioprotection in hypertension and myocardial infarction, and their anti-inflammatory activity via suppression of NF-κB/AP-1 signaling. Broader studies also document anticancer, antimicrobial, antiviral, and antidiabetic activities arising from DNA intercalation, topoisomerase inhibition, metal-ion coordination, and kinase blockade. Compelling preclinical profiles notwithstanding, low oral bioavailability and rapid hepatic clearance remain major pharmacokinetic obstacles. Ongoing work on new formulations, prodrug strategies, and structure–activity optimization seeks to slow systemic elimination. Precise stereochemical definition combined with pleiotropic pharmacology positions tetracyclic ketoximes as attractive candidates for next-generation agents against complex multifactorial diseases.

## 1. Introduction

Nitrogen-containing tetracyclic heterocycles have long occupied a privileged position in medicinal chemistry, serving as foundational scaffolds for the development of targeted therapeutics across a diverse array of disease states [1]. Among these, the indoloquinazoline and indenoquinoxaline frameworks have garnered sustained attention. Their classification as “privileged structures” arises from their rigid, fused, and flat π-conjugated framework. This planar architecture enables strong interactions with biological targets, particularly through intercalation into DNA and binding within kinase active sites [2,3]. The two most extensively investigated representatives of this chemical class are the natural alkaloid tryptanthrin (indolo[2,1-*b*]quinazolin-6,12-dione) and the synthetic core 11*H*-indeno[1,2-*b*]quinoxalin-11-one. The latter was synthesized by Pearson et al. [4] in the early 1960s. However, its pharmacological importance was not realized for decades. Historically, tryptanthrin was first isolated as a sublimation product of natural indigo and was later identified in the culture filtrates of the yeast *Candida lipolytica*, as well as in various medicinal plants, including *Isatis tinctoria*, *Polygonum tinctorium*, and *Strobilanthes cusia* [5,6]. Early ethnopharmacological applications of these indigo-bearing plants in traditional Chinese and European medicine highlighted their profound anti-inflammatory, antipyretic, antiviral, and dermatological benefits. However, the precise bioactive constituents responsible for these effects remained elusive for decades [7]. The structural elucidation of tryptanthrin by X-ray crystallography in the early 1970s confirmed its planar tetracyclic architecture, establishing a structural paradigm that would later prove critical for understanding its target engagement and guiding subsequent pharmacokinetic optimization [8,9].

A major turning point in the development of these scaffolds was the recognition that their exocyclic carbonyl groups (specifically, the C-6 carbonyl in tryptanthrin and the C-11 carbonyl in indenoquinoxalinone) are reactive electrophilic centers. This reactivity enables their straightforward chemical modification to generate new derivatives. While the parent ketones exhibit baseline biological activity, their therapeutic potential in a clinical setting is frequently constrained by limited aqueous solubility, rapid metabolic clearance, and modest target affinity [10,11]. The strategic conversion of these carbonyl moieties into oximes (C=N–OH) emerged as a transformative medicinal chemistry strategy [12,13]. Oximation fundamentally alters the electronic landscape, hydrogen-bonding capacity, and pharmacophoric profile of the tetracyclic core. Specifically, oxime functionalization introduces a potent chelating motif that enhances transition metal coordination, modulates electron density distribution, and frequently improves membrane permeability without compromising the planar integrity required for intercalative or kinase-binding interactions [14,15]. Moreover, the oxime group confers unique redox properties. Recent electron paramagnetic resonance (EPR) studies have shown that these oximes are metabolized via oxidoreductive pathways to release nitric oxide (NO), while also modulating reactive oxygen species (ROS). This dual activity expands their mechanism of action to include vasodilation and the regulation of oxidative stress [16,17,18].

**Figure 1:**
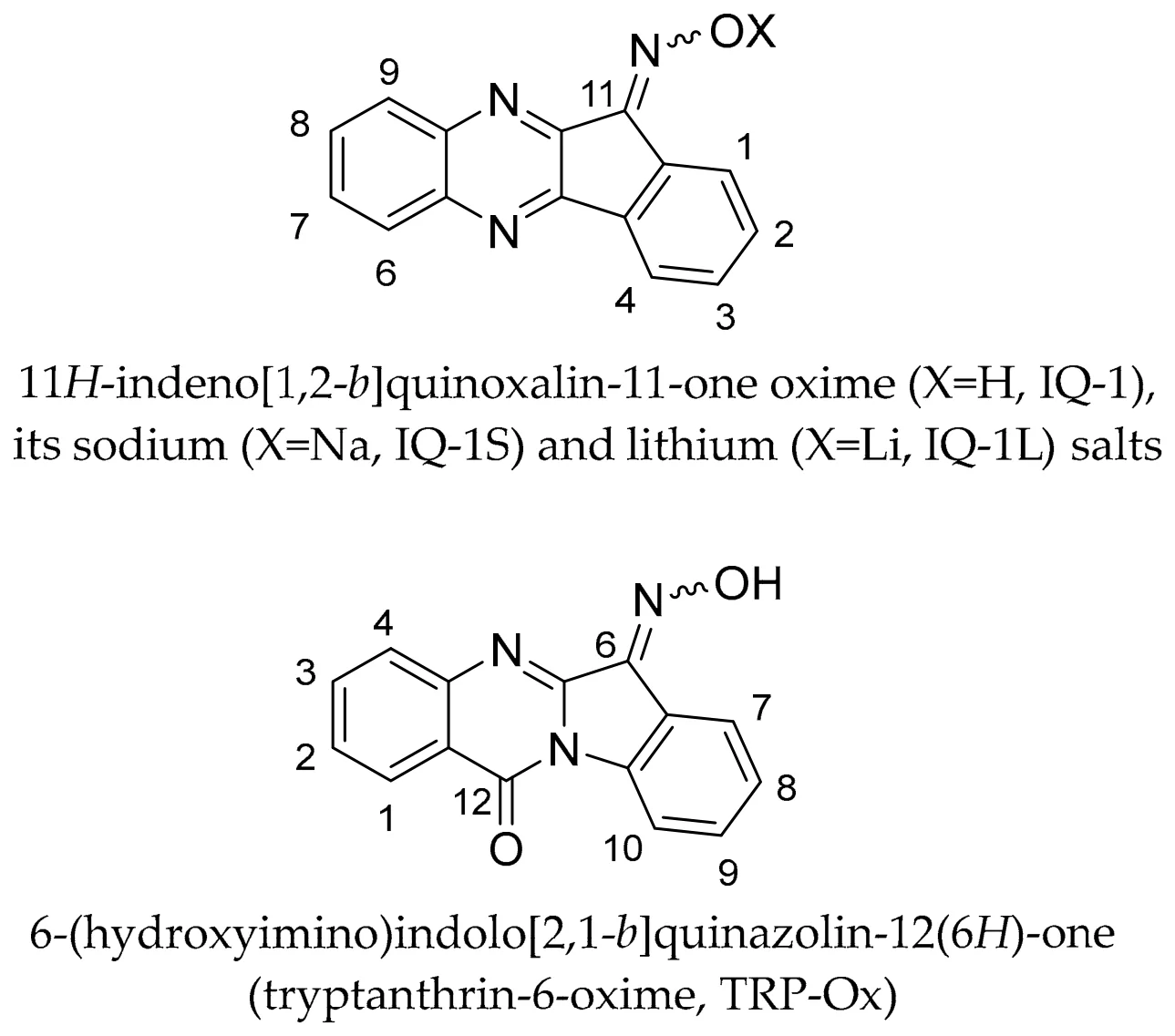
Chemical structures of some JNK1-3 inhibitors with indenoquinoxaline (**top**) and tryptanthrin (**bottom**) skeletons.

11*H*-Indeno[1,2-*b*]quinoxalin-11-one oxime (IQ-1) and its structural analogs have attracted great attention since the discovery that they may function as high-affinity, isoform-selective inhibitors of the c-Jun N-terminal kinase (JNK) family [19]. These oximes bind to JNK1 and JNK3 with nanomolar dissociation constants (Figure 1), establishing a direct link between oxime-mediated structural preorganization and the potent suppression of stress-activated signaling cascades [20]. This finding opened new prospects for the treatment of neurodegenerative, inflammatory, and ischemic pathologies. Subsequent investigations revealed that tryptanthrin and its derivatives also amplify anticancer, antimicrobial, antiparasitic, and antiviral activities. For instance, tryptanthrins have demonstrated potent antiviral activity against human coronaviruses (e.g., HCoV-NL63) by blocking viral RNA genome synthesis [21] and have shown exceptional antiparasitic efficacy against *Toxoplasma gondii* and *Plasmodium falciparum* in the low nanomolar range [22,23]. Furthermore, recent structural tuning has yielded multi-target-directed ligands (MTDLs) capable of simultaneously inhibiting acetylcholinesterase (AChE) and butyrylcholinesterase (BuChE) for Alzheimer’s disease (AD) therapy [24,25], as well as agents that disrupt inflammasome assembly and topoisomerase function [26,27,28]. The combination of these diverse biological effects underscores the unique versatility of tryptanthrin and indenoquinoxalinone tetracyclic scaffolds in addressing complex, multifactorial diseases.

Additionally, the emergence of pathogen- and tumor-mediated resistance mechanisms necessitates a deeper understanding of structure–activity relationships (SAR). For example, while mycobacteria can develop resistance via efflux pump upregulation [29], researchers are concurrently discovering that tryptanthrin can be used to target glutathione S-transferase P1 (GSTP1), inducing cellular senescence and sensitizing resistant tumors to senolytic combination therapies [30].

This review provides a comprehensive and critical analysis of the current state of research on 11*H*-indeno[1,2-*b*]quinoxalin-11-one oxime and tryptanthrin-6-oxime, their ketone precursors, and related compounds. We begin by examining the synthetic methodologies that enable the efficient construction of the tetracyclic cores and the carbonyl-directed oximation protocols that preserve scaffold integrity. The stereochemical peculiarities of the oxime bond and its implications for target recognition are then addressed. Scaffold diversification strategies, including spiro-heterocycle formation and transition metal coordination are also explored. The essential sections of the review systematically evaluate the broad biological activity of these oximes and their derivatives, with dedicated sections on neuroprotective, cardioprotective, anti-inflammatory, antitumor, and antimicrobial effects, alongside a detailed analysis of their high kinase-binding affinity and alternative mechanistic pathways. Finally, we discuss the pharmacokinetic limitations, toxicological considerations, and strategic perspectives required to advance tetracyclic ketoximes from promising preclinical leads to clinically validated therapeutics. The term “tetracyclic ketoximes” throughout this review will be mainly attributed to 11*H*-indeno[1,2-*b*]quinoxalin-11-one oxime and tryptanthrin-6-oxime (Figure 1). By integrating recent structural, computational, and pharmacological insights, this review aims to provide a framework for the rational design and future development of this privileged chemical class. One of the review authors’ goals was also to draw the attention of scientists, particularly those seeking new, effective small-molecule compounds for the treatment of socially significant diseases, to this group of promising molecules.

## 2. Chemical Characterization and Synthesis of 11*H*-Indeno[1,2-*b*]quinoxalin-11-one, Tryptanthrin, and Their Derivatives

### 2.1. Tetracyclic Core Architecture and Carbonyl Reactivity

The pharmacological versatility and synthetic utility of tetracyclic ketoximes arise from the structural rigidity, extended π-conjugation, and unique stereoelectronic properties of their parent heterocyclic scaffolds. Both 11*H*-indeno[1,2-*b*]quinoxalin-11-one and indolo[2,1-*b*]quinazoline-6,12-dione comprise a fully fused, highly conjugated tetracyclic architecture that integrates an electron-deficient quinoxaline or quinazoline moiety with a relatively electron-rich aromatic ring [11,14]. X-ray crystallographic analyses have consistently confirmed the near-perfect planarity of these cores. This geometric flatness facilitates efficient π-π stacking interactions, often with centroid–centroid distances of approximately 3.7 Å. Hence, these cores are capable of intercalative binding with nucleic acids and optimal insertion into the narrow, planar ATP-binding pockets of various kinases [8,31]. Furthermore, the rigidity of the fused tetracyclic system minimizes the loss of conformational entropy upon target engagement, thereby thermodynamically enhancing the binding affinity and target selectivity, as evidenced in extensive SAR studies targeting kinases and topoisomerases [5,32,33].

Central to the chemical reactivity and functional diversification of these scaffolds is the exocyclic carbonyl group, positioned at C-11 in indenoquinoxalines and C-6 in tryptanthrins. The reactivity of this electrophilic carbonyl carbon is modulated by the adjacent electron-withdrawing nitrogen atoms within the fused pyrazine or pyrimidine rings, which pull electron density away from the carbonyl, thereby significantly enhancing its electrophilicity [34,35]. Computational studies employing density functional theory (DFT), alongside molecular electron density theory, have demonstrated that this carbonyl group dictates the electron distribution and modulates the HOMO-LUMO energy gaps [36,37,38]. These electronic parameters directly correlate with the molecule’s global reactivity descriptors, redox behavior, and the stabilization of receptor–ligand complexes [39,40]. Cyclic voltammetry and quantum chemical analyses further indicate that the ability of the carbonyl oxygen to accept electron transfer is a crucial determinant for the biological activity of the parent scaffolds [34]. Consequently, this highly polarized electronic configuration not only facilitates nucleophilic attack under mild conditions but also allows for the precise tuning of molecular softness and dipole moments without disrupting the aromatic integrity of the tetracyclic core [41,42].

**Figure 2:**
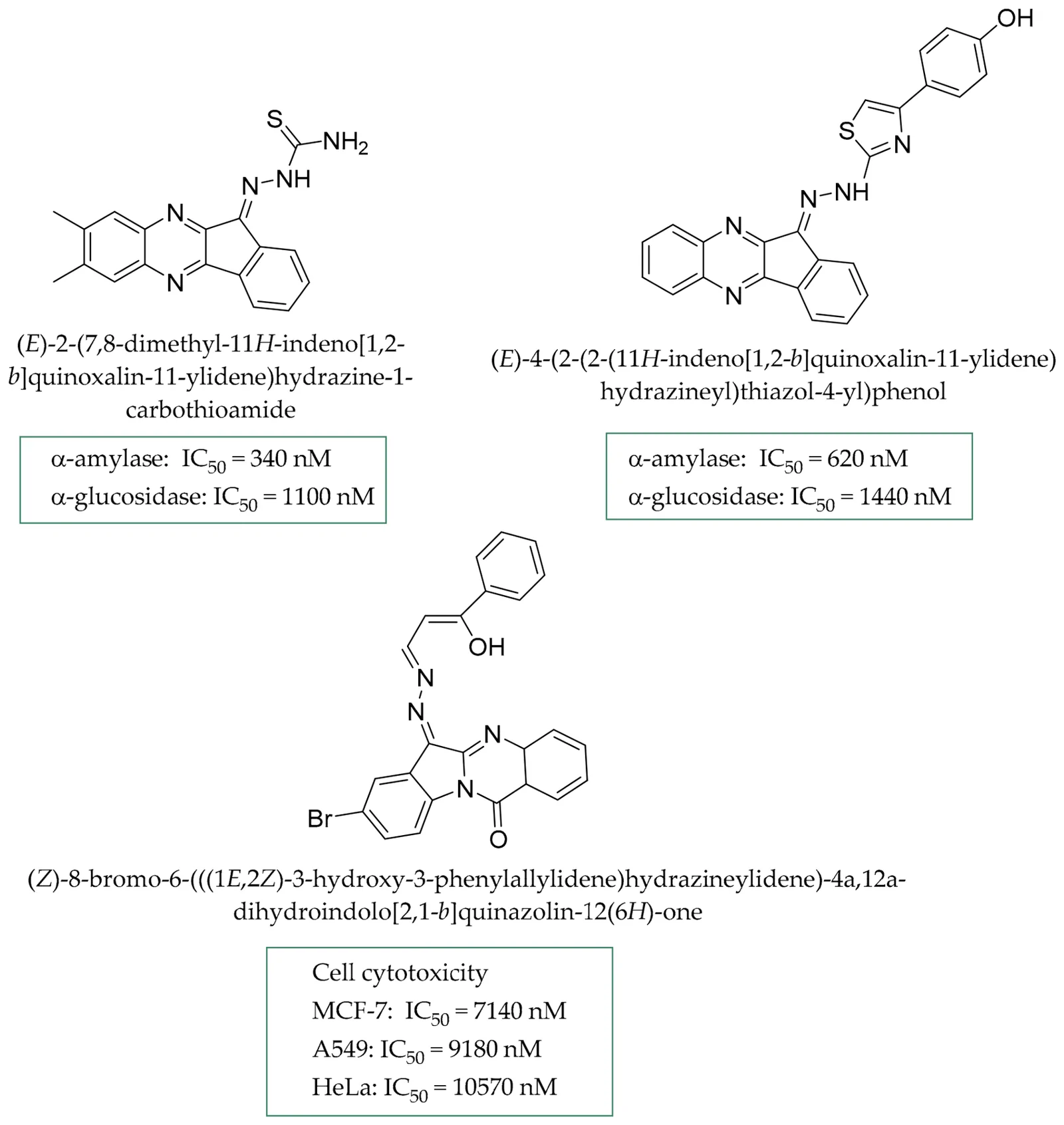
Some biologically active thiosemicarbazone, azine, and hydrazinylthiazole hybrids with indenoquinoxalinone or tryptanthrin.

Converting this exocyclic carbonyl group into an oxime (>C=N–OH) is a key synthetic strategy for developing biologically active derivatives. Oximation proceeds efficiently under mild, often catalyst-free conditions, preserving the integrity of the sensitive fused heterocyclic framework while introducing a highly versatile pharmacophoric chelating motif [19,43]. The resulting oxime functionality exhibits a stereoelectronic landscape that is distinctly different from the parent ketone. It introduces a potent hydrogen-bond donor (the hydroxyl proton) and an additional hydrogen-bond acceptor (the imine nitrogen), significantly altering p*K*_a_ of the compound, modulating its lipophilicity, and generally enhancing its aqueous solubility profile, factors that are critical for optimizing absorption, distribution, metabolism, and excretion (ADME) parameters [37,44]. Beyond simple oximes, the highly reactive C-11/C-6 carbonyl readily undergoes condensation with hydrazines, thiosemicarbazides, and primary amines to yield an array of hydrazones, thiosemicarbazones, and other derivatives. For instance, the conversion of the C-6 carbonyl into hydrazinyl thiazole hybrids or C-6 hydrazones has been shown to effectively expand the chemical space, generating multi-target agents (Figure 2) while maintaining the essential planar architecture required for target recognition [35,45,46,47].

Despite the steric bulk inherent to large tetracyclic systems, the exocyclic carbonyl remains highly accessible to nucleophiles due to its peripheral location on the ring system and the general absence of significant *ortho*-substituent steric hindrance. The spatial accessibility of this position allows for highly selective functionalization, which is particularly useful in cascade and multicomponent reactions for the rapid preparation of diverse compound libraries [48,49]. The reactivity of the carbonyl group is also exploited in complex metal-catalyzed and organocatalytic transformations. It readily participates in enantioselective additions and ketimine formations and acts as a linchpin for the construction of complex spiro-heterocycles via 1,3-dipolar cycloadditions or ring-closing enyne metathesis of propargyl ethers [50,51]. Importantly, the preservation of the rigid tetracyclic core during these modifications ensures that the resulting derivatives retain the essential structural prerequisites for high-affinity biomolecular interactions. At the same time, the newly introduced functional groups provide the necessary chemical diversity for fine-tuning biological activity, overcoming resistance mechanisms, and enhancing target selectivity [11,14].

Thus, the rigid core structure and the reactive carbonyl group together provide a versatile platform for the rational design of tetracyclic ketoximes. The planarity of the core, the reactivity of the carbonyl group, and the available derivatization routes collectively influence both the ease of synthesis and the physicochemical and pharmacological properties of these compounds. This structural framework thus connects synthetic chemistry directly to biological evaluation and enables the stereochemical control, scaffold diversification, and transition-metal coordination strategies discussed in the following sections.

### 2.2. Sustainable and Multicomponent Synthetic Strategies

These scaffolds have been prepared by a considerable variety of synthetic methods. The strategies surveyed in this section are, therefore, organized into two broad groups. The first encompasses constructions of the parent tetracyclic frameworks, most notably the tryptanthrin and indenoquinoxaline cores, by microwave-assisted, photocatalytic, sonochemical, and electrochemical procedures. The second comprises oximation of the carbonyl function and subsequent elaboration, for instance by multicomponent spiro-cyclizations or metal complexation. To aid comparison, the key practical features of the reactions shown in the accompanying schemes, atom economy, reaction time, and substrate compatibility, are summarized briefly below.

Synthesis of tetracyclic ketoximes and their parent ketones now increasingly follows green-chemistry principles. The drivers are clear: lower environmental burden, simpler experimental workflows, and faster SAR campaigns. Older routes to the indenoquinoxaline and tryptanthrin cores typically demanded prolonged heating, stoichiometric oxidants, and hazardous organic solvents, constraints that restricted both scalability and sustainability. Newer methods have displaced those approaches with microwave-assisted, sonochemical, visible-light photocatalytic, electrochemical, and multicomponent protocols. The newer routes emphasize atom economy, step economy, and rapid library assembly while leaving sensitive functional groups intact.

**Scheme 1:**
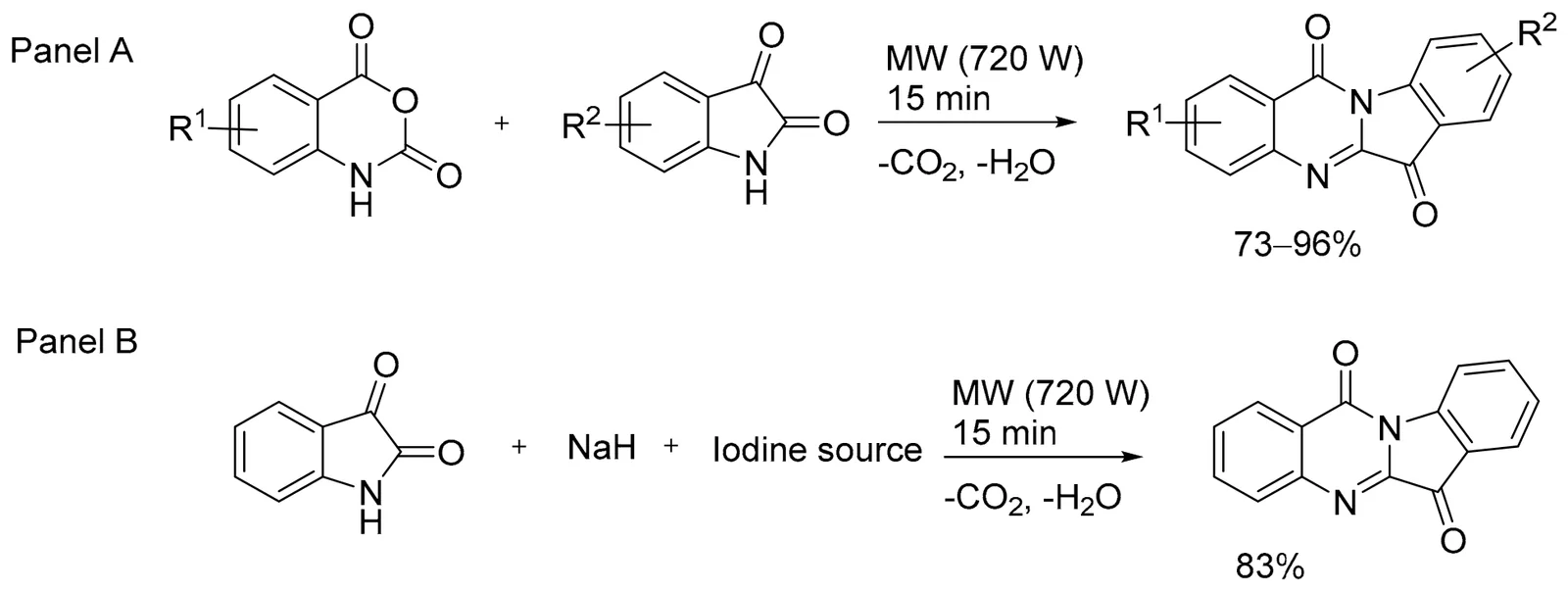
Synthetic routes from isatins to tryptanthrins by microwave (MW) irradiation method.

Microwave irradiation under solvent-free conditions has proved especially effective for building the rigid tetracyclic framework. Azizian et al. [52] introduced a one-pot, three-component microwave procedure that delivers dicyanomethylene derivatives of both indenoquinoxaline and tryptanthrin without a solvent, cutting reaction times from hours to minutes and still furnishing high yields. Extending the same thermal-acceleration concept, a catalyst- and solvent-free microwave protocol was later devised for tryptanthrin derivatives through decarboxylative–dehydrative addition of isatoic anhydrides to isatins [53] (Scheme 1A). The method sidesteps toxic solvents and produces compounds that display selective cytotoxicity toward prostate cancer cell lines while sparing normal epithelial cells. In a related development, microwave irradiation paired with the I_2_/NaH/DMF oxidant system converts indigo or isatin into tryptanthrin rapidly under mild conditions [54], underscoring how thermal acceleration remains compatible with varied oxidative settings (Scheme 1B).

**Scheme 2:**
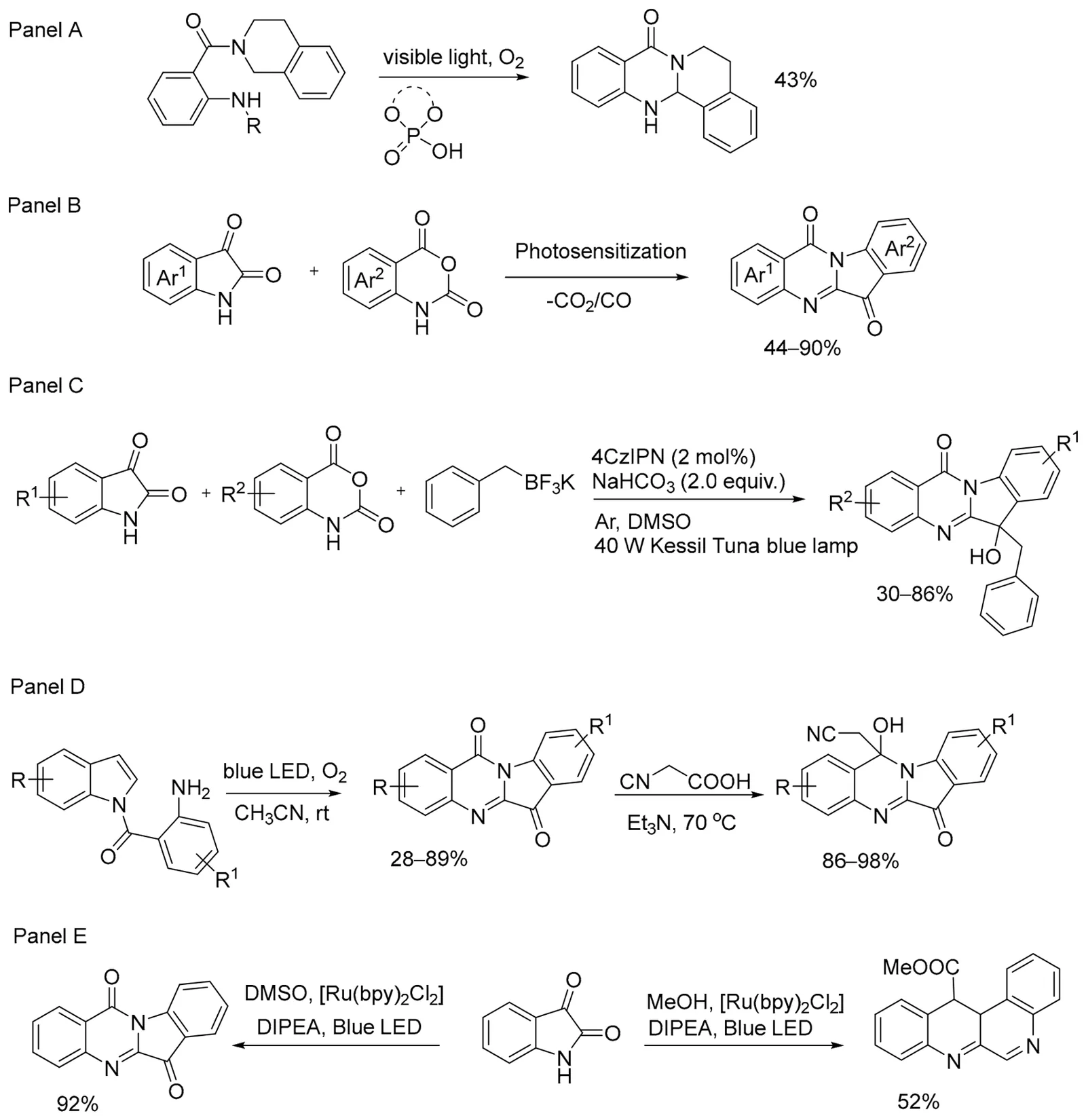
Synthetic routes to tryptanthrin and its analogues using photocatalysis. Abbreviations: 4CzIPN, 1,2,3,5-*tetrakis*(carbazol-9-yl)-4,6-dicyanobenzene; DMSO, dimethyl sulfoxide; LED, light-emitting diode; DIPEA, *N*,*N*-diisopropylethylamine.

Visible-light photocatalysis has further revolutionized the assembly of these heterocycles by eliminating the need for toxic transition metals and harsh thermal conditions. Jing et al. [55] reported a metal- and photocatalyst-free visible-light-driven intramolecular C–N cross-coupling mediated by a long-lived photoisomer complex (Scheme 2A). This room-temperature method enabled the gram-scale synthesis of tryptanthrin and its analogues and tolerated a wide range of substrates. Complementary work [56] utilized Rose Bengal as an inexpensive organic dye to mediate energy transfer processes for the self- or cross-condensation of isatin and isatoic anhydride (Scheme 2B). Expanding on this, a visible-light-induced radical–radical cross-coupling method was developed [57] for the one-pot synthesis of tryptanthrin derivatives from isatins and potassium benzyl trifluoroborates, allowing simultaneous C–C and C–N bond formation without external oxidants (Scheme 2C).

More recently, a photocatalyst-free, visible-light-induced aerobic intramolecular cyclization was achieved via single-electron transfer (SET) between the substrate and molecular oxygen [58] (Scheme 2D). Furthermore, photoredox catalysis was shown to promote the chemodivergent conversion of isatins [59], providing a scalable and straightforward route to tryptanthrin architectures (Scheme 2E). These photochemical routes highlight a definitive shift toward ambient-temperature, energy-efficient methodologies.

Microwave protocols in Scheme 1 stand out for their high rates. Reactions typically finish in minutes, and they avoid volatile organic solvents. These methods also accommodate a wide array of isatin and isatoic anhydride derivatives, delivering the products in high yield under mild, solvent-free conditions [52,53,54]. Photocatalytic routes collected in Scheme 2 complement this picture by operating at room temperature and dispensing with toxic transition metals. Thus, Jing et al. [55] developed a procedure that accepts more than fifty different substrates and scales readily to the gram level. The visible-light-driven radical–radical coupling of Huang et al. [57] produced 31 products in 22–86% yield without added oxidants, illustrating its generous functional-group tolerance.

**Scheme 3:**
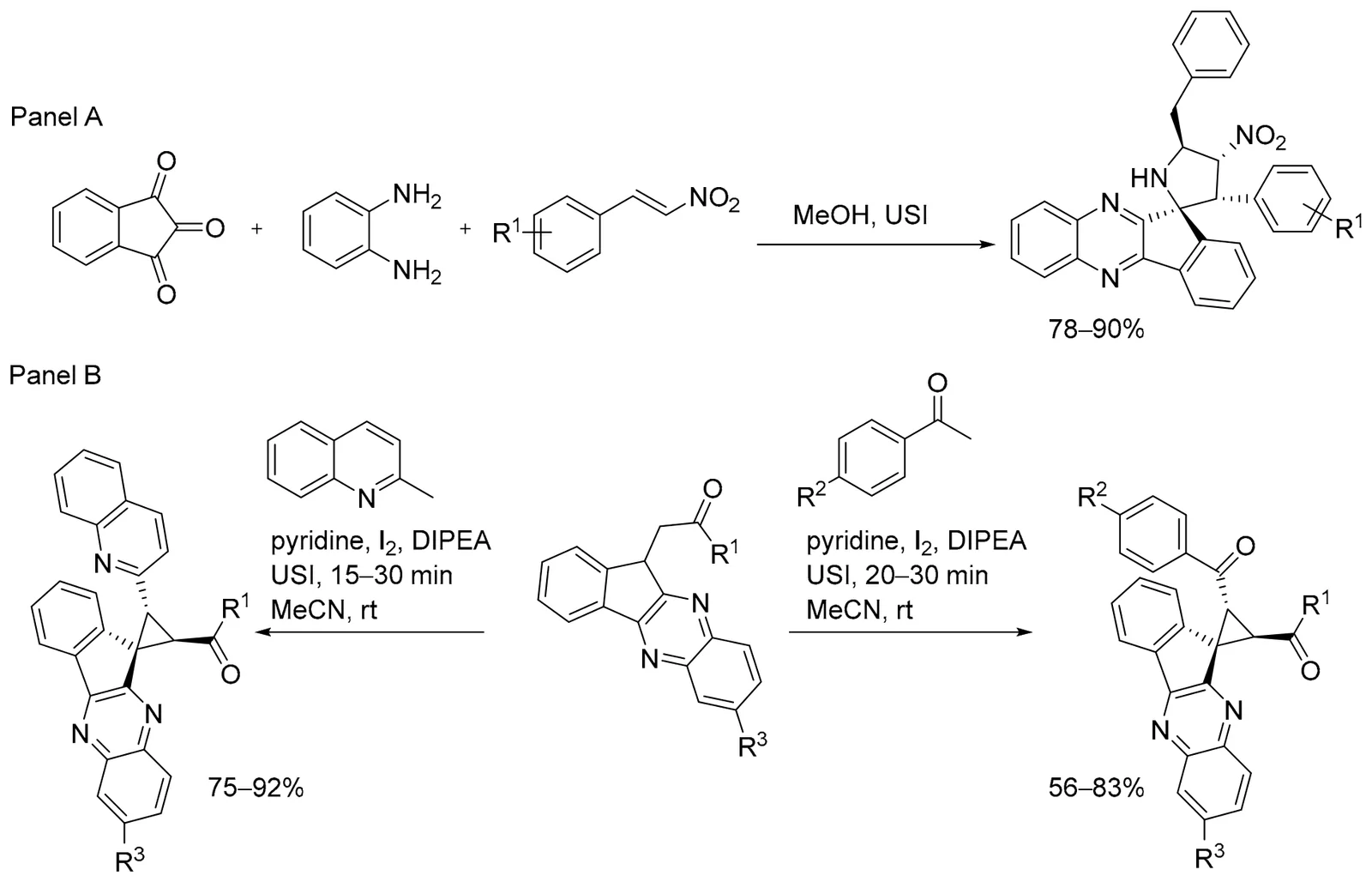
The synthesis of indenoquinoxaline derivatives via ultrasound irradiation (USI).

Sonochemistry and biocatalysis have also been used to accelerate reactions and improve the sustainability of the processes. Thus, Mane et al. [60] coupled high-intensity ultrasound with baker’s yeast (*Saccharomyces cerevisiae*) biocatalysis for tryptanthrin synthesis. Ultrasonic disruption of yeast cells facilitated rapid enzyme release, accelerating the biocatalyzed transformation while avoiding toxic metal catalysts and generating no detectable byproducts. In multicomponent reactions, ultrasound irradiation has been employed for organocatalytic domino processes [61,62], affording spiro-indenoquinoxaline derivatives in excellent yields under ambient conditions (Scheme 3A). The application of ultrasound has also been extended to the synthesis of spirocyclopropanes via azomethine ylide [3+2]-cycloadditions, yielding complex architectures with excellent stereoselectivity [63] (Scheme 3B). Quantitative green metrics, such as high atom economy, low E-factor, and minimal process mass intensity, confirm the environmental superiority of these sonochemical protocols.

**Scheme 4:**
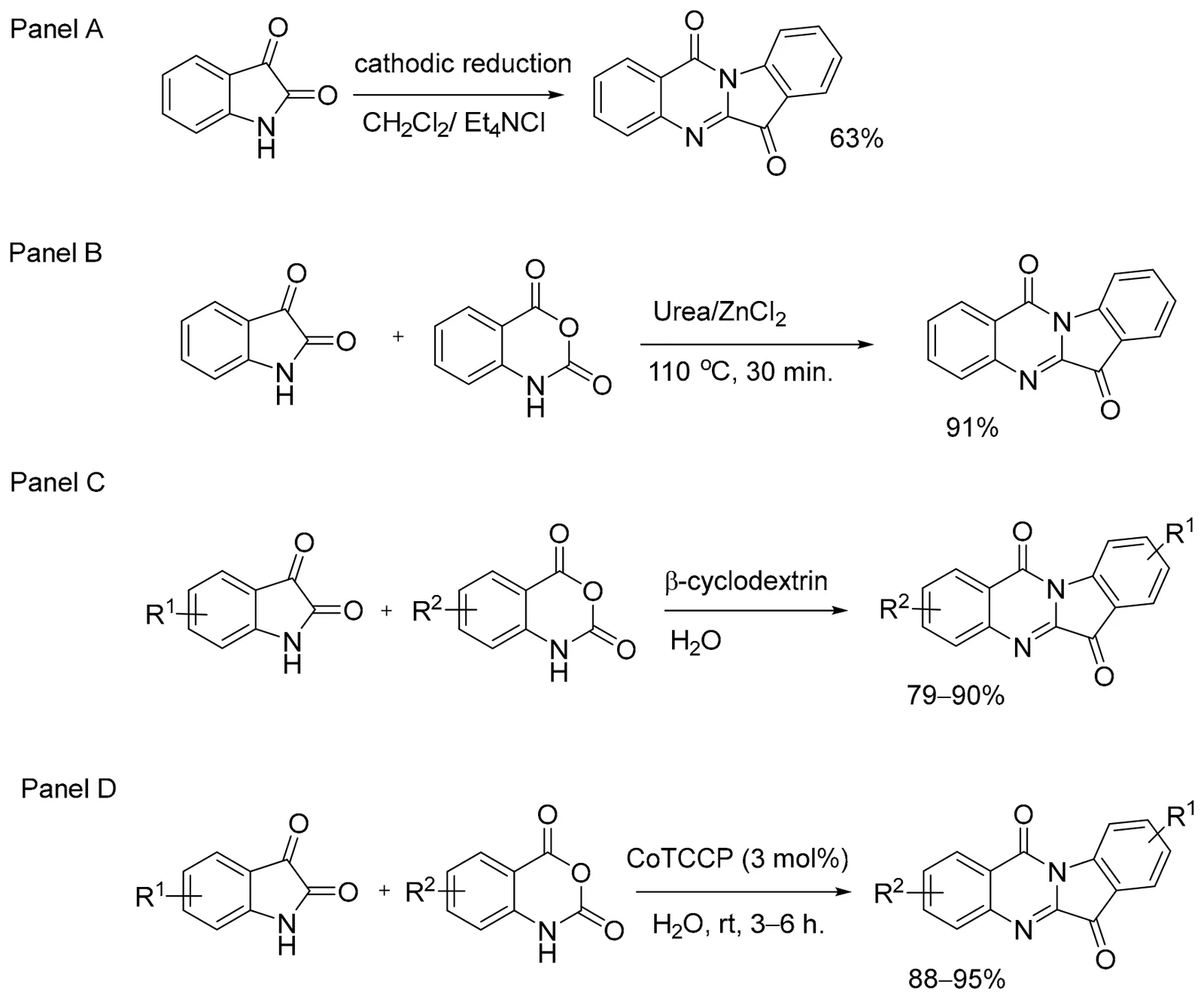
Synthetic routes from isatins and isatoic anhydrides to tryptanthrin and its derivatives using electrochemical, aqueous, and deep eutectic solvent (DES) approaches.

Electrochemical methods, aqueous media, and deep eutectic solvents (DES) represent additional sustainable approaches for organic synthesis. An electrosynthetic route to tryptanthrin was developed through the cathodic reduction of isatin [64] using atmospheric oxygen as the terminal oxidant in a low-energy process (Scheme 4A).

In another strategy, Peña-Solórzano et al. [65] employed a urea/zinc chloride eutectic mixture as a recyclable, low-melting catalytic medium for the multicomponent synthesis of dihydroquinazolinones and tryptanthrin, achieving excellent yields with catalyst reuse over multiple cycles (Scheme 4B). Aqueous media have also proven highly effective. It was demonstrated [66] that β-cyclodextrin efficiently catalyzes the condensation of isatoic anhydride and isatin in water at room temperature (Scheme 4C). Similarly, a water-soluble cobalt(III) porphyrin complex (CoTCPP) was used as a highly efficient and recyclable homogeneous catalyst for tryptanthrin synthesis in aqueous media [67] (Scheme 4D), while a catalyst-free, three-component synthesis of indenoquinoxalines in water was reported [68], highlighting the feasibility of using entirely green solvent systems.

The sonochemical multicomponent reactions presented in Scheme 3 are notable for their strong quantitative green-chemistry profiles. Borah et al. [61] reported high atom economy, reaction mass efficiency, and carbon efficiency, together with a low E-factor and process mass intensity. These ultrasound-assisted protocols combine short reaction times with waste-free conditions and broad functional-group tolerance. In many cases, chromatographic purification can be avoided through group-assisted purification chemistry [61,62]. The methods shown in Scheme 4, by contrast, place particular emphasis on recyclable catalysts and environmentally benign reaction media. In the deep eutectic solvent approach, the urea/zinc chloride mixture can be reused for at least three cycles while preserving excellent yields and short reaction times [65]. The aqueous protocols offer similar practical advantages, including room-temperature conditions, high reaction rates, and readily recoverable catalysts [67], thereby supporting high step economy.

**Scheme 5:**
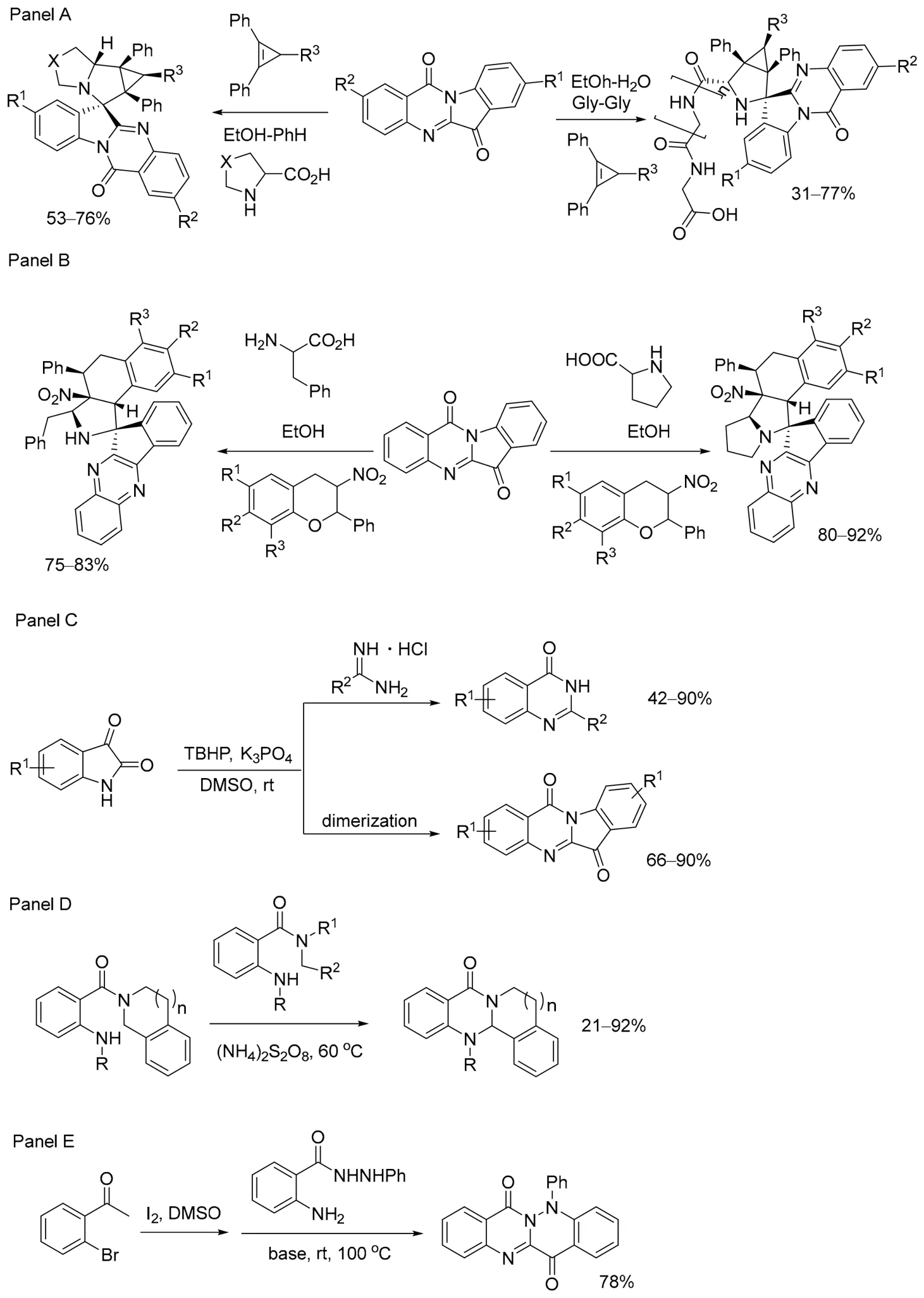
Multicomponent reactions (MCRs) and cascade/domino processes for synthesis of quinazoline derivatives.

Multicomponent reactions (MCRs) and cascade/domino processes have proven indispensable for rapid library generation, SAR optimization, and the construction of complex spiro-fused derivatives. By combining three or more starting materials in a single operational step, MCRs enhance the step economy and simplify downstream purification. Filatov et al. [69] utilized one-pot, three-component 1,3-dipolar cycloadditions to generate spiro-tryptanthrin analogues with high diastereoselectivity (Scheme 5A).

This approach has been widely adapted. For instance, sequential MCRs were employed to synthesize highly substituted spiro-indenoquinoxaline pyrrolidines (Scheme 5B), leveraging the in situ generation of azomethine ylides to achieve high regioselectivity and operational simplicity [70,71]. Oxidative cascade reactions also offer powerful synthetic pathways. Thus, access to functionalized quinazolinones and tryptanthrins was streamlined [72] via a *tert*-butyl hydroperoxide/K_3_PO_4_-promoted oxidative cyclization at room temperature (Scheme 5C), while a metal-free ammonium peroxodisulfate-mediated intramolecular oxidative cyclization was reported [73] for the synthesis of polycyclic quinazolinones (Scheme 5D). Other notable cascade strategies include the CuBr_2_-mediated single-electron transfer (SET) oxidative cyclization [74] and the iodine-mediated cascade reaction of 2′-bromoacetophenones [75] (Scheme 5E). As shown in Scheme 5, these multicomponent and post-modification strategies display strong atom economy. They enable rapid, one-pot construction of complex spiro-heterocycles with high levels of diastereoselectivity and regioselectivity. Their practical appeal lies in the combination of operational simplicity, high yields, broad substrate compatibility, and the capacity to generate multiple contiguous stereocenters in a single step. Together, these features make the methods especially well-suited for the efficient preparation of diverse libraries of tetracyclic derivatives [69,70].

One-pot multicomponent reactions have repeatedly proven valuable for assembling indenoquinoxaline- and tryptanthrin-fused spiroheterocycles, a point underscored in recent reviews [76]. Work in this area now centers on achieving high stereoselectivity while adhering to green-catalysis principles. A four-component route, for example, delivers spiro-indenoquinoxaline-pyrrolidine hybrids with strong endo-selectivity and clear antibacterial potency under mild, atom-economical conditions [77]. Parallel efforts have produced tryptanthrin-appended 4-spiropiperidines through an ammonium acetate-catalyzed green synthesis; the resulting compounds show activity against multidrug-resistant *S. aureus* isolates [78]. Magnetically recoverable nanocomposites such as Fe_3_O_4_@SiO_2_@CPTMS-PADETA have likewise allowed efficient, environmentally benign preparation of spiroindoloquinazolines, combining ready catalyst reuse with encouraging biological profiles [79].

Together, these sustainable and multicomponent methods provide simple, atom-economical routes that preserve the core structure while allowing rapid access to diverse derivatives, thereby supporting subsequent stereochemical control and biological evaluation.

### 2.3. Oxime Bond Stereochemistry: E/Z Isomerism and Thermodynamic Stability

The pharmacological activity, pharmacokinetic behavior, and molecular recognition properties of tetracyclic ketoximes depend strongly on the geometry of the exocyclic C=N–OH bond. Like other oximes, these compounds display classical *E*/*Z* isomerism. For many years, the stereochemical assignment of key derivatives, including IQ-1 and tryptanthrin-6-oxime, remained unresolved in the literature. Earlier structural models often favored the *Z*-configuration, largely because this arrangement was thought to be stabilized by an intramolecular hydrogen bond between the oxime hydroxyl proton and the neighboring nitrogen atom of the fused heterocyclic system. Recent experimental and computational studies, however, have resolved this ambiguity and established the *E*-isomer as the thermodynamically preferred and biologically relevant form in both the solid state and solution.

Matveevskaya et al. [44] provided a detailed stereochemical analysis of IQ-1 and tryptanthrin-6-oxime using single-crystal X-ray diffraction, 1D/2D NMR spectroscopy, and DFT calculations. The crystallographic results clearly assigned the oxime moiety to the *E*-configuration, with the hydroxyl group directed away from the tetracyclic core. This orientation prevents formation of the proposed intramolecular hydrogen bond and instead promotes extensive intermolecular hydrogen-bonding networks in the crystal lattice. By lowering lattice energy, these intermolecular contacts stabilize the *E*-isomer and contradict the earlier *Z*-configuration model. Solution NMR data supported the crystallographic findings, showing that the *E*-configuration is maintained in common organic solvents and does not undergo appreciable *E*/*Z* isomerization under ambient conditions.

Computational studies provide further support for the thermodynamic preference of the *E*-isomer. The destabilization of the *Z*-isomer arises primarily from steric repulsion between the oxime hydroxyl group and the rigid, planar tetracyclic core. Kovrizhina et al. [37] independently confirmed these trends in functionalized tryptanthrin-6-oxime derivatives, showing that the *E*-isomer consistently has the lower ground-state energy. The calculated energy barriers indicate that uncatalyzed thermal or photochemical isomerization is slow in standard organic solvents, preserving stereochemical integrity during initial biological screening. However, it is important to note that aqueous environments, particularly under acidic conditions or within specific enzymatic microenvironments, can significantly lower the activation barrier for *E*/*Z* isomerization of oximes through water-assisted catalytic mechanisms [80].

**Scheme 6:**
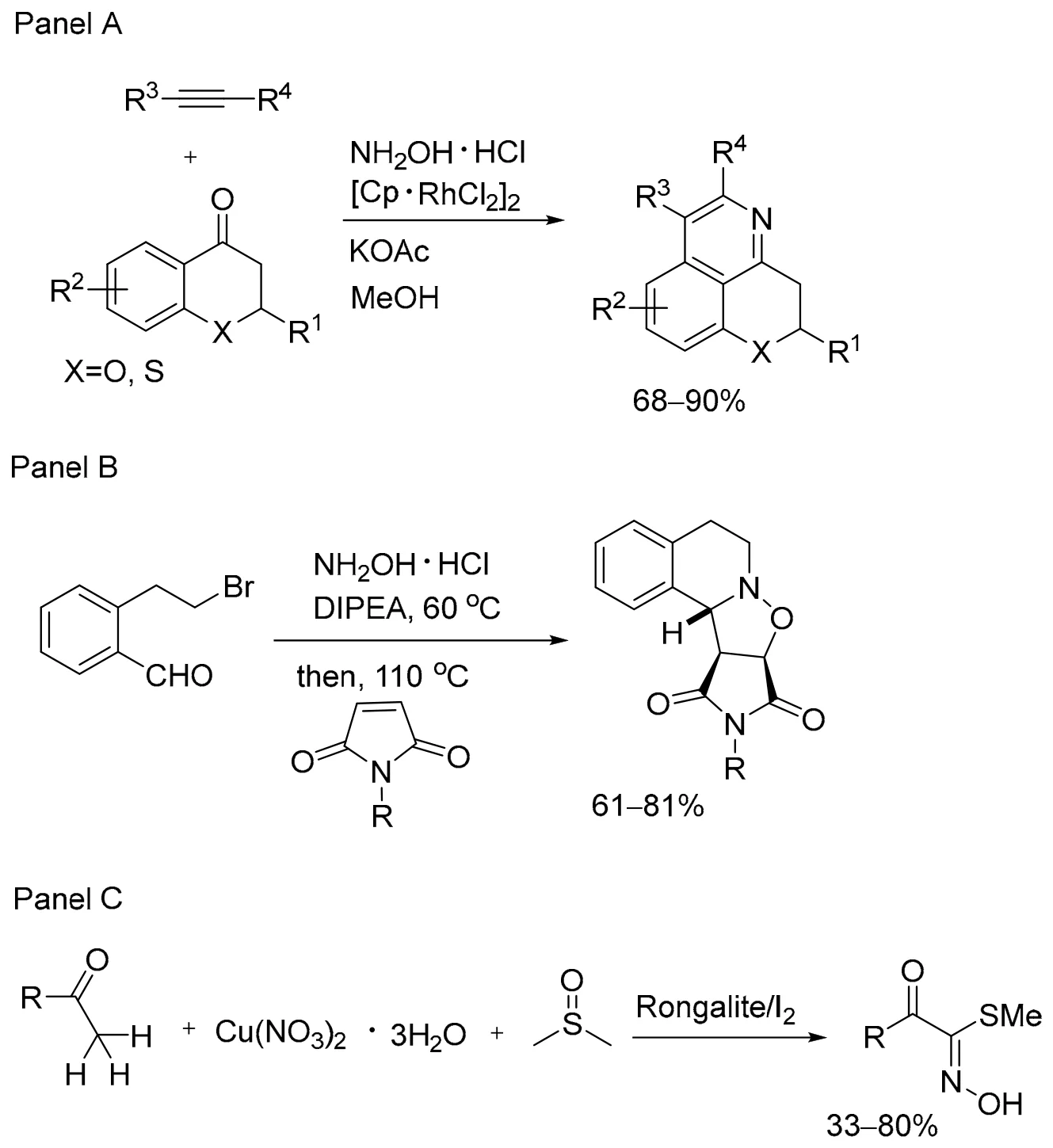
Oximation-based synthetic methodologies (Panels **A**,**B**) and rongalite/iodine-mediated synthesis of oximes (Panel **C**).

Recent synthetic studies likewise point to the thermodynamic stability of the *E*-configured oxime bond. Cascade oximation reactions, often combined with C–H activation or dipolar cycloadditions, have been shown to proceed with high stereoselectivity, presumably because formation of the *E*-oxime product provides the thermodynamic driving force [48,49] (Scheme 6A,B). Newer oxime-forming methods reinforce this conclusion. In particular, rongalite/iodine-mediated C(sp^3^)–H bond oximation using copper nitrate as a [NO] reagent [43] demonstrates that the oxime group can be introduced with maintaining predictable geometry (Scheme 6C).

Viewed through the lens of synthetic efficiency (Scheme 6), these cascade oximation and post-modification reactions show high atom, step, and redox economy [49]. Their multicomponent design shortens synthetic sequences and broadens substrate scope, allowing fused-ring skeletons and diverse functional groups to be incorporated under mild conditions [43,48].

The spatial arrangement of the oxime pharmacophore is important for target recognition and binding affinity. Molecular docking and enzymatic inhibition studies demonstrate that the oxime group configuration influences positions of the N,O-motif and the hydroxyl proton for complementary interactions within kinase active sites. Schepetkin et al. [20] established that 11*H*-indeno[1,2-*b*]quinoxalin-11-one oxime and tryptanthrin-6-oxime function as high-affinity inhibitors of JNK1-3, with binding modes heavily dependent on precise hydrogen-bonding interactions between the oxime group and key catalytic residues. Furthermore, the *E* and *Z* isomers of IQ-1 exhibit distinct pharmacokinetic profiles, as demonstrated by a validated LC-MS/MS method that quantified them separately in rat plasma after oral administration [81].

Thus, the *E*-isomer is now established as the thermodynamically stable form of 11*H*-indeno[1,2-*b*]quinoxalin-11-one oxime and tryptanthrin-6-oxime. As this geometry influences both target binding and pharmacokinetics, it must be considered in the design and evaluation of these compounds.

## 3. Biological Activity of the Tetracyclic Ketoximes and Related Compounds

### 3.1. Neuroprotective Effects of the Oximes

The neuroprotective efficacy of tetracyclic ketoximes is primarily attributed to their ability to modulate stress-dependent signaling cascades that regulate neuronal survival. In particular, certain tetracyclic ketoximes selectively inhibit JNK, the expression of which plays an important role in ischemic stroke and progressive neurodegenerative diseases [82,83]. The JNK3 isoform is predominantly expressed in the central nervous system (CNS) and plays a critical role in the pathogenesis of excitotoxicity, neuroinflammation, ischemic stroke, and progressive neurodegenerative disorders such as AD [84,85]. The incorporation of the exocyclic oxime pharmacophore in a rigid, planar tetracyclic scaffold provides a unique structural feature that enables high-affinity, isoform-selective binding to the ATP pocket of JNK. This interaction effectively suppresses the downstream phosphorylation of c-Jun and other pro-apoptotic substrates [86]. Consequently, compounds such as IQ-1, its sodium (IQ-1S) and lithium (IQ-1L) salts, and tryptanthrin-6-oxime have demonstrated potent JNK inhibition coupled with robust neuroprotective activity across multiple preclinical models of acute and chronic CNS injury [44,87].

In experimental models of acute cerebral ischemia, these oxime derivatives reliably attenuate infarct expansion and accelerate functional neurological recovery. For instance, in a rat model of transient focal cerebral ischemia (FCI) induced by middle cerebral artery occlusion (MCAO), the intraperitoneal administration of tryptanthrin-6-oxime at doses of 5–10 mg/kg significantly reduced neurological deficit scores by 35–67% and decreased infarct volume by ~30% at 48 h post-reperfusion [88]. Comparative studies have further elucidated that, while both the parent tryptanthrin and its oxime derivative reduce cerebral infarction size by approximately 24–30% and mitigate brain tissue swelling by over 60%, the oxime moiety imparts enhanced anti-edematous and anti-inflammatory potency [89]. Similarly, IQ-1S administered at 5–25 mg/kg in focal ischemia models reduced infarct volumes by 20–50%, achieving neuroprotective efficacy comparable to the clinically utilized neuroprotector citicoline [87]. Notably, the lithium salt derivative, IQ-1L, exhibited superior therapeutic potency, producing a 52% reduction in infarct size at a relatively lower dose (12 mg/kg). This enhanced efficacy is likely attributable to Li^+^-mediated stabilization of the oximate coordination geometry, which significantly improves JNK1-3 binding affinity in vitro [90]. These favorable outcomes directly correlate with the suppression of c-Jun phosphorylation in the ischemic penumbra and hippocampal regions, confirming a sustained, targeted effect under conditions of severe pathology [87,88].

**Figure 3:**
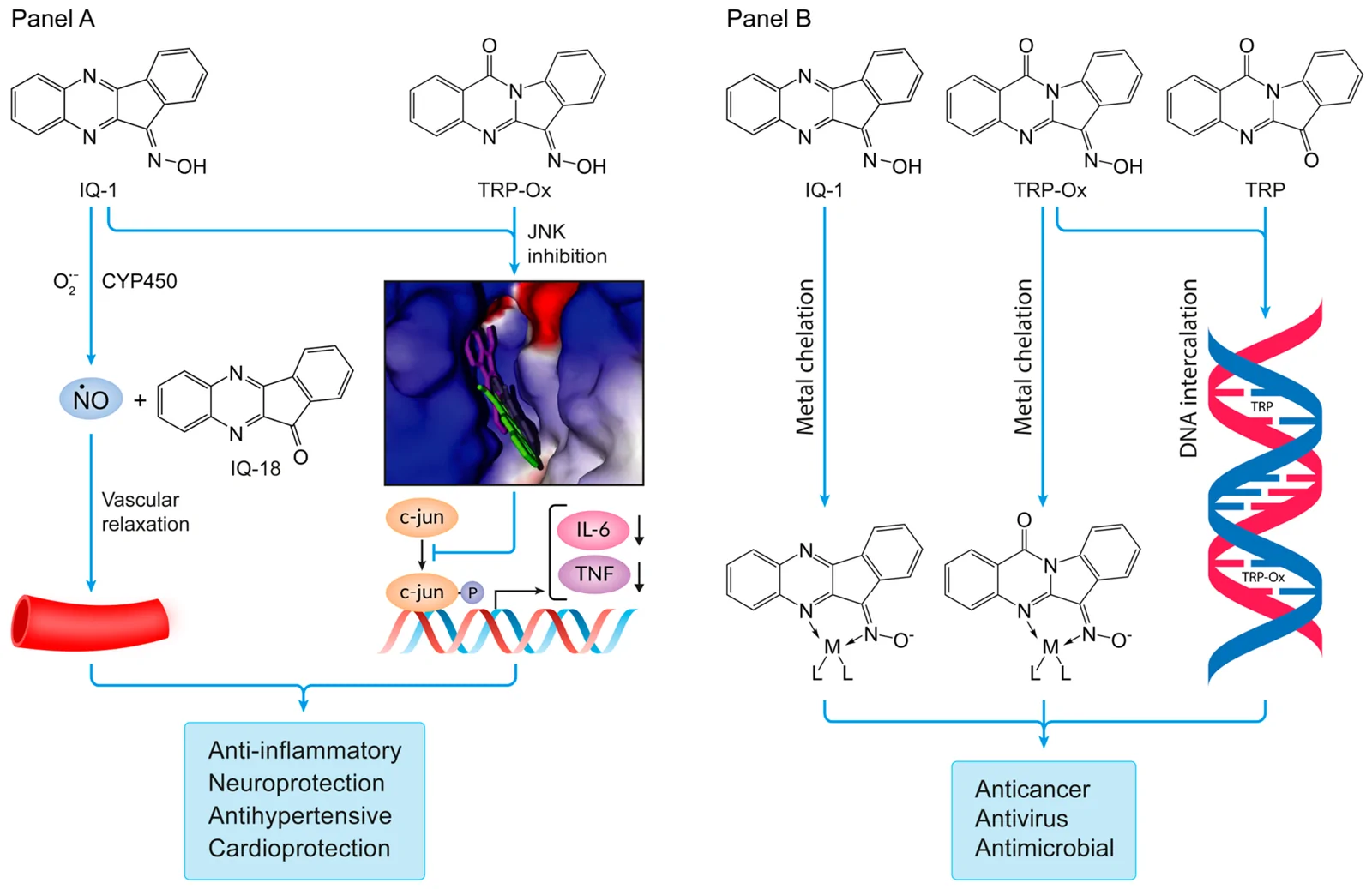
Schematic representation of the main biological effects of IQ-1, tryptanthrin-6-oxime (TRP-Ox), and tryptanthrin (TRP). Panel (**A**). JNK inhibitors IQ-1 and tryptanthrin-6-oxime (TRP-Ox) block activity of JNK; IQ-1 undergoes cytochrome P450-catalyzed oxidation to nitric oxide (NO·) and inactive ketone (IQ-18) in liver microsomes. These effects can provide anti-inflammatory, neuroprotective, antihypertensive, and cardioprotective actions, depending on the biological model and the administration regimen. Panel (**B**). The oxime-based chelating pharmacophore is capable of coordinating with transition metals, while the compact tetracyclic core intercalates into DNA. These effects can provide anticancer, antivirus or antimicrobial actions.

Beyond direct kinase inhibition, tetracyclic ketoximes exert potent anti-inflammatory and antioxidant effects that synergistically preserve neuronal architecture and vascular integrity. Following an ischemic insult, IQ-1S and tryptanthrin-6-oxime significantly downregulate the production of pro-inflammatory cytokines, including interleukin-1β (IL-1β) and tumor necrosis factor (TNF), within both the ischemic core and the contralateral hemisphere [88,89]. Tryptanthrin-6-oxime also attenuates cerebral edema and reduces ROS generation in brain homogenates, demonstrating direct cytoprotective activity against H_2_O_2_-induced oxidative stress in SH-SY5Y neuroblastoma cells [18,88]. In a global cerebral ischemia (three-vessel occlusion) model, IQ-1S administration not only preserved pyramidal neurons in the hippocampal CA1 region but also improved local cerebral blood flow by 60%, reduced blood hyperviscosity, and mitigated endothelial dysfunction, highlighting pleiotropic vascular and neuroprotective mechanisms [91]. These anti-inflammatory mechanisms are highly likely to be retained or even amplified by oxime functionalization. Moreover, the dual ability of the IQ-1S compound to release NO and inhibit JNK (Figure 3A), properties first demonstrated in a mouse MCAO model, highlights the multifaceted mechanisms of action of the oxime pharmacophore, enabling it to target both hemodynamic and molecular components of stress in ischemic neuropathology [17]. Indeed, endothelial dysfunction develops in a number of CNS disorders; one of its manifestations is the inhibition of endothelial NO synthase (eNOS) activity and NO deficiency [92,93]. Under these conditions, the NO pool generated during the biotransformation of tetracyclic ketoximes [16] can, to a certain extent, compensate for the deficit of endogenous NO (for details, see Section 3.2.3).

In a rat model of focal transient ischemia, tryptanthrin at a dose of 12 mg/kg significantly reduced the severity of neurological deficits and infarct volume, lowered IL-1β and TNF levels in the cerebral infarct zone, and decreased blood–brain barrier (BBB) permeability and edema in the supraventricular region of the affected hemisphere [94]. Furthermore, tryptanthrin has been shown to drive microglial polarization from the neurotoxic, pro-inflammatory M1 phenotype toward the neuroprotective M2 phenotype via the inactivation of the cyclic GMP-AMP synthase (cGAS)/stimulator of interferon genes (STING)/nuclear factor-κB (NF-κB) axis, thereby promoting functional recovery in spinal cord injury models [95]. Consistent with these findings, tryptanthrin suppresses Toll-like receptor 4 (TLR4)-MyD88-mediated NF-κB and mitogen-activated protein kinase (MAPK) p38 activation in lipopolysaccharide (LPS)-stimulated BV2 microglial cells, as well as activating the Nrf2/heme oxygenase-1 (HO-1) antioxidant pathway [96,97].

The neuroprotective scope of tetracyclic ketoximes extends significantly beyond acute ischemic injury to encompass chronic neurodegenerative and age-related pathologies. In senescence-accelerated OXYS rats, which spontaneously develop AD-like pathology and age-related macular degeneration (AMD), the chronic administration of IQ-1S significantly suppressed JNK activity. This intervention reduced amyloid precursor protein (APP) and tau phosphorylation in the hippocampus, thereby interrupting the proteostatic collapse and neuroinflammatory cascades that drive progressive neuronal loss [98]. In the context of AMD, IQ-1S treatment improved retinal blood circulation, enhanced retinal pigment epithelium function, reduced vascular endothelial growth factor (VEGF) expression, and increased pigment epithelium-derived factor (PEDF), collectively mitigating photoreceptor degeneration and preserving retinal synapses [99,100].

Building upon the success of JNK inhibition, recent structural diversification of the indoloquinoxaline and related analogs has yielded novel MTDLs specifically tailored for AD therapy. For instance, novel tryptanthrin derivatives bearing benzenesulfonamide substituents have been developed as potent, mixed reversible dual inhibitors of AChE (IC_50_ = 0.13 μM) and BuChE (IC_50_ = 6.1 μM), while simultaneously preventing self-induced amyloid-β (Aβ) aggregation and suppressing neuroinflammation [24]. Similarly, targeted modifications have produced highly selective AChE inhibitors (e.g., selectivity index = 517 over BuChE) that not only inhibit Aβ aggregation but also demonstrate robust neuroprotective and metal-chelating properties, effectively reversing learning and memory impairments in scopolamine-induced AD mouse models [25]. Furthermore, in silico and in vitro screening of spiro-indenoquinoxaline–pyrrolidine hybrids has identified promising antiamyloidogenic candidates with high binding affinities for both monomeric and oligomeric Aβ peptides, supported by stable molecular dynamics simulations [101].

The broader utility of the oxime pharmacophore in neuroprotection is also evident in related heterocyclic systems. Imidazolylacetophenone oxime derivatives have shown potent anti-neuroinflammatory effects, antioxidative properties, and AChE-inhibitory effects, successfully overcoming the BBB to exert neuroprotection in models of ferroptosis and 6-hydroxydopamine-induced cell damage [102,103]. In particular, Ren et al. reported the protective effect of an imidazolylacetophenone oxime against H_2_O_2_-induced damage and ferroptosis in PC12 cells and demonstrated the compound’s selective chelating properties toward Cu^2+^ ions [103]. Given that metal ions, specifically copper, iron, and zinc, play a key role in the pathogenesis of AD by influencing Aβ deposition and tau protein accumulation [104], the chelating properties of oximes could be utilized in a novel therapeutic strategy for treating the disease. Additionally, the related compound indirubin-3’-oxime has been formulated into solid lipid nanoparticles (size ~186 nm) to enhance brain-targeted delivery. These nanoparticles demonstrated robust neuroprotection in okadaic acid-induced tauopathy models via the modulation of the glycogen synthase kinase-3 β (GSK-3β)/Wnt signaling pathway, significantly reducing Aβ_1–42_ and p-tau levels [105].

The clinical translation of these neuroprotective effects is heavily dependent on pharmacokinetic optimization and efficient BBB permeability [106]. Numerous neuroprotectors showed promise for treatment of ischemic stroke, but their delivery to the brain remained challenging [107]. The ability of tryptanthrin to cross the BBB was previously evaluated in several in vitro human and animal BBB models [108]. Computational ADME profiling consistently predicts high CNS penetration for IQ-1 and tryptanthrin [94], which is corroborated by in vivo pharmacokinetic studies, demonstrating extensive tissue distribution. Following intravenous administration in rats and rabbits, IQ-1 exhibits a two-compartment pharmacokinetic profile with high clearance values and a steady-state volume of distribution exceeding total body water, indicating rapid CNS penetration and broad systemic exposure [109]. However, the oral bioavailability of the free oxime remains low (<1.5%), suggesting that targeted formulation strategies or prodrug approaches are required to optimize systemic exposure [110].

Collectively, the robust in vivo efficacy, favorable CNS pharmacokinetics, and multi-targeted mechanisms, spanning JNK3 inhibition, anti-inflammatory/antioxidant signaling, AChE inhibition, and anti-amyloidogenic activity, establish tetracyclic ketoximes and their derivatives as a highly promising class of neuroprotective therapeutics. These findings warrant further structural optimization, advanced nanocarrier formulation development, and rigorous clinical evaluation for the treatment of ischemic stroke and progressive neurodegenerative disorders. Table 1 provides a consolidated overview of the key neuroprotective findings for these oxime derivatives across preclinical models.

### 3.2. Cardioprotective and Antihypertensive Effects of the Oximes

The therapeutic potential of tetracyclic ketoximes regarding the cardiovascular system is inextricably linked to their highly synergistic dual pharmacological profile: high-affinity inhibition of stress-activated JNK kinases combined with the oxime group’s inherent ability to act as an endogenous NO donor. Pathological cardiovascular conditions, including resistant hypertension, myocardial ischemia–reperfusion (I/R) injury, and post-infarction ventricular remodeling, are heavily driven by JNK-mediated inflammatory cascades, endothelial dysfunction, and severe oxidative stress. The strategic incorporation of the oxime pharmacophore onto rigid indenoquinoxaline and tryptanthrin scaffolds has yielded a unique class of multi-target cardiovascular agents capable of simultaneously attenuating maladaptive molecular signaling and restoring physiological vascular tone.

#### 3.2.1. Antihypertensive Efficacy and Vascular Remodeling

Evidence suggests that JNKs are involved in the pathogenesis of elevated vascular tone in essential hypertension [111]. As essential factors in the increase in blood pressure in hypertension [112,113], angiotensin II and endothelin-1 partially mediate their effects through the activation of JNK [114,115]. In hypertension, activation of the JNK signaling pathway triggers pathological myocardial and aortic remodeling [116,117]. Emerging data suggest that antihypertensive drugs affect the regulation of the JNK signaling pathway [118,119].

Chronic administration of the sodium salt of IQ-1 (IQ-1S) has demonstrated robust and sustained antihypertensive activity in spontaneously hypertensive rats (SHRs). In a comprehensive 6-week regimen, daily intragastric dosing of IQ-1S (5–50 mg/kg) significantly reduced systolic and mean arterial blood pressure, alongside a marked decrease in total peripheral resistance, hematocrit, and whole-blood viscosity [120]. Importantly, these hemodynamic improvements were accompanied by a beneficial effect on the cellular structure of the myocardium. IQ-1S treatment directly attenuated pressure-induced hypertrophic remodeling, as evidenced by a significant reduction in myocardial cell cross-sectional area and aortic wall thickness.

Mechanistically, IQ-1S exerts concentration-dependent vasorelaxation in isolated carotid arterial rings and significantly suppresses the secretion of the potent vasoconstrictor endothelin-1 from human umbilical vein endothelial cells (HUVECs) in vitro. This suggests a dual action on both vascular smooth muscle tone and endothelial secretory function [120]. Furthermore, IQ-1S effectively mitigates the progression of diastolic dysfunction in SHRs with established, stable hypertension. Treatment at 50 mg/kg reduced left ventricular end-diastolic pressure (LVEDP) by 40% and decreased the left ventricular mass-to-body mass index by 5%, confirming its capacity to preserve diastolic compliance and delay the transition to overt heart failure [121].

#### 3.2.2. Cardioprotection in Acute Ischemia and Chronic Post-Infarction Remodeling

Recent studies have shown that the JNK pathway is involved in myocardial I/R injury [122,123]. JNK plays an important role in the regulation of inflammation, apoptosis, necrosis, and several transcriptional and non-transcriptional processes involved in cardiomyocyte injury during I/R [124]. The contribution of the JNK pathway to post-infarction heart failure pathogenesis has been convincingly demonstrated in experimental animals and in cell cultures [125]. Beyond systemic blood pressure modulation, the tetracyclic ketoximes exhibit potent cardioprotective effects in models of acute myocardial I/R injury and chronic post-infarction remodeling. In a rat model of acute myocardial ischemia (1 h) followed by reperfusion, a single intraperitoneal dose of the free oxime IQ-1 (25 mg/kg) administered during the ischemic phase reduced the infarct area from 24 to 14% at 16 h post-reperfusion [126]. A prolonged 5-day course of IQ-1 further restricted the infarct zone to just 10% and significantly improved left ventricular cardiodynamics. Systolic pressure increased by 20%, maximum rates of pressure rise (+dP/dt_max_) and fall (−dP/dt_max_) improved by 23% and 43%, respectively, and minimum diastolic pressure decreased 3.4-fold [126,127].

These acute hemodynamic benefits translate directly into long-term structural preservation. When tryptanthrin-6-oxime was administered at 12 mg/kg intraperitoneally during the acute phase of myocardial infarction and continued for 4 days, it profoundly attenuated fibrotic scarring over a 4-month follow-up period. Treated animals exhibited a relative increase in viable muscle elements within the scar, a greater than 50% reduction in connective tissue replacement, and decelerated fibrosis in remote, non-infarcted myocardial regions [128]. Consequently, long-term systemic and cardiohemodynamic parameters were significantly preserved, with marked increases in stroke volume and cardiac output.

#### 3.2.3. The Dual Mechanism: JNK Inhibition and NO Donation

The marked cardiovascular efficacy of these oximes appears to arise from a coordinated dual mechanism. JNK inhibition attenuates pro-apoptotic signaling, inflammatory cytokine release, and pathological hypertrophy, whereas the oxime moiety can itself function as a direct vasodilatory element through NO release (Figure 3).

The formation of NO during the oxidation of the oxime group has been demonstrated for a broad class of compounds containing the C=N–OH function and is theoretically determined solely by the compound’s susceptibility to microsomal oxidation [129]. Formaldoxime and *E*-cinnamaldehyde oxime have been reported to induce potent endothelium-independent vasorelaxation. The effect is mediated through a 7-ethoxyresorufin-sensitive NAD(P)H-dependent reductase pathway, in which oxime metabolism releases NO, leading to soluble guanylyl cyclase (sGC) activation, increased cyclic GMP (cGMP), and the opening of potassium channels [130,131,132]. To date, NO formation during the oxidation of tetracyclic ketoximes has been experimentally demonstrated only for the compound IQ-1. Using electron paramagnetic resonance (EPR) spectroscopy with diethyldithiocarbamate (DETC) and hemoglobin spin traps, Andrianov et al. [16] have shown that IQ-1 undergoes oxidoreductive bioconversion in vivo, generating NO in both hepatic tissue and the systemic circulation. DFT calculations further indicate that NO formation from IQ-1 through interaction with the superoxide anion radical (·O_2_^−^) is thermodynamically favorable, supporting the possibility of a sustained, enzyme-independent source of vasodilatory signaling. For tetracyclic ketoximes, such localized reductase-mediated NO release likely works in parallel with JNK-dependent suppression of vascular inflammation. Together, these actions create a favorable hemodynamic profile characterized by reduced ventricular afterload, improved coronary perfusion, and limitation of ischemic necrosis.

#### 3.2.4. Modulation of Vascular Inflammation and Endothelial Function

Acute and chronic cardiovascular diseases are often worsened by low-grade vascular inflammation and endothelial dysfunction [133,134]. After myocardial infarction, for instance, an acute inflammatory response develops during the first several days [135]. If inflammation persists, it can promote adverse left ventricular remodeling after myocardial infarction, which makes inflammatory signaling an important therapeutic target for improving post-infarction outcome [136].

JNKs are central regulators of the inflammatory processes that contribute to cardiomyocyte injury during I/R [137]. Tetracyclic ketoximes, especially tryptanthrin derivatives, suppress JNK-dependent activation of transcription factors such as NF-κB and activating protein-1 (AP-1). This inhibition leads to a marked downregulation of pro-inflammatory mediators, including IL-6, TNF, and monocyte chemoattractant protein-1 (MCP-1) [19]. Through suppression of these inflammatory cascades, oximes may reduce endothelial activation and limit leukocyte recruitment to the vascular wall. The parent compound tryptanthrin has also been shown to regulate the phenotypic switching of vascular smooth muscle cells (VSMCs) through the AMP-activated protein kinase (AMPK)/acetyl-CoA carboxylase (ACC) signaling pathway. By preventing VSMCs from shifting from a contractile phenotype to a synthetic, proliferative state, tryptanthrin significantly reduces arterial intimal hyperplasia and atherosclerotic plaque progression in ApoE^−/−^ mice [138]. In line with this anti-inflammatory profile, Kawaguchi et al. [139] reported that tryptanthrin suppresses polyinosinic–polycytidylic acid-induced CXCL10 expression in HUVECs by attenuating signal transducer and activator of transcription 1 (STAT1) phosphorylation and nuclear translocation, thereby confirming anti-inflammatory activity at the level of the vascular endothelium.

Table 2 consolidates the key cardioprotective and antihypertensive findings for tetracyclic ketoximes and related tryptanthrins.

#### 3.2.5. Antithrombotic and Vascular Remodeling-Related Activities

Recent work has broadened the cardiovascular significance of tryptanthrin to include hemostasis and angiogenesis. Gao et al. [140] showed that tryptanthrin markedly impairs platelet function and suppresses thrombus formation in mouse models of FeCl_3_-induced carotid artery thrombosis and deep venous thrombosis. Mechanistically, this antithrombotic activity is associated with a downregulation of glycoprotein Ibα (Gp1bα), together with broad suppression of platelet activation and fibrin clotting cascades. Tryptanthrin also displays strong anti-angiogenic activity. In human microvascular endothelial cells, it inhibits angiogenesis by targeting the vascular endothelial growth factor receptor 2 (VEGFR2)-mediated extracellular signal-regulated kinase 1 and 2 (ERK1/2) signaling pathway [141]. Later studies showed that this anti-angiogenic mechanism also involves substantial downregulation of the pro-angiogenic factor apelin, achieved through reduced promoter activity and decreased mRNA stability [144]. In a related cardiovascular context, 8-methyltryptanthrin (8-Me-TRP) has been reported to promote differentiation of P19CL6 embryonal carcinoma cells into spontaneously beating cardiomyocyte-like cells by activating gene expression programs for pacemaker channels (Ca_v1.2, Ca_v3.1) and gap junction proteins (GJA1, GJA5) [143]. The cardioprotective and antihypertensive profiles of tetracyclic ketoximes unite targeted kinase inhibition with endogenous vasodilator release. IQ-1, IQ-1S, and tryptanthrin-6-oxime simultaneously shrink infarct size, limit diastolic dysfunction, slow post-infarction fibrosis, and lower systemic vascular resistance, revealing clear multi-modal therapeutic potential. Long-term safety profiling, gains in oral bioavailability through advanced prodrug or salt formulations, and rigorous testing in clinically relevant models of heart failure with preserved ejection fraction (HFpEF) and resistant hypertension now stand as the principal priorities. This dual-action logic supplies a practical framework for next-generation cardiovascular agents able to address both hemodynamic overload and molecular stress signaling.

### 3.3. Anti-Inflammatory and Immunomodulatory Effects

Tetracyclic ketoximes owe their anti-inflammatory efficacy to the ability to intercept stress-activated signaling cascades and thereby interrupt the transcriptional networks that drive both acute and chronic inflammatory pathologies. The parent tryptanthrin core already displays baseline immunomodulatory activity; installation of the exocyclic oxime pharmacophore markedly strengthens engagement with specific kinase catalytic pockets. That modification converts the rigid tetracyclic scaffold into a potent multi-target anti-inflammatory agent capable of suppressing cytokine storms, reprogramming immune-cell polarization, and limiting tissue damage across diverse preclinical models.

#### 3.3.1. Anti-Inflammatory and Immunomodulatory Effects of Tryptanthrin

A comprehensive assessment of the pharmacological profile of oxime derivatives should begin with an examination of the pronounced anti-inflammatory activity inherent in their basic structure. Tryptanthrin, originally isolated from traditional medicinal plants such as *I. tinctoria* and *P. tinctorium*, was recognized early as a potent dual inhibitor of the arachidonic acid cascade. In vitro work showed selective COX-2 inhibition (IC_50_ = 64 nM), a potency on par with the clinical NSAID nimesulide, together with concurrent 5-lipoxygenase (5-LOX) inhibition (IC_50_ = 0.6 µM) in human neutrophils [145,146]. The compound is neither redox-active nor a direct inhibitor of 5-LOX in cell-free assays. Instead, it alters the enzyme’s subcellular localization and thereby suppresses leukotriene B4 (LTB_4_) formation in whole blood [147]. Tryptanthrin also potently downregulates iNOS expression, producing dose-dependent drops in NO and prostaglandin E_2_ (PGE_2_) output from activated macrophages [148]. The resulting multi-pronged blockade of eicosanoid pathways, therefore, precedes, and later complements, the kinase-directed actions conferred by oxime functionalization.

#### 3.3.2. Kinase-Dependent Transcriptional Regulation

Converting the exocyclic carbonyl group to an oxime shifts the dominant pharmacological focus of these compounds toward the JNK family. IQ-1S and its neutral analogue, IQ-1, strongly inhibit LPS-induced secretion of IL-1α, IL-1β, IL-6, IL-10, TNF, interferon-γ (IFN-γ), and granulocyte-macrophage colony-stimulating factor (GM-CSF) in murine macrophage and human monocytic cell lines [19]. This cytokine suppression is mediated, at least in large part, by inhibition of JNK-dependent activation of the transcription factors NF-κB and AP-1. Binding studies further show that the oxime derivatives IQ-1 and IQ-1S interact with high affinity at the catalytic sites of JNK1 and JNK3, with dissociation constants (K_d_) ranging from 22 to 490 nM [20]. Of particular interest, modification at the 8-position of the tryptanthrin core produces oxime derivatives with marked selectivity for JNK3 over JNK1/2, a feature that may reduce the off-target effects associated with the broader physiological functions of JNK1 [86].

Structural diversification of tryptanthrin derivatives also extends their activity beyond JNK to other inflammatory kinases. Benzenesulfonamide-substituted tryptanthrin derivatives, for example, selectively target p38α MAPK, suppressing NO, IL-1β, and TNF production while stabilizing p38α in cellular thermal shift assays [149]. Recent work also indicates that tryptanthrin downregulates oncostatin M, a key cytokine in chronic inflammation, by acting on the GM-CSF-mediated phosphoinositide 3-kinase (PI3K)-Akt-NF-κB signaling axis in neutrophils [150].

#### 3.3.3. Modulation of Innate Immunity, Inflammasomes, and Toll-like Receptors (TLRs)

Tetracyclic ketoximes and their parent precursors also interfere with innate immune sensing and inflammasome assembly. Tryptanthrin functions as a broad-spectrum inflammasome inhibitor by binding stably to the pyrin domain of apoptosis-associated speck-like protein containing a CARD (ASC PYD). This interaction disrupts ASC oligomerization and subsequent caspase-1 maturation, thereby attenuating activation of the NLRP3, NLRC4, and AIM2 inflammasomes [27]. Tryptanthrin also modulates several TLR-dependent pathways. In THP-1 macrophages and HUVECs, it suppresses TLR3 activation induced by polyinosinic–polycytidylic acid (poly IC), markedly reducing STAT1 phosphorylation and nuclear translocation. As a result, expression of CXCL10 and classical interferon-stimulated genes (ISGs) is downregulated, while the primary antiviral IRF3/IFN-β axis remains intact [139,151]. In LPS-stimulated models, tryptanthrin inhibits the TLR4/MyD88/ROS/NF-κB and JAK/STAT3 signaling axes and, at the same time, activates the Keap1/Nrf2 antioxidant defense pathway. These combined effects support a dual mechanism involving inflammatory suppression and the preservation of redox homeostasis [97,152].

#### 3.3.4. Reprogramming of Macrophage Polarization and Adaptive T-Cell Responses

Beyond inhibiting transcriptional signaling, these compounds also alter the functional state of immune cells. In murine models of LPS-induced sepsis and pressure ulcers, IQ-1S or tryptanthrin treatment markedly reduces systemic pro-inflammatory cytokine levels and shifts macrophage polarization away from the neurotoxic/inflammatory M1 phenotype toward the tissue-reparative M2 phenotype. This phenotypic transition is associated with decreased iNOS expression, increased Arg1 (arginase-1)/MRC1 (mannose receptor C-1) expression, and inactivation of the cGAS/STING/TBK1 signaling pathway [153,154].

IQ-1S reduces the activation of CD4^+^ and CD4^−^ T cells, preferentially suppressing central memory and effector memory subsets, while decreasing the secretion of IL-2, IFN-γ, and IL-4 [155]. Importantly, the indoloquinazoline core is associated with potent inhibition of T helper 17 (Th17) cell polarization. Tryptanthrin and related indole alkaloids from Indigo naturalis potently decrease the expression of IL-17A, CCL20, and the antimicrobial peptide S100A9 in RORγt-expressing T cells and activated keratinocytes [156,157]. Conversely, in allergic paradigms, tryptanthrin inhibits the expression of Th2-specific transcription factors, c-Maf and GATA-3, while simultaneously blocking IgE-mediated degranulation and IL-4 production in basophilic leukemia cells [158]. Furthermore, in autoimmune arthritis models, IQ-1S treatment increases the population of Foxp3^+^CD4^+^CD25^+^ regulatory T cells (T_regs_) in draining lymph nodes, promoting an immunosuppressive, tolerance-inducing microenvironment [159].

#### 3.3.5. In Vivo Efficacy in Autoimmune, Gastrointestinal, and Dermatological Pathologies

The translational potential of tetracyclic ketoximes is robustly demonstrated across diverse preclinical models. In murine collagen-induced arthritis (CIA) and collagen-antibody-induced arthritis (CAIA), both tryptanthrin and tryptanthrin-6-oxime significantly attenuate joint swelling, cartilage degradation, and clinical arthritis scores. While both compounds reduce collagen II-specific antibody titers, tryptanthrin-6-oxime consistently exhibits superior in vitro potency in suppressing IL-17A, GM-CSF, and receptor activator of NF-κB ligand (RANKL), likely attributable to its enhanced JNK inhibitory profile [159,160].

In the context of inflammatory bowel disease (IBD), tryptanthrin demonstrates remarkable mucosal protection. In dextran sulfate sodium (DSS)-induced colitis models, tryptanthrin administration prevents weight loss, reduces disease activity indices, and preserves colon length. Magnetic resonance imaging and histopathological analyses confirm a significant reduction in bowel wall edema, fibrosis, and goblet cell loss, driven by the inhibition of the TNF/NF-κB and IL-6/STAT3 pathways, as well as the modulation of the Aryl hydrocarbon receptor (AhR) by the compound [161,162,163,164,165].

Dermatological applications represent another highly validated therapeutic avenue. In imiquimod-induced psoriasis models, tryptanthrin ameliorates skin lesions by suppressing NF-κB/MAPK signaling, activating Nrf2, and reducing Th17 cell infiltration [166]. The clinical relevance of this mechanism was confirmed in a randomized, double-blind, placebo-controlled trial where topical Indigo naturalis ointment (containing tryptanthrin) achieved a PASI 75 response in 56% of moderate psoriasis patients, correlating with a transcriptomic downregulation of the IL-17 pathway [167]. In atopic dermatitis models, tryptanthrin downregulates thymic stromal lymphopoietin (TSLP) production in mast cells via the RIP2/caspase-1/NF-κB pathway, ameliorating clinical atopic dermatitis symptoms and reducing serum histamine and IL-4 levels [168,169]. Topical preparations, specifically the dermo-protective agent Kourochitin, which contains tryptanthrin as its active ingredient, have further confirmed the antiallergic and wound-healing properties of this compound in mouse and dog models of dermatitis [170].

Beyond the skin and gut, the anti-fibrotic and barrier-enhancing properties of tryptanthrin have been demonstrated in bleomycin-induced pulmonary fibrosis, where the compound suppresses both MAPK/NF-κB and TGF-β1/Smad signaling [171] and in gingival epithelial cells, where it inhibits osteoclastogenesis and upregulates tight junction proteins, suggesting utility in periodontitis [172].

#### 3.3.6. Advanced Delivery Systems and Structural Diversification

Given the pharmacokinetic constraints associated with systemic administration, recent studies have incorporated tetracyclic ketoximes into advanced drug delivery systems designed to provide localized and sustained anti-inflammatory activity. Electrospun poly(ε-caprolactone) (PCL) scaffolds and electrosprayed poly(lactic-co-glycolic acid) (PLGA) microparticles loaded with IQ-1 show controlled, diffusion-mediated release profiles. These biomaterials inhibit neutrophil activation, reduce LPS-induced IL-6 production, and limit adverse foreign-body immune responses without compromising cell viability [173,174].

Tryptanthrin has also been advanced through biomimetic structural diversification. Under acidic, biomimetic conditions, tryptanthrin reacts selectively with endogenous indole monoamines, such as tryptamine and serotonin, to generate spirocyclic adducts. These spirocyclic products retain strong anti-inflammatory activity, significantly decreasing IL-1β secretion associated with inflammasome activation in macrophages, while preserving favorable passive membrane permeability [175].

Table 3 provides a consolidated overview of the key anti-inflammatory and immunomodulatory findings for tetracyclic ketoximes and structurally related tryptanthrin, including compound identity, preclinical model, dosing regimen, principal outcomes, and the proposed mechanisms of action.

### 3.4. Anti-Tumor Effects of the Oximes and Related Compounds

Tetracyclic ketoximes have become an increasingly important focus in oncology drug discovery because of their antiproliferative and pro-apoptotic activities. The parent indenoquinoxaline and tryptanthrin ketones possess intrinsic cytotoxicity. However, conversion of the exocyclic carbonyl group to an oxime moiety markedly enhances antitumor activity across a range of cancer models, including chemoresistant systems [10,15]. This improvement is largely linked to the distinctive stereoelectronic properties of the oxime group. By modulating the electronic structure, the oxime functionalization can favor deeper DNA intercalation; it also introduces a strong chelating motif suitable for transition metal coordination (Figure 3B). As a result, tetracyclic ketoximes and their derivatives are now regarded as privileged scaffolds capable of engaging several oncogenic targets, including topoisomerases, telomerase, and receptor tyrosine kinases (RTK). This multi-target profile provides a promising framework for addressing established mechanisms of tumor resistance.

#### 3.4.1. Oxime Derivatives and Cytotoxic Profiling

Systematic SAR studies of indeno[1,2-*c*]quinoline and indenoquinoxaline scaffolds have shown that C-11 oxime functionalization provides clear advantages over the corresponding ketone precursors in both potency and selectivity. Tseng et al. [178] reported that the hydroxyimino derivative of 9-methoxy-11*H*-indeno[1,2-*c*]quinolin-11-one displayed stronger antiproliferative activity than its carbonyl analogue. Subsequent introduction of aminoalkoxyimino side chains produced compounds with submicromolar GI_50_ values against camptothecin-resistant lung (A549) and gastric (AGS) carcinoma cell lines.

Later SAR work further indicated that terminal tertiary amines or pyrrolidino rings attached to the oxime oxygen are important for improving cellular uptake and stabilizing target engagement in the acidic tumor microenvironment [179]. For example, the *O*-2-(pyrrolidin-1-yl)ethyl oxime derivative showed potent cytotoxicity against SAS, A549, and BT483 cell lines (GI_50_ = 0.8–0.9 μM), exceeding the in vitro activity of the clinical standard camptothecin and producing significant tumor regression in human breast cancer xenograft models [179]. Mechanistic studies indicate that these oximes act as dual topoisomerase I and II (Topo I/II) poisons. The planar tetracyclic core intercalates into DNA, whereas the oxime side chain forms key hydrogen bonds within the Topo-DNA cleavage complex. These interactions induce pronounced S-phase arrest, DNA polyploidy, and caspase-3-mediated apoptosis, accompanied by strong γ-H2AX phosphorylation and PARP cleavage [179,180].

This dual-targeting paradigm extends to the indenoquinoxaline series. Compound **10a**, a dimethylaminopropoxyimino derivative, achieved IC_50_ values of 0.6–0.9 μM against highly aggressive MDA-MB-231, PC-3, and Huh-7 cells. Notably, it exhibited exceptional selectivity indices (SI = 36–49) over normal human fibroblasts and effectively suppressed tumor growth in a zebrafish xenograft model by concurrently inhibiting topoisomerases and downregulating the oncogenic Src/Akt phosphorylation pathways [181]. Similarly, 11-iminoindeno[1,2-*c*]quinoline oxime (compound **23a**) exhibited broad-spectrum activity against hepatoma, lung, and breast cancer lines (GI_50_ = 0.4–0.7 μM), inducing G2/M phase arrest through the upregulation of p53 and Bax, alongside robust caspase-8 activation [182]. IQ-1S effectively suppressed the growth of 3D spheroids and MCF7 cells in a 2D culture [183].

#### 3.4.2. Cytotoxicity of Metal Coordination Complexes

The high propensity of tetracyclic oximes to form chelate complexes was deliberately exploited to create transition metal compounds exhibiting significantly improved antitumor properties, particularly against drug-resistant phenotypes. Arene-ruthenium(II) “piano-stool” complexes incorporating indenoquinoxaline and tryptanthrin-6-oxime ligands exhibit potent micromolar cytotoxicity against human breast cancer cell lines, including cisplatin-resistant MCF-7CR variants (IC_50_ ≈ 9 μM) [184]. The anionic oximate coordination stabilizes the ruthenium center while preserving the planar aromatic core’s DNA-intercalating potential. In a complementary study, mono- and binuclear arene-Ru(II) *p*-cymene complexes bearing tryptanthrin-6-oxime demonstrated moderate cytotoxic effects against PANC-1 pancreatic cancer cells alongside notable catalytic transfer hydrogenation activity, illustrating the dual functional utility of the oximate coordination motif [185].

Platinum(II) and copper(II) complexes bearing tryptanthrin-derived ligands provide further support for this metallodrug approach. Among them, [Pt(BrTRP)(DMSO)Cl_2_] shows remarkable potency against bladder carcinoma T-24 cells (IC_50_ ≈ 0.2 μM), while displaying minimal toxicity toward normal hepatocytes [186]. These Pt(II) complexes induce S-phase arrest, reduce cyclin A/CDK2 expression, and act as potent telomerase inhibitors through selective targeting of the c-Myc promoter. At the same time, they promote mitochondrial dysfunction and apoptosis. Spectroscopic studies further show that platinum–tryptanthrin molecular complexes bind favorably to telomeric G-quadruplex DNA, consistent with a dual mechanism involving telomere destabilization and intercalative DNA damage [187]. Copper(II) complexes of tryptanthrin and 8-bromo-tryptanthrin show a related profile, with selective cytotoxicity against hepatocellular, bladder, and gastric cancer cell lines (IC_50_ = 4–9 μM). Their activity is mediated through DNA damage and interaction with the c-Myc promoter, illustrating how metal coordination can cooperate with the quinazoline scaffold to address classical chemoresistance mechanisms [188].

#### 3.4.3. Spiro-Heterocycle Conjugates and Multi-Targeted Hybrids

In addition to direct oximation and metal complexation, the exocyclic carbonyl position provides a valuable site for 1,3-dipolar cycloadditions, giving access to spiro-fused heterocycles with potent, selective, and multi-target antitumor activity. Spiro(indenoquinoxaline-pyrrolizidine] and spiro(tryptanthrin-pyrrolo[3,4-*a*]pyrrolizine] hybrids have shown significant cytotoxicity against erythroleukemia (K562), cervical carcinoma (HeLa), and colon carcinoma (CT26) cell lines. Their activity is associated with G2/M-phase accumulation, apoptosis, and marked cytoskeletal disruption, including actin filament depolymerization and loss of filopodia-like membrane protrusions [69,189].

Recent progress in MCR chemistry has considerably broadened this structural class. Saravana Mani et al. [190,191] synthesized spiro-indenoquinoxaline pyrrolizine and pyrrolothiazole hybrids containing a quinoline pharmacophore through an atom-economic cycloaddition strategy. Lead compounds displayed substantial cytotoxicity against MCF-7 and A-549 cells, comparable to that of doxorubicin, while also functioning as potent radical scavengers and inducing cell death through both apoptotic and necrotic pathways. In a related series, Zimnitskiy et al. [192] prepared spiro(indenoquinoxaline-pyrrolizidine]-N-arylpyrazole conjugates that showed strong, regioisomer-specific cytotoxicity against HeLa cells.

Spiro-architectures have been especially useful for directing activity toward specific kinase domains. A series of spiro(indolo[2,1-*b*]quinazoline-pyrano [2,3-*d*]pyrimidine] derivatives, prepared through a four-component reaction, showed notable efficacy. Lead compounds produced IC_50_ values below those of the clinical standard etoposide in multiple 2D and 3D cell culture models [193]. Further diversification of this scaffold generated spiro(chromene-indolo[2,1-*b*]quinazoline] and spiro(indolo[2,1-*b*]quinazoline-6,4′-pyrano [2,3-*c*]pyrazole]-5′-carboxamide derivatives. These compounds exhibited strong growth-inhibitory effects against pancreatic (Panc1), breast (MDA-MB-231), and colon (HT-29) cancer cells, often exceeding the activity of etoposide while retaining favorable safety profiles in normal human dermal fibroblasts [194,195].

Visible-light-promoted, catalyst-free synthetic methods have also afforded spiro indolo-quinazolinone-pyrrolo[3,4-*a*]pyrrolizine hybrids with IC_50_ values of 7–18 μM across several cancer cell lines. Molecular dynamics simulations supported their activity as potent inhibitors of both wild-type and mutant epidermal growth factor receptor (EGFR), with binding energies ranging from −10.2 to −11.5 kcal/mol [196]. Indenoquinoxaline-derived spiropyrazole heterocycles have likewise been identified as dual EGFR and VEGFR-2 kinase inhibitors. The lead compound showed an IC_50_ = 1.4 μM against HepG2 cells and induced pronounced G1-phase cell cycle arrest, together with a 36-fold increase in apoptosis [36].

Therapeutic versatility can be broadened further by introducing additional pharmacophores through oxime-derived intermediates. For example, tryptanthrin-1,2,3-triazole hybrids prepared by Cu(I)-catalyzed azide–alkyne cycloaddition of tryptanthrin-6-oxime *O*-propargyl ethers display an exclusive *E*-configuration and improved ADMET (absorption, distribution, metabolism, excretion, and toxicity) profiles. These hybrids also show strong binding affinity (−11.3 kcal/mol) and promising in vitro anticancer potency [197]. In addition, spirooxindole–indenoquinoxaline derivatives designed as tryptophanyl-tRNA synthetase (TrpRS) inhibitors exhibit dual antibacterial and anticancer activity, suppressing diffuse large B-cell lymphoma (DLBCL) proliferation at IC_50_ values of 2.9–4.8 μM [198]. Recent structural studies have also produced novel 11*H*-indeno[1,2-*b*]quinoxaline phosphonates and spiro-pyrazole derivatives with notable anticancer activity. These compounds showed promising in vitro cytotoxicity against human breast (MCF-7) and colon (HCT116) cancer cell lines. In parallel, comprehensive in silico ADME profiling and molecular docking confirmed favorable oral bioavailability and strong binding affinities toward relevant cancer-associated protein targets [199].

Spiro-heterocycles have continued to widen their therapeutic reach in oncology and neurology. Tryptanthrin-appended spiro-1-nitropyrrolizidine derivatives, obtained through a highly regiospecific three-component reaction, display clear in vitro anticancer activity against MCF-7 cells. In silico ADMET profiling of these adducts predicted high bioavailability along with strong BBB penetration scores (up to 0.8), underscoring their promise for CNS malignancies and for addressing the distribution limitations of the parent scaffolds [200].

#### 3.4.4. Mechanistic Convergence and Structure–Activity Considerations

The antitumor activity of tetracyclic ketoximes and their spiro-conjugates reflects several interrelated mechanisms: (i) DNA intercalation and Topo I/II poisoning, supported by the planar aromatic framework and further shaped by oxime-derived side chains; (ii) cell cycle disruption, mainly S- or G2/M-phase arrest, together with caspase-dependent apoptosis and mitochondrial dysfunction; (iii) telomerase inhibition through c-Myc promoter targeting or G-quadruplex stabilization; and (iv) multi-kinase inhibition made possible by strategic spiro-fusion or metal coordination.

**Figure 4:**
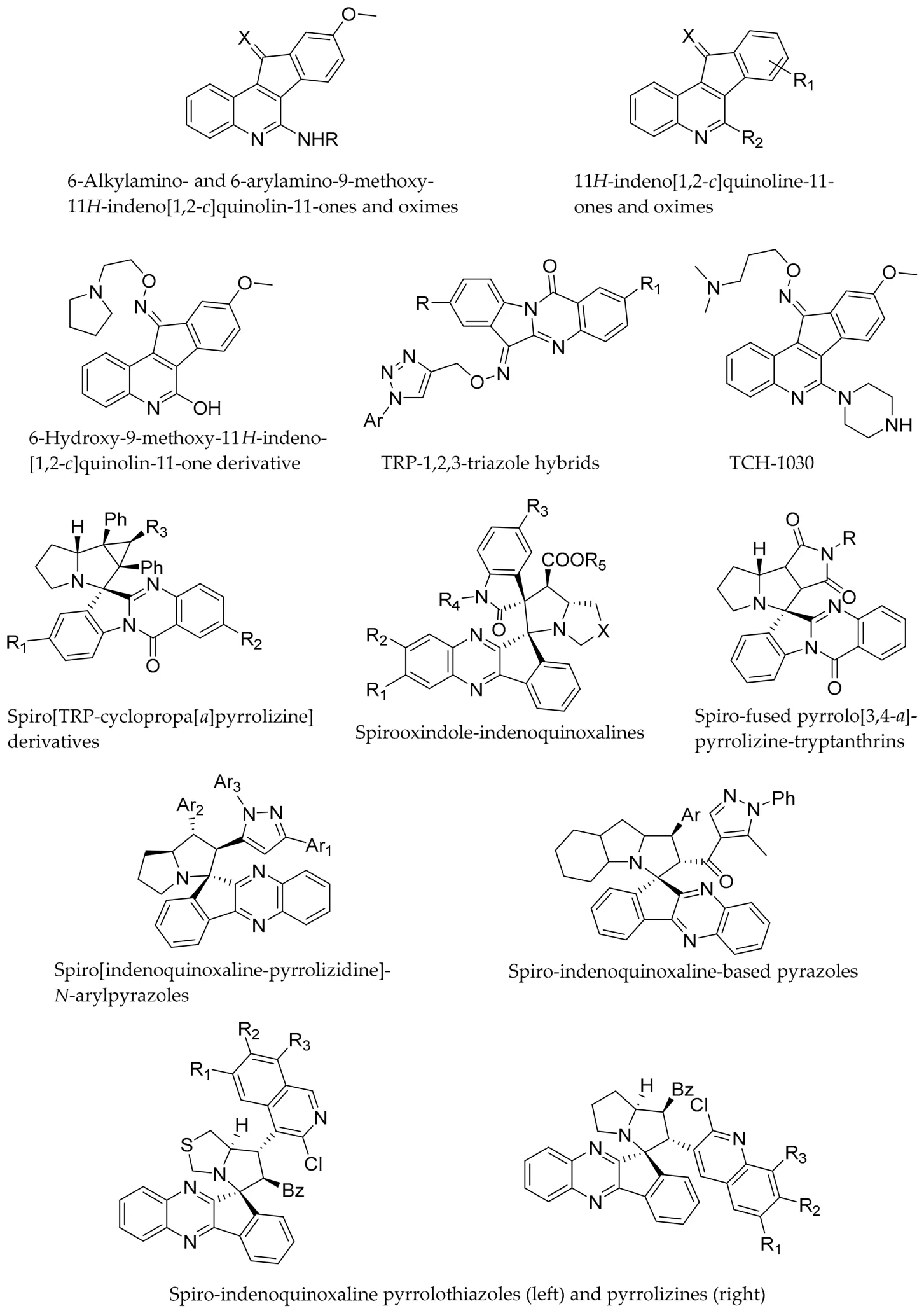
Chemical structures of selected compounds presented in Table 4.

The oxime group functions as a flexible electronic and steric modulator within these scaffolds. It increases hydrogen-bonding capacity in enzyme active sites, can improve aqueous solubility and thereby facilitate cellular uptake, and provides strong N,O- and N,N-chelating sites for metallodrug development. *O*-substituted oxime derivatives and their corresponding metal complexes are particularly notable because they often overcome cisplatin and camptothecin resistance, most likely through alternative DNA damage pathways and reduced recognition by cellular efflux pumps. Although the in vitro findings and early in vivo data are compelling, clinical translation will require further pharmacokinetic optimization, tumor-selective delivery strategies, and comprehensive toxicological evaluation. Table 4 summarizes the principal antitumor findings for these compounds; the chemical structures of selected compounds are shown in Figure 4.

### 3.5. Other Therapeutic Effects of Tryptanthrins and Related Derivatives

In addition to their established neuroprotective, cardioprotective, anti-inflammatory, and antineoplastic activities, tetracyclic ketoximes and closely related C=N-functionalized derivatives display a broad range of therapeutic effects. The indenoquinoxalinone and tryptanthrin scaffolds are structurally adaptable, especially when modified at the exocyclic carbonyl position, allowing them to engage diverse microbial, viral, parasitic, and metabolic pathways [15,28,202]. Introduction of the oxime pharmacophore changes the electronic distribution of the rigid tetracyclic core and adds chelating capabilities. This section reviews emerging evidence for the antimicrobial, antiviral, antiparasitic, antitubercular, and antidiabetic potential of these compounds, with emphasis on how oxime-derived pharmacophores and their diversified products may broaden clinical utility.

#### 3.5.1. Antimicrobial and Antifungal Activity

The planar, electron-deficient structures of indenoquinoxalinone and tryptanthrin oximes promote strong π–π stacking and intercalative interactions with microbial nucleic acids, a feature that contributes to their antibacterial and antifungal activity. Bandekar et al. [203] showed that tryptanthrin derivatives bearing a C-6 oxime group had the greatest bactericidal effect against *Escherichia coli*, reducing cell viability by factors ranging from 10^−2^ to <10^−6^ at concentrations of 0.2–40 μM. Linking-number assays further confirmed that these 6-oxime derivatives intercalate efficiently into bacterial DNA, thereby interfering with replication and transcription. High-affinity DNA binding is also characteristic of the indenoquinoxaline scaffold. Recent spectrophotometric and fluorescence quenching studies have demonstrated that indenoquinoxaline derivatives function as potent DNA intercalators, an activity that directly correlates with their antimicrobial and antiviral efficacy [204].

Similar DNA-targeting mechanisms have been observed in indenoquinoxaline-based spiro-heterocycles, which show enhanced membrane permeation and selective efficacy against Gram-positive pathogens due to optimized lipophilicity and reduced steric hindrance [205]. Furthermore, the chelating ability of these compounds has been exploited to create organometallic complexes with enhanced biocidal properties. For instance, the Re(I) carbonyl complexes of indenoquinoxalinone hydrazones have demonstrated superior Minimum Inhibitory Concentrations (MIC) against both Gram-positive (*S. aureus*, *B. subtilis*) and Gram-negative (*E. coli*, *P. aeruginosa*) bacteria compared to their free ligands, a phenomenon attributed to increased ROS and lipid peroxidation induction [206]. A related line of work examined a newly prepared *N*’-(11*H*-indeno[1,2-*b*]quinoxalin-11-ylidene)acetohydrazide ligand together with a series of its transition-metal complexes for biocidal activity. The Cd(II) and Zn(II) complexes stood out, showing markedly stronger broad-spectrum antibacterial and antifungal effects than the free ligand. Molecular docking reinforced these findings by indicating high binding affinities of the complexes for key microbial targets, among them *Staphylococcus aureus* DNA gyrase B and *Candida albicans* CYP51 [207].

In agricultural and phytopathological contexts, tryptanthrin analogues have been engineered to combat crop-threatening fungi and bacteria. Hao et al. [208] reported that specific tryptanthrin derivatives display broad-spectrum fungicidal activity, with selectivity against *Physalospora piricola* surpassing conventional agrochemicals. Further structural optimization through the introduction of fluorine and piperazine moieties yielded compound **6b**, which inhibited *Xanthomonas oryzae* pv. *oryzae* with an EC_50_ = 1.3 μg/mL, outperforming the commercial pesticides bismerthiazol and thiodiazole copper by over 25 fold [209]. Mechanistically, these derivatives induce ROS accumulation, trigger bacterial apoptosis, and disrupt biofilm formation, while proteomic analyses reveal targeted inhibition of type II and VI secretion systems. Complementary studies on indenoquinoxaline thiocarbohydrazones and Pd(II)-quinoxaline complexes further validate the scaffold’s adaptability, demonstrating robust activity against *S. aureus* and *C. albicans* alongside favorable radical-scavenging profiles [210,211]. A distinct antimicrobial targeting strategy has also emerged from recent work on spiro-indenoquinoxaline-pyrrolidines. Using virtual screening in combination with biophysical assays, these compounds were identified as promising small-molecule ligands for the CssA RNA thermometer in bacterial pathogens. By binding this regulatory RNA element, they interfere with translational control and suggest a new route toward RNA-targeted antibacterial therapy [212].

#### 3.5.2. Antiviral Potential

The rigid tetracyclic core, when functionalized with oxime or oxime-mimetic groups, has emerged as a highly promising scaffold for multi-target antiviral intervention. Historically, plants rich in these alkaloids, such as *I. tinctoria* and *S. cusia*, have been utilized in traditional medicine to treat viral respiratory infections [213,214]. Modern pharmacological screening has validated this ethnobotanical use. Tryptanthrin exhibits potent activity against human coronavirus NL63 (HCoV-NL63), reducing the cytopathic effects and progeny virus production with an IC_50_ = 1.5 μM and demonstrating strong virucidal activity at 60 nM [21]. Time-of-addition assays revealed that the compound interferes with both early and late replication stages, specifically blocking viral RNA genome synthesis and papain-like protease 2 (PLpro2) activity.

During the COVID-19 pandemic, the indoloquinazoline and indenoquinoxaline analogs garnered significant attention as a potential therapeutic agents against SARS-CoV-2 [215,216]. Jin et al. experimentally validated the predictions of a deep learning model and identified the compound IQ-1S, which exhibits pronounced synergistic antiviral activity against SARS-CoV-2 in vitro [217]. In silico molecular docking studies identified tryptanthrin as a potent dual antagonist capable of blocking the interaction between the SARS-CoV-2 spike glycoprotein and the host ACE2 receptor [218]. Furthermore, multi-phase computational screening of 310 natural metabolites highlighted tryptanthrin as a top candidate for inhibiting the SARS-CoV-2 papain-like protease (PLpro) [219]. Building on these computational predictions, Veselá et al. [220] designed tryptanthrin derivatives bearing thiosemicarbazone moieties, structurally analogous to oximes in their C=N coordination chemistry, that effectively chelate the Fe(II/III) ions implicated in COVID-19 pathogenesis. These compounds demonstrated high affinity for both the SARS-CoV-2 main protease (Mpro) and PLpro and significantly suppressed viral replication in Vero cell models.

Beyond coronaviruses, tryptanthrin derivatives show efficacy against influenza, respiratory syncytial virus (RSV), and plant viruses. Network-based pharmacological screening identified tryptanthrin as a key anti-influenza A virus (IAV) agent, exhibiting protective effects against wild-type H1N1 and H3N2 strains, as well as drug-resistant variants [221,222]. Quantitative assays demonstrated that tryptanthrin inhibits viral neuraminidase with an IC_50_ of 0.266 mg/mL [223]. In the context of RSV, related 4(3*H*)-quinazolone derivatives have been shown to regulate innate immune signaling by moderately inhibiting the retinoic acid-inducible gene-I (RIG-I) pathway, thereby suppressing excessive virus-induced interferon-β secretion and mitigating inflammatory injury [224,225]. In plant virology, tryptanthrin derivative (compound **B1**) binds directly to the viral genome-linked protein (VPg) of potato virus Y with a dissociation constant of 0.69 μM, pinpointing N121 as the critical residue for binding and highlighting VPg as a novel antiviral target [226].

#### 3.5.3. Antiparasitic and Antiprotozoal Effects

The redox-modulating capacity and DNA-intercalating potential of tryptanthrins translate into potent antiparasitic activity across multiple protozoan pathogens. Tryptanthrin derivatives have consistently demonstrated low nanomolar efficacy against *Toxoplasma gondii*. SAR studies emphasize the critical role of C-6 modifications; oxime, hydrazone, and alcohol analogues at this position significantly enhance parasite growth inhibition while maintaining low host cell cytotoxicity, establishing them as promising leads for toxoplasmosis therapy [22,227]. Similarly, tryptanthrin exhibits strong activity against *Leishmania* species, with IC_50_ = 8–11 μM against *L. infantum* and *L. amazonensis* promastigotes, respectively. Treatment induces mitochondrial membrane depolarization and triggers apoptosis-like death in intracellular amastigotes, with computational ADMET profiling predicting pharmacokinetic properties comparable to the clinical drug miltefosine [228]. Quantum chemical and 3D-QSAR analyses further suggest that electron transfer to the carbonyl/oxime region is a plausible mechanism of action, correlating strongly with antileishmanial potency [34]. In African trypanosomiasis, tryptanthrin analogues bearing electron-withdrawing groups at the C-8 position achieve an EC_50_ of 0.4 μM against *Trypanosoma brucei*, surpassing the efficacy of the parent tryptanthrin [229]. Complementary to these findings, Frank et al. [230] demonstrated that thioamide-substituted imidazoquinolinones, possessing a heterocyclic core structurally analogous to tryptanthrin, exhibit potent activity against *Trypanosoma cruzi* epimastigotes and amastigotes (IC_50_ = 1.5 and 1.7 μM, respectively). Parasite death is mediated by intracellular thiol pool-driven oxidative stress and apoptosis, extending the antiparasitic scope of this class of compounds to Chagas disease.

Antimalarial activity has also been investigated in considerable detail. Onambele et al. [23] reported that tryptanthrin derivatives maintain nanomolar antiplasmodial potency (IC_50_ = 30–100 nM) against both chloroquine-sensitive and chloroquine-resistant *Plasmodium falciparum* strains. The lead compound, NT1 (IC_50_ = 30 nM, selectivity index: 156), acts not only on asexual blood stages but also inhibits gametocyte maturation and reduces microgamete exflagellation by 20%, indicating meaningful transmission-blocking potential. Recent phytochemical screening of *Wrightia* species supports these observations, with isolated tryptanthrin showing IC_50_ values of 2.5 and 3.1 μM against *P. falciparum* 3D7 and Dd2 strains, respectively, together with negligible hemolytic toxicity [231]. Computational tunneling barrier analyses further show that Lowest Unoccupied Molecular Orbital (LUMO) lobe distribution is strongly correlated with antiplasmodial efficacy, offering a quantitative design parameter for next-generation oxime-based antimalarials [232]. Debbert et al. [233] also assessed a panel of quinoxaline derivatives against *Schistosoma mansoni* and identified compounds with IC_50_ ≤ 0.3 μM against adult worms. The in vivo reduction in worm burden, however, was only moderate (9–46%), emphasizing the need to optimize the pharmacokinetic properties of the quinoxaline scaffold for antischistosomal use.

#### 3.5.4. Antitubercular Activity

The rise of multidrug-resistant (MDR) and extensively drug-resistant *Mycobacterium tuberculosis* (Mtb) has made the search for new structural classes of antitubercular agents urgent [234,235]. The indoloquinazoline scaffold has long been known for its activity against Mtb [236], and oxime or related C=N derivatives now provide a means of targeting essential mycobacterial enzymes. Hwang et al. [237] carried out a systematic evaluation of tryptanthrin analogues, assessing both antitubercular potency and ADME properties. They identified C-11-deoxy and A-ring-saturated variants that showed improved solubility and oral bioavailability in rodents. Even so, efficacy in acute murine models remained limited, underscoring pharmacokinetic optimization as a key remaining obstacle.

Subsequent research has focused on enoyl-acyl carrier protein reductase (InhA), a validated Mtb target. Duca et al. [238] developed thiadiazoloquinazolinone analogues of tryptanthrin that achieve up to 100% inhibition of mycobacterial growth at a MIC of 6.5 μg/mL. Molecular docking confirmed stable binding within the InhA active site, facilitated by favorable non-covalent interactions. Similarly, phaitanthrin congeners (structurally related to tryptanthrin via C-6 keto modification) demonstrate promising activity against MDR Mtb strains, with in silico models predicting high affinity for InhA and favorable pharmacokinetic profiles [239]. Scaffold diversification via 1,3-dipolar cycloaddition has further enhanced antitubercular potency. Arumugam et al. [240] synthesized spiropyrrolidine-tethered indenoquinoxaline hybrids using an ionic liquid-accelerated multicomponent strategy. The resulting nitro-substituted analogue achieved a MIC = 1.6 μg/mL against Mtb H37Rv, comparable to the first-line drug ethambutol, underscoring how strategic oxime-derived cycloaddition pathways can generate highly active, stereochemically defined antitubercular leads. Recent target-oriented design efforts have also produced spiroindenoquinoxaline-derived pyrrolo[3,4-*c*]pyrroles with favorable binding interactions toward the Mtb enzyme decaprenylphosphoryl-β-D-ribofuranose oxidoreductase. These compounds showed potent in vitro antitubercular activity against the H37Rv strain [241].

#### 3.5.5. Antidiabetic and Metabolic Modulation

Tetracyclic ketoximes and their hydrazone/thiazole hybrids show clear promise for metabolic-disease management, particularly through inhibition of carbohydrate-digesting enzymes [242]. Hameed et al. [35] prepared a library of hydrazinyl thiazole-linked indenoquinoxaline hybrids and tested them against α-amylase and α-glucosidase. Several derivatives proved competitive inhibitors, with IC_50_ values of 0.3–77 μM for α-amylase and 1.1–92 μM for α-glucosidase, substantially better than the clinical standard acarbose (IC_50_ = 13.5 μM). Molecular docking showed that the oxime/hydrazone moiety and the extended π-system together position the ligands optimally within the enzyme active sites. The same compounds also exhibited strong 2,2-diphenyl-1-picrylhydrazyl (DPPH) radical-scavenging activity, indicating a dual capacity for glycemic control and mitigation of oxidative stress. Related work on Pd(II)-quinoxaline complexes demonstrated a dose-dependent α-amylase inhibition (IC_50_ = 326.5 μg/mL) that followed mixed-mode kinetics. Molecular-dynamics simulations and MM/GBSA free-energy calculations confirmed highly stable ligand–enzyme complexes dominated by van der Waals and lipophilic interactions [211]. Overall, these findings identify oxime-functionalized tetracyclic cages as attractive multi-target agents for type 2 diabetes and oxidative-stress-linked metabolic disorders.

#### 3.5.6. Concluding Remarks

Tetracyclic ketoximes offer therapeutic opportunities well beyond kinase inhibition or neuro- and cardiovascular protection. Their ability to intercalate nucleic acids, chelate metal ions, modulate redox balance, and occupy varied enzymatic active sites accounts for substantial antimicrobial, antiviral, antiparasitic, antitubercular, and antidiabetic effects. In vitro potency remains consistently high across these settings, yet translation into clinically useful agents will require focused attention to pharmacokinetic optimization, targeted delivery platforms, and thorough in vivo validation. Further work on oxime stereochemistry, scaffold hybridization, and metal coordination is expected to reveal additional mechanistic routes and broaden the clinical reach of this privileged scaffold class. Supplementary Table S1 assembles the principal antimicrobial, antiviral, antiparasitic, antitubercular, and antidiabetic findings for tryptanthrins and related structures, listing compound identity, target pathogen or enzyme, key activity data, proposed mechanisms, and supporting references.

### 3.6. Kinase Binding Affinity of the Tetracyclic Ketoximes

Tetracyclic ketoximes derive their broad therapeutic versatility from the ability to engage kinase catalytic domains with high affinity and, in several cases, marked isoform selectivity. The exocyclic oxime, embedded in a rigid planar indenoquinoxaline or tryptanthrin framework, serves as a privileged pharmacophore. This architecture fits snugly into ATP-binding pockets and forges key hydrogen bonds plus π–π stacking contacts with hinge-region residues and hydrophobic back pockets. Flexible acyclic inhibitors pay large entropic penalties on binding; the preorganized tetracyclic core largely avoids that cost. As a result, the oxime functions as a precise molecular anchor that governs both binding thermodynamics and kinetic residence time. The quinoxalinone nucleus itself is already regarded as a privileged platform in drug discovery [3], while planar heterocyclic systems are known to mimic adenine–kinase interactions through extensive hydrophobic contacts inside the adenine-binding region [243]. Independent reviews of kinase-inhibitor pharmacology have singled out the indenoquinoxalinone oxime chemotype as one of the most structurally distinctive and pharmacologically validated scaffolds described to date [85,244].

Designing isoform-selective JNK inhibitors has proven difficult, chiefly because the ATP-binding pockets of JNK1, JNK2, and JNK3 share very high sequence identity. Even so, systematic modification of the rigid, planar 11*H*-indeno[1,2-*b*]quinoxalin-11-one and tryptanthrin scaffolds has defined clear SAR that regulates both binding affinity and isoform selectivity. Within these tetracyclic systems, an exocyclic oxime or a related nitrogen-containing functionality is essential for JNK inhibition. The corresponding parent ketones, both indenoquinoxalinone and tryptanthrin, lack JNK binding affinity and do not inhibit LPS-induced inflammatory responses in cellular assays [19,20,86].

Substitution at the oxime group has a marked effect on both target engagement and cellular permeability. Unsubstituted oximes, *O*-acyl-oximes, and certain hydrazones, including semicarbazones, generally preserve high affinity for JNKs [19]. By contrast, bulky *O*-alkyl substituents, such as *O*-benzyl groups or simple hydrocarbon chains, eliminate activity, apparently because they generate steric clashes within the kinase binding cleft [20,176]. Small *O*-alkyl substituents on tryptanthrin oximes, such as *O*-allyl groups, are notable in that they confer moderate JNK3 selectivity, yet they show weak cellular anti-inflammatory activity, likely reflecting limited cellular uptake or microsomal instability [176]. *O*-Acyl derivatives, including acetoxyimino analogs, behave differently. They retain high binding affinity while substantially improving cellular efficacy, consistent with intracellular ester-bond cleavage and prodrug delivery of the active oxime pharmacophore [176]. Decorating the aromatic rings of the tetracyclic core provides a powerful mechanism for tuning JNK isoform selectivity. In the 11*H*-indeno[1,2-*b*]quinoxalin-11-one oxime series, the introduction of halogen atoms at specific positions enhances selectivity. For instance, a chlorine atom at the C-8 position (see Figure 1 for the corresponding atom numbering) increases the relative specificity toward JNK1 and JNK3 over JNK2 [20]. Similarly, simultaneous fluorination at the positions C-7 and C-8 yields compounds with high selectivity for JNK1/3 and no detectable binding to JNK2 [177]. However, steric bulk is poorly tolerated; the introduction of a *tert*-butyl group at C-7, or simultaneous multi-substitutions with bulky halogens and trifluoromethyl groups (e.g., Br or CF_3_ at C-8 combined with CH_3_, CF_3_, Cl, or Br at C-6), results in a complete loss of JNK2 affinity [177].

For tryptanthrin-6-oxime derivatives, single substitutions at the C-8 position (such as NO_2_, Br, OMe, or phenyl groups) act as a major driver for JNK3 selectivity, yielding selectivity indices greater than 7 fold over JNK1 and JNK2 [86]. Positional dependence is highly sensitive; shifting a methyl group from the C-7 position (which favors JNK3 selectivity) to the C-9 position converts the molecule into a potent, but non-selective, pan-JNK inhibitor. As observed with the indenoquinoxalines, excessive steric bulk, such as di-substitutions with methoxy groups or additional bromine atoms, abolishes binding affinity due to severe steric clashes with the kinase binding pocket [86].

Molecular docking results are broadly consistent with the experimental binding data, indicating that the planar tetracyclic core penetrates deeply into the narrow ATP-binding cleft of JNK isoforms and occupies a region comparable to that of the co-crystallized pan-JNK inhibitor SP600125 [19,20] (see Figure 3A). For active ketoximes, high-affinity binding appears to depend largely on well-defined H-bonding interactions. The oxime oxygen and nitrogen atoms serve as essential H-bond donors and acceptors, forming contacts with key hinge-region residues, including Lys93, Asn152, Gln155, Met149, and Asp207, with the interaction pattern varying according to the JNK isoform and ligand orientation [19,20,86]. Inactive analogs, by contrast, either do not establish these hydrogen bonds or adopt energetically unfavorable conformations. The loss of activity observed for bulky di-methoxy tryptanthrin derivatives, for instance, has been attributed to steric clashes with Met149, Asp150, and Ala151, which displace the molecule substantially from the preferred binding plane [86]. Finally, while these compounds are predominantly stable as *E*-isomers in aprotic solvents, they can undergo catalyzed *E*/*Z* isomerization in aqueous environments [80] or upon interaction with specific targets. Docking simulations indicate that the kinase binding site can selectively accommodate specific configurations. Depending on the peripheral substitutions, either the *E*-isomer (e.g., for *O*-acyl derivatives) or the *Z*-isomer (e.g., for unsubstituted oximes) provides the lowest-energy docking pose, suggesting that target-induced *E*/*Z* isomerization occurs to achieve optimal complementarity with the JNK binding pocket [176,177].

Despite the relatively low bioavailability of IQ-1/IQ-1S, the blood concentrations achieved upon oral administration at the indicated doses are comparable to the levels that elicit an anti-inflammatory effect and inhibit JNK [19,20]. The therapeutic efficacy of IQ-1 indicates that the concentration range employed ensures effective interaction with molecular targets, thereby significantly enhancing the compound’s translational potential. Similarly, for tryptanthrin-oxime, the K_d_ value for JNK1-3 is comparable to the concentrations at which inhibition of NF-κB/AP-1 transcriptional activity in THP-1Blue cells and IL-6 production in MonoMac-6 cells is observed [86].

While JNK inhibition remains extensively characterized, the oxime-functionalized tetracyclic cores and related C=N-modified derivatives have been successfully repurposed to target RTKs, particularly EGFR. The emergence of resistance mutations in EGFR necessitates the continuous development of novel inhibitors [245]. Molecular docking and enzymatic assays reveal that indenoquinoxaline-based spiro-pyrazole hybrids function as dual EGFR/VEGFR-2 inhibitors, achieving an IC_50_ of 1.35 µM against HepG2 cells through optimal occupation of the kinase hinge region [36]. The tryptanthrin core, when functionalized with aminothiazole or Schiff base moieties at the C-6 position, exhibits nanomolar binding affinities for EGFR, with the derivatives outperforming erlotinib in kinase assays and demonstrating highly favorable binding energies (−10.2 to −11.5 kcal/mol) in molecular dynamics simulations [246,247]. Furthermore, quinoxaline–piperazine and quinoxaline–oxazole conjugates have been identified as potent EGFR inhibitors, with specific derivatives demonstrating dual-inhibiting tendencies and IC_50_ values as low as 0.2 µM, significantly outperforming standard therapeutics like erlotinib [248,249,250]. Crucially, quinoxalinone scaffolds have shown exceptional promise against drug-resistant mutant strains. Suriya et al. [251] demonstrated that quinoxalinone-containing compounds effectively inhibit the advanced EGFR triple mutant (L858R/T790M/C797S), with IC_50_ values ranging from 3 to 10.5 nM, driven by critical interactions with hinge region residues (M793) and mutated residues (M790, S797). Similarly, Jiwacharoenchai et al. [252] identified quinoxalinone derivatives that strongly bind the EGFR tyrosine kinase domain via key residues, including L694, V702, L768, M769, and L820.

The affinity of indenoquinoxaline derivatives for RTKs also extends to vascular endothelial growth factor receptor 2 (VEGFR-2), a primary driver of tumor angiogenesis. Fluorinated indenoquinoxaline–thiazole conjugates demonstrate VEGFR-2 inhibition profiles rivaling sorafenib, attenuating p-VEGFR2 and p-AKT levels by over 77% in hepatocellular carcinoma models [253]. El-Adl et al. [254] successfully designed quinoxalin-2(1*H*)-one derivatives as analogs of lenvatinib and sorafenib, achieving superior in vitro cytotoxicity by replacing the central motifs of these clinical drugs with the versatile quinoxaline substructure. Other structural variations, such as [1,2,4]triazolo[4,3-*a*]quinoxalines [255] and (*Z*)-3-(2-(pyridin-4-yl)vinyl)quinoxalinones [256], have also exhibited potent anti-angiogenic effects via direct VEGFR-2 kinase inhibition at low nanomolar to micromolar concentrations.

The structural plasticity of the quinoxaline moiety allows it to engage a much broader spectrum of serine/threonine and non-RTKs. Quinoxaline-derived macrocycles and fused heterocycles have been engineered as potent PIM-1/2 inhibitors, achieving IC_50_ values of 74 nM and demonstrating subnanomolar cellular target engagement in multiple myeloma models [257,258,259]. In the realm of non-RTKs, pyrrolo[1,2-*a*]quinoxalin-4(5*H*)-one derivatives act as highly selective non-covalent Bruton’s tyrosine kinase (BTK) inhibitors (IC_50_ = 7.4 nM) with exceptional selectivity across a 468-kinase panel [260]. The scaffold’s adaptability is further evidenced by its application in targeting the transforming growth factor-β (TGF-β) receptor type-1 serine/threonine kinase (ALK5). Zhao et al. [261,262] developed 3-substituted-4-(quinoxalin-6-yl) pyrazoles and chiral rhodanine derivatives bearing a quinoxalinyl imidazole moiety that inhibited ALK5 phosphorylation, with IC_50_ values of 0.3 µM and 0.4 µM, respectively, displaying excellent selectivity over p38α MAPK.

Other notable kinase targets include DYRK1A/CLK1/CLK4, where benzo[b]indeno[1,2-d]thiophen-6-ones achieved IC_50_ values of 20–116 nM through ATP-competitive binding [263]; apoptosis signal-regulating kinase 1 (ASK1; IC_50_ = 30 nM) [264]; and FLT3 in acute myeloid leukemia, where quinoxaline–ureas bind the DFG-out inactive state [265,266]. The p38α MAPK and its downstream target MK2 are also effectively inhibited by quinoxaline derivatives containing triazole or hydrazone moieties [267,268] and azepine/oxazocine analogs [269,270,271]. Furthermore, tetracyclic architectures have shown profound efficacy against Aurora kinases [272], PDK1 [273], EphA3 [274], and PKMYT1 [275]. Macrocyclic quinoxalinone derivatives have also been engineered as pan-CDK inhibitors, achieving low nanomolar potency against CDK1, 2, 4, 5, 6, and 9 with excellent selectivity over non-CDK kinases [276].

The strong kinase-binding affinity of these compounds reflects more than steric complementarity; it also depends on electronic tuning and conformational preorganization. Peripheral electron-withdrawing substituents, including fluorine, nitro, and cyano groups, improve dipole–dipole interactions within the kinase active site and increase selectivity toward resistant mutants [253,277]. The oxime group can also participate in interaction networks within solvent-exposed regions of the ATP pocket; a feature associated with improved binding kinetics and longer target residence time [20,177]. This pattern of multi-target kinase engagement is consistent with the growing concept of dual-target-directed ligands, in which rationally designed polypharmacology exploits shared binding-pocket features among structurally related kinases [278]. Overall, the high kinase affinity of tetracyclic ketoximes arises from the combined effects of scaffold rigidity, precise oxime-mediated hydrogen bonding, and adjustable electronic properties. This recognition profile enables submicromolar to nanomolar engagement across multiple kinase families and supports the development of these compounds as versatile leads for targeted oncology, immunomodulation, and neuroprotection. Continued integration of structural biology, computational thermodynamics, and rational scaffold diversification should facilitate the design of next-generation oxime-based kinase inhibitors with improved selectivity, activity against resistant variants, and stronger translational potential.

### 3.7. Other Mechanisms of Therapeutic Effects of Tryptanthrins

Although high-affinity kinase inhibition and modulation of downstream stress-signaling pathways remain the best-characterized pharmacological actions of tryptanthrins, recent evidence points to a broader and more complex mechanistic profile. These compounds also influence innate immune responses, transcriptional programs, DNA topology, redox-dependent senescence, apoptosis, cellular differentiation, and microbial virulence. Such noncanonical mechanisms often act alongside established target engagement, producing pleiotropic therapeutic effects across inflammatory, oncological, and infectious disease contexts.

#### 3.7.1. Modulation of Innate Immunity and Inflammasome Complexes

Tryptanthrin derivatives act beyond the suppression of transcription factors by directly interfering with the assembly and activation of cytosolic inflammasome complexes. Ren et al. [27] showed that tryptanthrin binds stably to ASC-like protein through its pyrin domain. This binding disrupts ASC oligomerization and subsequent caspase-1 maturation. As a result, broad-spectrum inflammasome inhibition attenuates activation of NLRP3, NLRC4, and AIM2, leading to significant improvement in murine models of methionine- and choline-deficient diet-induced non-alcoholic steatohepatitis (NASH) and LPS-induced sepsis. Supporting in vivo studies further indicate that Isatidis folium extracts enriched in tryptanthrin suppress NLRP3 responses in macrophages and alleviate DSS-induced colitis by preserving mucosal integrity and tight junction protein expression [279].

Furthermore, Fan et al. [95] also reported that tryptanthrin promotes microglial polarization from the pro-inflammatory M1 phenotype toward the neuroprotective M2 phenotype through inactivation of the cGAS/STING/NF-κB axis. This immunomodulatory shift reduces secondary neuroinflammation, suppresses endoplasmic reticulum stress-related neuronal apoptosis, and improves functional recovery after spinal cord injury. In a complementary mechanism, Hesse-Macabata et al. [280] demonstrated that tryptanthrin increases the expression of the human β-defensins HBD2 and HBD3 in keratinocytes, even without fungal stimulation. At the same time, it attenuates *Trichophyton benhamiae*-induced pro-inflammatory cytokine release and prevents dermatophyte-mediated cellular damage. This combined ability to restrain excessive inflammation while enhancing antimicrobial peptide production supports the role of tryptanthrin as a balanced innate immune modulator capable of strengthening epithelial barrier defense.

#### 3.7.2. Transcriptional Reprogramming and Receptor Modulation

The tetracyclic cores and their derivatives also exert profound effects on cytokine signaling networks and ligand-activated transcription factors. In models of atopic dermatitis, tryptanthrin downregulates TSLP production in human mast cells by blocking the receptor-interacting protein 2 (RIP2)/caspase-1/NF-κB pathway. This subsequently reduces IL-4, IFN-γ, and TNF secretion while inhibiting mast cell proliferation via MDM2/p53 axis modulation [168,169]. The indoloquinazoline core also serves as a privileged substructure for AhR engagement. Mexia et al. [281] identified pityriazepin and structurally related indole metabolites as exceptionally potent AhR agonists, competitively binding to the receptor and directly stimulating AhR-dependent DNA binding. In oncology, tryptanthrin modulates critical transcriptional regulators. For instance, a semi-purified tryptanthrin-containing fraction DW-F5 from *Wrightia tinctoria* was shown to inhibit malignant melanoma by suppressing the WNT/β-catenin and Akt-NF-κB pathways, leading to the complete abolition of MITF-M, the master regulator of melanomagenesis [282]. Similarly, related quinazolin-2,4-diones have been shown to modulate STAT3 and FOXO3a signaling, driving apoptosis and necroptosis in hepatocellular carcinoma cells [283].

#### 3.7.3. DNA Topology Modulation and Topoisomerase Interference

The extended π conjugation of tetracyclic oximes facilitates direct physicochemical interactions with nucleic acids. Bandekar et al. [203] and Terryn III et al. [284] demonstrated that 6-oxime and 4-azatryptanthrin derivatives preferentially intercalate into GC-rich DNA regions (favoring GC over TA by approximately 2–4 kcal/mol), altering linking numbers and inducing topological stress that disrupts bacterial replication and transcription. In mammalian systems, tryptanthrin sulfonate exhibits moderate hypochromicity and significant viscosity increases upon calf thymus DNA binding, confirming an intercalative binding mode with an intrinsic binding constant of 1.1 × 10^4^ M^−1^ [285]. This intercalative capacity is a shared feature of related planar heterocycles, such as indolo[1,2-*b*]isoquinolin-5-ones [286].

Chen et al. [287] further established that aminoalkylamino-substituted indolo[2,1-*b*]quinazolin-6,12-dione derivatives specifically stabilize DNA triplex structures, with position-8 fluorine or methyl substitutions enhancing thermal stability by up to 6 °C through optimized side-chain orientation. These DNA-targeting properties synergize with topoisomerase inhibition; benzo[*b*]tryptanthrin functions as a non-intercalative, ATP-competitive dual Topo I/II catalytic inhibitor, effectively reversing adriamycin resistance by downregulating multidrug resistance protein 1 (MDR1) expression in breast cancer cells [288,289]. The introduction of transition metals further amplifies this genotoxicity. For instance, Zn(II) complexes bearing tryptanthrin and curcumin ligands accumulate in the cell nuclei, inducing profound DNA damage and cell cycle arrest in cisplatin-resistant A549/DDP lung cancer cells [290]. Similarly, the novel tryptanthrin derivative D6 induces severe DNA damage in non-small cell lung cancer cells, as evidenced by an increased p53/MDM2 ratio and the concentration-dependent accumulation of micronuclei [291].

#### 3.7.4. Redox Homeostasis, Apoptosis, and Cellular Differentiation

The tryptanthrin derivatives exhibit context-dependent redox modulation and apoptosis induction that can be harnessed for targeted cytotoxicity. Zhang et al. [30] identified tryptanthrin as a direct binder and inhibitor of GSTP1, a critical regulator of cellular redox homeostasis. GSTP1 inhibition triggers ROS accumulation, DNA damage response, and the NF-κB-mediated senescence-associated secretory phenotype (SASP) in liver cancer cells. This senescence induction exposes tumor vulnerabilities, significantly sensitizing cells to Bcl-2 inhibitors like ABT263 and establishing a novel senolytic-combinatorial strategy.

The induction of apoptosis by these tetracyclic compounds is frequently mediated by the intrinsic mitochondrial pathway. In human chronic myeloid leukemia (K562) cells, tryptanthrin induces pyknosis, chromatin margination, and a dramatic decrease in mitochondrial membrane potential, leading to the release of cytochrome c, upregulation of BAX, downregulation of Bcl-2, and activation of caspase-3 [292]. Similar apoptotic mechanisms, including cell cycle arrest at the G2/M and G1/S phases, have been validated for thiazole-indenoquinoxaline hybrids [293]. Interestingly, tryptanthrin also drives cellular differentiation. In human monocytic (U-937) and promyelocytic (HL-60) leukemia cells, tryptanthrin enhances the expression of monocyte/macrophage CD markers and triggers Fas-mediated apoptosis [294]. In neuroblastoma LA-N-1 cells, it induces G0/G1 cell cycle arrest, promotes neuronal differentiation (as evidenced by enhanced acetylcholine esterase activity), and significantly downregulates the N-Myc oncogene [295].

Furthermore, structural modifications can overcome classical chemoresistance. Dihydroxyquingdainone, an indigoid derived from tryptanthrin, induces caspase-3-dependent apoptosis and effectively overcomes p-glycoprotein-mediated MDR in leukemia cells [296]. Concurrently, nano-encapsulated tryptanthrin derivatives have been shown to induce immunogenic cell death while potently inhibiting indoleamine 2,3-dioxygenase, thereby remodeling the tumor immune microenvironment and enhancing antitumor efficacy in melanoma models [297]. In a mechanistically distinct paradigm, Chang et al. [298] demonstrated that the tryptanthrin-derived indoloquinazoline CIQ paradoxically activates, rather than inhibits, ERK, JNK, and p38 MAPK pathways in breast cancer cells, driving caspase-dependent apoptosis and suppression of invasion via MMP-2 downregulation. This illustrates how subtle structural modifications within the same tetracyclic scaffold can elicit diametrically opposite signaling outcomes to achieve convergent antitumor effects.

#### 3.7.5. Microbial Virulence Attenuation and Quorum Sensing Disruption

In antimicrobial applications, tryptanthrin extends beyond direct bactericidal activity to target pathogen communication and virulence regulation. Tryptanthrin inhibits biofilm formation and motility in *Vibrio splendidus* by competitively binding to the sigma-54-dependent transcriptional regulator LuxO_1_, a central quorum-sensing node, thereby reducing hemolytic activity and flagellar gene expression [299]. Similarly, in toxigenic *Vibrio cholerae*, tryptanthrin suppresses biofilm maturation at sub-MIC concentrations by targeting LuxO, sensitizing pathogens to conventional quinolone antibiotics and circumventing MDR [300].

In phytopathogenic contexts, fluorinated and piperazine-substituted tryptanthrin analogs disrupt type II and VI secretion systems (T2SS/T6SS), impairing membrane transport, inducing bacterial apoptosis, and demonstrating superior in vivo therapeutic efficacy against citrus canker compared to commercial pesticides [209,301]. Furthermore, 7-aliphatic amine tryptanthrin derivatives exhibit potent activity against *Xanthomonas oryzae* pv. *oryzae* by increasing intracellular ROS accumulation, causing bacterial cell apoptosis, and disrupting biofilm integrity [302]. The anti-virulence repertoire extends to *Campylobacter jejuni*, where tryptanthrin exerts a direct bactericidal mechanism with a narrow antimicrobial spectrum and minimal potential for resistance development, significantly reducing cecal colonization in chick infection models [303]. In the fungal domain, tryptanthrin derivative **5b** exhibits remarkable synergy with fluconazole against drug-resistant *C. albicans*, mechanistically attributed to the suppression of hyphal growth and biofilm formation rather than direct fungicidal activity [304]. Similarly, Lin et al. [305] established that tryptanthrin induces cell cycle arrest at G1/S and synergizes with calcineurin inhibitors against *Cryptococcus neoformans*, engaging multiple signaling pathways, including the protein kinase A cascade and the calcium transporter Pmc1. These anti-virulence mechanisms position tryptanthrin derivatives as promising adjuvants that disarm pathogens rather than merely exerting selective pressure for resistance.

#### 3.7.6. Emerging Targets and Resistance Mechanisms

Compounds built on these tetracyclic cores also engage biosynthetic and proteolytic targets beyond topoisomerases. Spirooxindole–indenoquinoxaline hybrids act as potent bacterial TrpRS inhibitors and display dual antibacterial plus DLBCL antiproliferative activity [198]. Tryptanthrin analogs, for their part, produce unprecedented inhibition of the human 20S constitutive proteasome, pointing to a possible role in disrupting protein homeostasis within rapidly proliferating cells [306].

Separate from these cytostatic actions, tryptanthrin accelerates excisional wound healing in mice by altering the expression kinetics of vascular endothelial growth factor (VEGF) and MMP-9, yielding closure rates as high as 94% without residual scarring [307]. Such regenerative capacity implies that tryptanthrin derivatives may prove useful in skin repair and the management of postoperative wounds. In the mycobacterial setting, InhA was first advanced as a primary target. However, recent genomic work shows that resistance in *Mycobacterium smegmatis* arises mainly from mutations in the MmpS5-MmpL5 efflux system and EmbR/TetR transcriptional regulators [29]. Preserving antitubercular efficacy will, therefore, require either structural optimization or co-administration of efflux-pump inhibitors.

Taken together, the engagement of inflammasome adaptors, redox-driven senescence, nucleic acid intercalation, quorum-sensing disruption, and inhibition of essential biosynthetic enzymes places these molecules within a densely interconnected mechanistic network. Structural refinement aimed at greater target selectivity, rational combinations that exploit senolytic or efflux-blocking strategies, and validation of the alternative pathways in clinically relevant disease models will be needed to convert the translational promise of this privileged chemical class into practical therapeutics.

## 4. Limitations and Perspectives

Tetracyclic ketoximes display a compelling and multifaceted pharmacological profile, yet their advance from promising preclinical leads to clinically viable therapeutics is still limited by pharmacokinetic, toxicological, and formulation-related constraints. Clearing these translational barriers will require a multidisciplinary strategy that combines rational compound optimization, advanced drug-delivery platforms, and rigorous in vivo modeling.

Convincing preclinical data notwithstanding, clinical translation of this chemical class still hinges on solving key pharmacokinetic, stereochemical, and formulation challenges. Only recently has the thermodynamic preference for the *E*-configuration of the oxime bond been firmly established; that geometry governs how the pharmacophore orients inside enzyme active sites [44]. Parallel efforts now focus on water-soluble salt forms (for example, the tryptanthrin derivative mostotrin), prodrug design, and incorporation of oxime derivatives into advanced nanoparticle delivery systems—all active frontiers in current research [308].

The principal limitation of tetracyclic ketoximes and their parent ketones is poor oral bioavailability combined with rapid systemic clearance, a pattern that often separates strong in vitro activity from more modest in vivo efficacy. Comprehensive pharmacokinetic profiling of IQ-1 in rats, for example, showed an absolute oral bioavailability below 1.5%. Detectable pharmacological effects required relatively high doses of 50–100 mg/kg, which produced maximum plasma concentrations C_max_ of only 24.7 to 37.6 ng/mL [110]. After intravenous administration, IQ-1 follows a two-compartment pharmacokinetic profile marked by extensive tissue distribution, with a steady-state volume of distribution (Vss) exceeding total body water by 3.6 to 5.6 fold, and very high clearance rates corresponding to 88–94% of hepatic blood flow [109]. These findings point to substantial first-pass metabolism and rapid hepatic elimination, both of which limit the ability to maintain sustained therapeutic plasma concentrations.

Similar translational gaps have been observed with related scaffolds. Early tryptanthrin analogs showed potent in vitro antitubercular activity and improved aqueous solubility, yet failed to suppress Mtb replication in acute murine models even at doses up to 400 mg/kg/day [237]. Likewise, quinoxaline analogs tested against *S. mansoni* displayed excellent in vitro potency (IC_50_ ≤ 0.3 μM) but produced only moderate reductions in worm burden in vivo (9.3–46.3%), again underscoring the need for pharmacokinetic optimization [233]. The same rigid, planar, highly lipophilic architecture that supports high-affinity target binding also contributes to poor aqueous solubility and limited gastrointestinal absorption. Structural or formulation-based interventions are, therefore, required to improve systemic exposure. Several technologies are available to increase the water solubility and bioavailability of lipophilic compounds, including micronization, crystal engineering, self-microemulsifying systems, liposomes and their derivatives, phospholipid nanoparticles, inclusion complexes through clathrate formation, and chemical modification. Even so, improving the bioavailability of tetracyclic ketoximes remains an unresolved challenge.

This section classifies existing problem-solving strategies into several practical areas: (i) rational molecular design, (ii) refinement of “green” synthesis methods, (iii) innovative drug delivery systems, (iv) high-throughput screening of multi-target ligands using artificial intelligence, and (v) pathways for clinical translation. It also evaluates experimental data supporting the efficacy of each approach, with an emphasis on what is realistically achievable in the near term.

### 4.1. Rational Molecular Design

The first actionable design principle is rigorous isomer control. Single-crystal X-ray diffraction, 1D/2D NMR spectroscopy, and DFT calculations have unambiguously established that IQ-1 and tryptanthrin-6-oxime adopt the *E*-configuration in both the solid state and solution, stabilized by intermolecular rather than the historically assumed intramolecular hydrogen bonds [44]. Because oxime geometry dictates the spatial projection of the N,O-pharmacophore into kinase catalytic clefts, stereochemical verification should be treated as a mandatory quality-control step before the biological profiling of any new derivative. Intriguingly, isomer control extends to metal-coordinated oximates. DFT modeling of the lithium salt IQ-1L indicated that Li^+^ stabilizes the six-membered oximate chelate ring better than Na^+^, and molecular docking predicted that the *Z*-configured oximate should bind JNK1 and JNK3 better than (*E*)-IQ-1. Experimentally, IQ-1L indeed exhibited higher JNK1–3 binding affinity than the sodium salt IQ-1S and achieved comparable neuroprotection at half the dose (12 vs. 25 mg/kg) [90]. Counterion selection, therefore, represents a legitimate, underexploited isomer-control variable.

A coherent, experimentally validated SAR map now exists to guide peripheral substitution. Electron-withdrawing groups at the C-8 position of the tryptanthrin core confer the greatest antitrypanosomal potency [229] and strengthen tryptophan 2,3-dioxygenase inhibition beyond the reference inhibitor LM10, with docking indicating coordination of the ring oxygen to the heme iron [42]. C-8 substitution also delivers kinase isoform selectivity. The 8-methoxy and 8-phenyl tryptanthrin-6-oximes display high selectivity for JNK3 over JNK1/JNK2, whereas the 9-methyl analogue is a pan-JNK inhibitor [86]. Similarly, 8-nitrotryptanthrin inhibits colorectal cancer cells markedly more potently than the parent [309]. At C-9, aliphatic amine substituents yield phytopathogenic antibacterial leads against *Xanthomonas* and *Pseudomonas* spp. [310]. At C-7, a (2-(dimethylamino)ethyl)amino side chain confers both high water solubility and Topo II inhibition exceeding etoposide [311]. Ring-level rules have also emerged. Halogenation of the D-ring enhances antitumor activity, whereas the same substitution on the A-ring reduces it [312]; and electron-withdrawing substituents on appended phenyl rings are crucial for antibiofilm activity against *Vibrio parahaemolyticus* [313].

Hydrazone chemistry at C-6 has yielded EGFR inhibitors (KBR-7, KBR-8) that outperform erlotinib in kinase assays [247], and benzenesulfonamide substituents redirect the scaffold toward p38α [149] or toward dual cholinesterase/multi-target anti-Alzheimer profiles [24]. For antiparasitic indications, C-6 analogues emerged as the most promising anti-*Toxoplasma* leads for therapy [227]. Two cautionary constraints should govern all such modifications. First, solubility-driven simplification of the tetracyclic core (C-11-deoxy or A-ring saturation) abolished antitubercular efficacy despite improved ADME parameters day [237], indicating that the planar pharmacophore must be preserved. Second, scaffold-level changes such as benzo-annulation predictably retune Topo I/II inhibitory balance [32] and should be used deliberately rather than empirically.

### 4.2. Green Synthetic Upgrading

Actionable green upgrading can be judged by three practical criteria: scalability, ease of purification, and the availability of quantitative sustainability metrics. Visible-light photocatalysis performs particularly well with respect to scalability. Jing et al. [55] demonstrated a metal- and photocatalyst-free intramolecular C–N cross-coupling on a Gram scale and under solar irradiation, while the Rose Bengal-mediated de novo photocatalytic synthesis from simple indoles has been explicitly framed as eco-friendly, cost-effective, and scalable [314]. Related photochemical methods broaden this platform further, enabling tryptanthrin construction through aerobic single-electron transfer in the absence of any photocatalyst [58], radical–radical cross-coupling without external oxidants [57], and organic-dye-mediated energy transfer under household-bulb irradiation [56,59]. Microwave-assisted solvent-free condensations [52,53,54], together with sonochemical and biocatalytic protocols [60,63], similarly provide mild reaction conditions and substantially reduce waste generation.

A persistent limitation in this literature is that quantitative green metrics are reported only sporadically; they should instead become routine. Borah et al. [61], for instance, provided an exemplary case in their ultrasound-assisted organocatalytic domino synthesis of spiro-indenoquinoxalines, where atom economy, reaction mass efficiency, E-factor, process mass intensity, and carbon efficiency were all explicitly calculated. Bokam et al. [62] addressed the purification burden from a different angle. Their four-component ultrasonic protocol followed group-assisted purification chemistry and avoided chromatographic purification altogether. Several aqueous or alternative-media methods also point toward genuinely low-E-factor production. These include β-cyclodextrin catalysis in water at room temperature [66], use of a recyclable water-soluble Co(III)-porphyrin catalyst [67], catalyst-free multicomponent reactions in water that furnish 73–98% yields without column chromatography [68], and a urea/ZnCl_2_ deep eutectic solvent that can be reused over three cycles [65]. Other approaches further strengthen the case for scalable, lower-impact synthesis. Electrosynthesis from isatin with atmospheric oxygen as the terminal oxidant shows that electron-transfer-driven assembly can be achieved with low energy input [64], while phosgene-free preparation of *N*-carboxyanhydrides directly from amino acids and CO_2_ provides one-pot access to tryptanthrin with a qualitatively greener profile than conventional routes [315]. In the same practical vein, a two-step, one-pot NaOCl-based process has been proposed as industrially applicable because of its low cost and operational simplicity [316].

The most conspicuous unresolved issue is the oximation step itself, which is still most often carried out with hydroxylamine under conventional reaction conditions. Several newer cascade oximation strategies suggest a clear path forward. These include one-pot oximation/C–H activation/alkyne annulation, notable for its atom, step, and redox economy [49]; cascade oxime formation–cyclization–dipolar cycloaddition sequences [48]; and rongalite/iodine-mediated C(sp^3^)–H oximation using copper nitrate as a mild [NO] source, a method already shown to tolerate fused-ring skeletons [43]. These approaches are, therefore, the most logical candidates for adaptation to tetracyclic ketone substrates. As a practical objective, future work should aim to develop telescoped one-pot processes in which a green core-assembly step is coupled directly to catalytic oximation, with the overall sustainability assessed using the quantitative metrics framework established by Borah et al. [61].

### 4.3. Formulation Innovations and Resistance Mechanisms

Given the bioavailability ceiling documented above, formulation-level interventions arguably offer the fastest route to clinically meaningful exposure. Salt derivatives already provide proof of concept. Mostotrin is at least five orders of magnitude more water-soluble than tryptanthrin, shows 5–10-fold higher cytotoxicity against HCT-116, MCF-7, and K-562 tumor lines, a five-fold higher LD_50_ (375 versus 75 mg/kg), retained DNA-binding capability, and improved survival in combination with doxorubicin in vivo [308]. Sulfonation to tryptanthrin sulfonate likewise increased water solubility while preserving intercalative DNA binding [285]. The C-7 cationic side-chain strategy of Catanzaro et al. [311] and the explicit solubility-driven design of dual indoleamine 2,3-dioxygenase 1/tryptophan 2,3-dioxygenase inhibitors [317] confirm that water solubility and target potency can be co-optimized rather than traded off.

Nanoparticulate and depot systems provide spatially controlled exposure where systemic delivery is unnecessary or undesirable. Electrosprayed PLGA microparticles incorporating IQ-1 release the inhibitor in a prolonged, non-Fickian two-step profile and suppress neutrophil activation and IL-6 secretion [173]; electrospun PCL scaffolds doped with IQ-1 release via Fickian diffusion and inhibit neutrophil ROS production, Ca^2+^ mobilization, and monocytic NF-κB/AP-1 activation without cytotoxicity [174]. These platforms are immediately actionable for localized indications (implant coatings, intra-articular or dermal depots) and establish design rules (particle size, release kinetics, polymer–drug hydrophobic interactions) transferable to nanoparticle formulations intended for systemic or CNS delivery. Finally, delivery strategies should be resistance-aware. Because tryptanthrin resistance in mycobacteria arises from MmpS5-MmpL5 efflux rather than target mutation [29], co-formulation or co-administration approaches that circumvent efflux should be evaluated alongside the carrier itself.

The clinical utility of indenquinoxaline and tryptanthrin derivatives is further complicated by pathogen- and tumor-mediated resistance mechanisms. In mycobacterial models, spontaneous resistance to tryptanthrin is primarily driven by the upregulation of the MmpS5-MmpL5 efflux system and mutations in transcriptional regulators (EmbR, TetR), rather than target-site alterations [29]. This underscores the necessity of co-administering efflux pump inhibitors or engineering sterically hindered oxime derivatives that evade active transport.

Interestingly, while the scaffold is susceptible to certain efflux mechanisms, it also possesses a remarkable capacity to overcome classical MDR in oncology. Benzo[b]tryptanthrin has been shown to successfully reverse adriamycin resistance in breast cancer cells by downregulating the expression of MDR1 [288,289]. Similarly, dihydroxyquingdainone, an indigoid derived from tryptanthrin, effectively overcomes p-glycoprotein-mediated MDR in leukemia cells [296]. Where compensatory survival pathways limit efficacy, rational combination therapies offer a solution. Recent evidence indicates that tryptanthrin-induced cellular senescence via GSTP1 inhibition and ROS accumulation can be leveraged synergistically with senolytic agents (e.g., ABT263) to induce apoptosis in resistant liver cancer cells [30].

Recent advances in nanotechnology have begun to address two persistent limitations of tryptanthrin and its derivatives: poor solubility and restricted tissue penetration. In dermatological applications, ethosome-mediated delivery of tryptanthrin has been developed as an effective anti-psoriatic nanotherapy. These deformable phospholipid vesicles enhance topical drug absorption, help restore lipid homeostasis, and improve cellular permeability without producing systemic toxicity [318]. A related topical strategy uses a double-sided Janus-structured nanofibrous patch with asymmetric wettability to achieve controlled, directional delivery of tryptanthrin and indirubin; its anti-psoriatic efficacy is comparable to that of first-line clinical ointments [319]. Nanotechnology has also proved useful in agricultural applications. Encapsulation of highly active tryptanthrin derivatives in pH-responsive ZIF-8 metal–organic framework nanoparticles reduces their phytotoxicity while enabling targeted release under weakly acidic conditions. These nanoformulations provide superior protective and curative activity against plant bacterial canker and wilt diseases, while limiting toxicity to host plants [320,321].

### 4.4. High-Throughput and AI-Aided Screening

The fourth area concerns discovery throughput and the rational development of polypharmacology. This scaffold class is unusually well-positioned for such work, since it already has a substantial computational foundation that can be extended with contemporary AI/ML workflows. In an early example, 3D QSAR pharmacophore modeling using CATALYST, together with AM1 quantum-chemical and cyclic–voltammetric descriptors, generated a predictive model of antileishmanial activity across 27 tryptanthrin analogues and implicated electron transfer to the carbonyl region as a key determinant of activity [34]. Subsequent pharmacophore-based virtual screening of compound libraries identified antimalarial candidates from structurally distinct chemotypes and supported the custom design and synthesis of potent analogues [322]. Target deconvolution has also benefited from computational methods. Reverse docking correctly predicted p38α as the relevant molecular target, a finding later confirmed by cellular thermal shift assay [149]. A complementary example comes from the experimental–computational interface, where comprehensive two-dimensional K562/cell-membrane chromatography combined with in silico target identification established tryptanthrin as the anti-leukemia component of Indigo naturalis. This workflow illustrates how affinity-based wet-laboratory screening can be integrated with computational target fishing.

Current derivative-discovery programs increasingly incorporate in silico ADMET prediction together with docking and molecular-dynamics tiers at an early stage. For example, PreADMET analysis likewise indicated that the pharmacokinetic and toxicological profile of tryptanthrin is comparable to that of miltefosine [228]. Similar computational workflows, combining SwissADME, molecular docking, and molecular dynamics simulations, have been used to prioritize several classes of derivatives, including Re(I) organometallics [323], aminothiazole hybrids with binding energies superior to gefitinib, erlotinib, and lapatinib [246], and Schiff-base EGFR inhibitors [247].

The feasibility of MTDL design is also well-supported within this chemical family. Benzenesulfonamide tryptanthrins, for instance, behave as mixed reversible dual AChE/BuChE inhibitors and combine anti-Aβ-aggregation, anti-neuroinflammatory, metal-chelating, BBB-permeable, and in vivo cognition-restoring properties [24]. In a related series, the selective AChE inhibitor (compound **n1**), with a selectivity index of 517 over BuChE, reversed scopolamine-induced memory impairment in mice [25]. Additional examples, including dual indoleamine 2,3-dioxygenase 1/tryptophan 2,3-dioxygenase inhibitors [317], dual antibacterial/antilymphoma TrpRS inhibitors [198], and dual EGFR/VEGFR-2 spiro-heterocycles designed with Molecular Electron Density Theory (MEDT) [36], further show that the planar oxime core can accommodate the pharmacophore merging, linking, and fusing strategies described in the MTDL hybridization framework [235].

These observations point to a practical discovery agenda. First, the existing SAR data should be organized into curated training sets suitable for machine-learning models of potency and selectivity. Those models can then be incorporated into generative or virtual-screening workflows linked to the docking/MD/ADMET cascade already demonstrated in the studies discussed above. Computational triage should also be paired with high-throughput experimental assays, so that the design–make–test cycle becomes iterative rather than sequential. Finally, candidate selection should rely on multi-objective optimization, with potency, isoform selectivity, aqueous solubility, and predicted oral exposure evaluated together. Such an approach would address, at the design stage, the pharmacokinetic failure modes documented by Frelikh et al. [110] and Hwang et al. [237], rather than attempting to correct them only after lead compounds have already been identified.

### 4.5. Strategic Pathways for Clinical Translation

Future work on tetracyclic ketoximes should center on three closely related priorities. First, computational and artificial intelligence-guided molecular design should be coupled with high-throughput ADME screening. Comprehensive 2D cell membrane chromatography paired with in silico target identification, together with multi-phase computational screening [219], will be important for predicting and optimizing solubility, metabolic stability, and target selectivity before synthesis. Second, continued development of MTDLs containing tryptanthrin cores is warranted, as these compounds have already shown improved BBB permeability and in vitro metabolic stability, providing a rational framework for CNS-focused design [24,25]. Third, rigorous translational studies in disease-relevant animal models are needed to address the efficacy gaps that often emerge in early preclinical development. The recent finding that tryptanthrin-6-oxime provides prolonged protection of BBB integrity after cerebral infarction, outperforming the parent ketone in sustained pharmacodynamic modulation, illustrates the therapeutic value of strategic oxime functionalization [324]. Although recent computational models suggest that some newly described spiro-1-nitropyrrolizidine derivatives of tryptanthrin have intrinsically high BBB penetration scores [200], efficient delivery of more polar derivatives or water-soluble salts will still depend on advanced carrier systems. Several recent reports have shown that nanotechnology-based approaches can improve the BBB permeability of oxime-containing heterocycles [325,326]. Together, these findings provide strong proof of concept for the eventual development of BBB-penetrating nanocomplexes built around tetracyclic ketoximes. Integrating such nanoformulations with newly identified potent spiro-hybrids and water-soluble salts should substantially accelerate the development of CNS-targeted therapies. As structural and mechanistic knowledge of this chemical class continues to expand, progress will likely depend on the integration of precision pharmacology, advanced biomaterials, and systems-level toxicology to support the development of tetracyclic ketoximes as next-generation therapeutics.

## 5. Conclusions

The systematic exploration of 11*H*-indeno[1,2-*b*]quinoxalin-11-ones, tryptanthrins, and their oximes has firmly established this chemical class as a highly privileged and versatile one in modern medicinal chemistry. The convergence of sustainable synthetic methodologies, definitive stereochemical characterization, and comprehensive pharmacological profiling underscores their exceptional potential for drug discovery. Recent synthetic advances have successfully transitioned the assembly of these rigid tetracyclic cores toward “green chemistry” paradigms. The implementation of microwave-assisted, sonochemical, and multicomponent strategies has not only streamlined synthetic access but also significantly improved quantitative green metrics, such as atom economy and process mass intensity, while preserving the exocyclic carbonyl as a highly tunable reactive handle [14,61,63]. Crucially, the resolution of historical structural ambiguities through integrated crystallographic, spectroscopic, and DFT approaches has provided a reliable stereochemical foundation for rational drug design. Thermodynamically stable *E*-configuration may favor a target binding, optimally projecting the N,O-pharmacophore into binding pockets [37,44]. However, for certain unsubstituted derivatives, target-induced isomerization allows access to the *Z*-configuration when required to maximize receptor engagement.

Pharmacologically, tetracyclic ketoximes exhibit a remarkable multi-target profile anchored by the high-affinity inhibition of JNKs. The oxime moiety enables precise hydrogen-bonding networks within the ATP-binding cleft, yielding submicromolar to nanomolar dissociation constants and the potent suppression of downstream NF-κB/AP-1 transcriptional cascades [20,86]. Beyond canonical kinase inhibition, these compounds demonstrate a vast pleiotropic repertoire. This includes endogenous NO donation via oxidoreductive bioconversion [16], direct modulation of inflammasome complexes through stable binding to the ASC [27], and profound DNA topology modulation. Notably, strategic derivatization, such as *O*-substitution, transition metal coordination (e.g., Ru, Pt, Cu, Zn), and spiro-heterocycle fusion, has consistently amplified their bioactivity. For instance, recent structural tuning has yielded derivatives that inhibit human Topo II with greater potency than the clinical standard etoposide [311], as well as spirooxindole–indenoquinoxaline hybrids that act as nanomolar TrpRS inhibitors, exhibiting dual antibacterial and antineoplastic efficacy [198]. Furthermore, the integration of metal complexes, such as Zn–curcumin–tryptanthrin hybrids, has been shown to drive nuclear accumulation, severe DNA damage, and apoptosis in drug-resistant carcinoma models [290].

Despite these substantial preclinical advances, the clinical development of tetracyclic ketoximes and their analogs is still limited by poor oral bioavailability, rapid systemic clearance, and context-dependent metabolic liabilities. The same rigid, planar, highly lipophilic architecture that supports high-affinity target binding also contributes to low aqueous solubility. Recent rational design efforts, however, have produced highly water-soluble derivatives that retain strong antiproliferative activity, indicating that these pharmacokinetic barriers can be addressed [311]. Localized and sustained delivery systems, including electrosprayed PLGA microparticles and electrospun PCL scaffolds, have also shown capacity to bypass systemic pharmacokinetic limitations while maintaining target engagement at inflammatory sites [173,174]. Resistance mechanisms mediated by pathogens and tumors, particularly efflux pump upregulation, further underscore the need for more innovative therapeutic strategies [29]. One promising countermeasure involves using tryptanthrin derivatives to target GSTP1, thereby inducing ROS-mediated cellular senescence. This process exposes vulnerabilities in resistant tumor cells and markedly sensitizes them to senolytic agents, such as ABT263 [30].

Future progress with tetracyclic ketoximes and related derivatives will likely depend on incorporating artificial intelligence-guided molecular design and comprehensive in silico ADMET prediction before chemical synthesis [323,327]. Strategic use of the oxime moiety for dual-action pharmacology, including the pairing of kinase inhibition with senolytic sensitization, NO-mediated vasodilation, or targeted metal chelation, provides a useful framework for next-generation MTDLs. As structural biology, computational thermodynamics, and in vivo disease modeling continue to clarify target engagement and tissue distribution, indenoquinoxalinone- and tryptanthrin-based oximes may advance from promising preclinical leads toward clinically validated therapeutics. Their ability to combine synthetic accessibility with broad pharmacological activity makes them valuable candidates for complex, multifactorial diseases in oncology, neurology, cardiology, and immunology.

## Figures and Tables

**Table 1:** Key neuroprotective findings for tetracyclic ketoximes with 11*H*-indeno[1,2-*b*]quinoxalin-11-one (IQ-1S and IQ-1L) and tryptanthrin (TRP and TRP-Ox) skeletons.

Compd.	Model/System	Route/Dose	Key Neuroprotective Outcomes and Mechanisms	Ref.
TRP-Ox	Rat, MCAO	i.p., 5–10 mg/kg	↓ Neurological deficit; ↓ infarct volume; ↓ IL-1β and TNF production; ↓ cerebral edema; ↓ c-Jun phosphorylation; anti-inflammatory; antioxidant; predicted BBB permeability	[88]
TRP-Ox, TRP	Rat, MCAO	i.p., 10 mg/kg	↓ Cerebral infarction size and brain tissue swelling; anti-inflammatory activity; neuroprotective	[89]
IQ-1S	Rat, MCAO	i.p., 5–25 mg/kg	↓ Infarct volume; faster neurological recovery; ↓ c-Jun phosphorylation in hippocampus; ↓ apoptotic signaling	[87]
IQ-1L	Rat, MCAO	i.p., 12 mg/kg	↓ Neurological deficit; ↓ infarct size; JNK1-3 binding affinity; Li^+^-mediated stabilization of oximate coordination geometry	[90]
IQ-1S	Mouse, MCAO	i.p., 25 mg/kg	↓ Neurological deficit; ↓ infarct size; NO release via oxidoreductive bioconversion of IQ-1S	[17]
IQ-1S	Rat, GCI	i.g., 50 mg/kg	Preserved hippocampal CA1 pyramidal neurons; ↓ blood hyperviscosity; ↓ endothelial dysfunction; ↓ lipid peroxidation; JNK inhibition; vasorelaxation	[91]
TRP-Ox	Brain tissue; SH-SY5Y cells; O_2_^−^ generation	0.25–5 µM (in vitro)	↓ Spontaneous CL in brain homogenate (0.25 µM); cytoprotective against oxidative stress (0.78–1.6 µM); antiradical/antioxidant activity (0.5–5 µM)	[18]
TRP	BV2 cells; Mouse, spinal cord injury	i.g., 30 mg/kg, 14 days	Microglial polarization to M2 (5–10 µM); ↓ neuroinflammation; neuronal apoptosis; inactivation of cGAS/STING/NF-κB axis	[95]
TRP	BV2 cells	20 µM (in vitro)	↓ iNOS, COX-2; ↓ TNF, IL-6, IL-1β production; ↓ M1 polarization; Nrf2/HO-1 signaling activation; ↓ NF-κB pathway	[96,97]
TRP	Rat, MCAO	i.p., 12 mg/kg	↓ Neurological deficit; ↓ infarct volume; ↓ IL-1β and TNF production; ↓ cerebral edema; ↓ BBB permeability	[94]
IQ-1S	OXYS rats	i.g., 50 mg/kg	↓ JNK3, c-Jun, APP, and tau phosphorylation in hippocampus; suppressed accelerated brain aging; JNK3 inhibition	[98]
IQ-1S	OXYS rats	i.g., 50 mg/kg	Improved retinal blood circulation; ↑ PEDF; ↓ VEGF; preserved retinal synapses and photoreceptor integrity; ↓ JNK pathway in retina; ↓ photoreceptor degeneration	[99,100]

Abbreviations: tryptanthrin, TRP; tryptanthrin-6-oxime, TRP-Ox; global cerebral ischemia, GCI; middle cerebral artery occlusion, MCAO.

**Table 2:** Cardioprotective and antihypertensive findings for tetracyclic ketoximes with 11*H*-indeno[1,2-*b*]quinoxalin-11-one (IQ-1 and IQ-1S) and tryptanthrin (TRP, 8-methyltryptanthrin (8-Me-TRP), and TRP-Ox) skeletons.

Compd.	Model	Route/Dose	Key Cardiovascular Outcomes and Mechanisms	Ref.
IQ-1S	SHR	i.g., 5–50 mg/kg/day	↓ Blood viscosity; ↓ myocardial cross-sectional area; ↓ aortic wall thickness; vasorelaxation; ↓ endothelin-1 secretion in HUVECs; ↓ endothelial vasoconstrictor production; JNK inhibition	[120]
IQ-1S	SHR	i.g., 50 mg/kg/day, 6 weeks	↓ LVM/BM index; ↓ LVEDP; JNK inhibition; attenuation of pressure-induced hypertrophic remodeling; preservation of diastolic compliance	[121]
IQ-1	Rat, MI	i.p., 25 mg/kg	↓ Infarct area; JNK inhibition; improved LV systolic and diastolic function	[126,127]
TRP-Ox	Rat, MI	i.p., 12 mg/kg	↑ Viable muscle elements in scar; ↓ connective tissue replacement; ↓ myocardial fibrosis; JNK inhibition; ↓ fibrotic remodeling	[128]
IQ-1	Rat liver and blood (EPR)	i.p., 50 mg/kg	(DETC)_2_-Fe^2+^-NO signal detected in liver; ↑ Hb-NO in blood	[16]
TRP	ApoE^−^/^−^ mice	i.g., 50–100 mg/kg	↓ VSMCs proliferation; ↑ contractile markers (α-SMA, calponin, SM22α); ↓ vimentin; ↓ serum lipid levels; ↓ atherosclerotic plaque formation; ↓ arterial intimal hyperplasia	[138]
TRP	Platelets; carotid artery	In vitro/ in vivo	↓ Platelet aggregation, spreading, clot retraction; ↓ carotid artery thrombus formation; ↓ Gp1bα expression	[140]
TRP	HUVECs	1 µM (in vitro)	↓ CXCL10 expression; ↓ RIG-I, MDA5, ISGs; ↓ STAT1 phosphorylation; suppression of TLR3-mediated IFN signaling in endothelium	[139]
TRP	HMEC-1; BALB/c mice	10–20 µM (in vitro); 20 µM s.c. in the Matrigel plug	↓ Endothelial proliferation and migration, ↓ Ang-1, PDGFB, MMP2; ↓ angiogenesis; ERK1/2 pathway inhibition	[141,142]
8-Me-TRP	P19CL6 cells	1 µM (in vitro)	Activation of pacemaker ion channels (Ca_v1.2, Ca_v3.1) and gap junction genes (GJA1, GJA5)	[143]

Abbreviations: LVM/BM, left ventricular mass/body mass; RIG-I, retinoic acid-inducible gene-I; MDA5, melanoma differentiation-associated protein 5; ISGs, interferon-stimulated genes; MMP2, matrix metalloproteinase; s.c., subcutaneously.

**Table 3:** Anti-inflammatory and immunomodulatory findings for tetracyclic ketoximes with 11*H*-indeno[1,2-*b*]quinoxalin-11-one (IQ-1, IQ-1S, and IQ-1L) and tryptanthrin (TRP, TRP-Ox, and their analogs) skeletons.

Compd.	Model/System	Route	Anti-Inflammatory Outcomes and Mechanisms	Ref.
IQ-1, IQ-1S	THP-1Blue and MonoMac-6	In vitro	↓ TNF (IC_50_ = 0.25 µM), IL-6 (IC_50_ = 0.6 µM), IL-1α, IL-1β, IL-10, IFN-γ, GM-CSF, and NO production; ↓ NF-κB/AP-1 activity; JNK1–3 binding	[19]
TRP-Ox, IQ-1	THP-1Blue and MonoMac-6	In vitro	K_d_ = 22–275 nM (JNK1/JNK3); ↓ IL-6; ↓ NF-κB/AP-1 activity	[20]
TRP-Ox analogs	THP-1Blue and MonoMac-6	In vitro	↓ IL-6, IL-1α, IL-1β, GM-CSF, MCP-1, and TNF production; ↓ NF-κB/AP-1 activity	[176]
TRP-Ox analogs	THP-1Blue and MonoMac-6	In vitro	↓ IL-6 production; ↓ NF-κB/AP-1 activity	[86]
IQ-1 analogs	THP-1Blue and MonoMac-6	In vitro	↓ IL-6; ↓ NF-κB/AP-1 activity; submicromolar JNK binding	[177]
IQ-1S	Human MΦ and T-cell cultures	In vitro (0.5–25 µM)	↓ CD197^+^ macrophages; ↓ TNF, IL-1β, IL-6, IL-10, IL-2, IFN-γ, and IL-4 production; ↓ CD25^+^ T cells; ↓ MΦ and T-cell activation	[155]
IQ-1S	Murine LPS/D-GalN–sepsis	i.p.	↓ Lung inflammation; ↓ TNF, IL-6, IL-1β; ↓ M1 MΦ polarization; ↓ NF-κB, AP-1 and NF-Y activation	[154]
IQ-1S	Murine CIA (DBA/1 mice)	i.p. (20–50 mg/kg, daily), in vitro	↓ Clinical arthritis scores; ↓ cartilage loss; ↓ collagen II-specific antibodies; ↓ proinflammatory cytokines in joints and lymph nodes; ↑ Foxp3^+^CD4^+^CD25^+^ Tregs; JNK inhibition; MMP-1/3 gene expression suppression	[159]
TRP, TRP-Ox	CIA and CAIA; FLS, HUVECs, THP-1Blue	i.p. (30 mg/kg daily), in vitro	↓ CIA/CAIA clinical scores; ↓ collagen II-specific antibodies; ↓ MMP-1/3, IL-6 secretion; oxime more potent for IL-17A, GM-CSF, RANKL suppression in vitro; JNK inhibition (for TRP-Ox)	[160]
IQ-1L	THP-1Blue; MonoMac-6	i.p. (12 mg/kg), in vitro	↓ NF-κB/AP-1; ↓ c-Jun phosphorylation; ↓ proinflammatory cytokines	[90]
IQ-1	Neutrophils; THP-1Blue; MonoMac-6	In vitro	↓ Neutrophil ROS production and Ca^2+^ mobilization; ↓ LPS-induced NF-κB/AP-1 activity; ↓ IL-6 production	[174]
IQ-1	Neutrophils; MonoMac-6	In vitro	↓ Neutrophil activation; ↓ LPS-induced IL-6 production	[173]
TRP	RAW 264.7	1–30 µM (in vitro)	↓ NO production; ↓ COX; ↓ iNOS expression	[148]
TRP	Mono Mac 6; neutrophils	In vitro	COX-2 inhibition (IC_50_ = 37 nM); ↓ LTB_4_ release (IC_50_ = 150 nM); dual COX-2/5-LOX pathway blockade	[145,146,147]
TRP analog	RAW264.7; AIA in rats	i.g.; in vitro	IC_50_ = 1.25 µM (NO); ↓ IL-1β (IC_50_ = 8.5 μM), TNF (IC_50_ = 11.5 μM), iNOS, COX-2; ↓ paw swelling and joint pathology in AIA; p38α MAPK inhibition	[149]
TRP	RAW264.7; DSS-ind. colitis	i.g. 78.4 mg/kg, 8 days	↓ NO, IL-6, and TNF production; ↓ NF-κBp65; ↑ IκBα; ↓ p-STAT3; ↓ TNF/NF-κB and IL-6/STAT3 pathways	[165]
TRP	HaCaT cells; psoriatic mice	20 and 50 nM; i.g., 25 and 100 mg/kg, 7 days	↓ PASI; ↓ Th17 infiltration; ↓ inflammatory cytokines; ↑ GSH, SOD, CAT activity; MAPK (JNK, ERK1/2, p38) suppression; Nrf2 antioxidant pathway activation	[166]
TRP	Murine IPF model; NIH3T3 fibroblasts	i.g., 5–10 mg/kg	Improved pulmonary function; ↓ fibrosis; ↓ TNF, IL-1β, IL-6 production; ↓ fibronectin; dual suppression of MAPK/NF-κB and TGF-β1/Smad2/3 cascades	[171]

Abbreviations: AIA, adjuvant-induced arthritis; CIA, collagen-induced arthritis; CAIA, collagen-antibody-induced arthritis; CAT, catalase; DSS-ind. colitis, dextran sodium sulfate-induced colitis; IPF, idiopathic pulmonary fibrosis; MΦ, macrophage.

**Table 4:** Antitumor findings for tetracyclic ketoximes and related compounds.

Compound	Cell Lines/Model	Cytotoxicity and Mechanisms of Action	Ref.
*Organic Compounds*			
11*H*-indeno[1,2-*c*]quinolin-11-ones and oximes	HeLa, SAS, SKHep, AGS, A549	GI_50_ = 0.4–0.9 μM; active against AGS and A549 cells; DNA intercalation; S-phase arrest	[178]
6-Alkylamino- and 6-arylamino-9-methoxy-11*H*-indeno[1,2-*c*]quinolin-11-ones and oximes	H460, HeLa, SKHep, AGS, PC-3, A549	GI_50_ = 3.4–3.5 μM; DNA intercalation; G2/M arrest; caspase-3/-8 activation	[201]
6-Hydroxy-9-methoxy-11*H*-indeno[1,2-*c*]quinolin-11-one derivative	SAS, A549, BT483; BT483 nude mouse xenograft	GI_50_ = 0.8–0.9 μM; in vivo tumor regression; dual Topo I/II inhibition; DNA intercalation; S-phase arrest; caspase-3 activation	[179]
TCH-1030	MCF-7, BT483; BT483 xenograft	S-phase arrest; tumor regression (in vivo); Topo I targeting; DNA intercalation	[180]
*Metal Coordination Complexes*			
Arene-Ru(II) complexes with TRP-Ox	MCF-7, MCF-7CR, MCF10A	IC_50_ ≈ 9.0 μM (MCF-7); 8.9 μM (MCF-7CR); *N*,*N*-bidentate anionic oximate coordination	[184]
Arene-Ru(II) complexes with IQ-1 derivatives and TRP-Ox	PANC-1	Moderate cytotoxic effect; anionic oximate coordination	[185]
[Pt(BrTRP)(DMSO)Cl_2_]	BEL-7402, MGC80-3, HepG2; HL-7702	IC_50_ = 0.2 μM (T-24); telomerase inhibition; S-phase arrest; ↓ cyclin A/CDK2	[186]
[Cu(TRP)_2_Cl_2_] and [Cu(BrTRP)_2_Cl_2_]	BEL-7402, MGC80-3, HepG2; HL-7702	IC_50_ = 4–9 μM; DNA damage; S-phase arrest; telomerase inhibition via c-Myc promoter	[188]
*Spiro-Heterocycle Conjugates and Multi-Targeted Hybrids*			
Spiro[TRP-cyclopropa[*a*]pyrrolizine] derivatives	K562, HeLa, CT26	Cytotoxicity	[69]
Spiro-fused pyrrolo[3,4-*a*]pyrrolizine-tryptanthrins	K562, HeLa	Active against K562 and HeLa; G2/M accumulation; apoptosis; actin filament depolymerization; ↓ cell motility	[189]
Spiro[indenoquinoxaline-pyrrolizidine]-*N*-arylpyrazoles	HeLa	High cytotoxic activity	[192]
Spiro-indenoquinoxaline pyrrolothiazoles and pyrrolizines	MCF-7, A-549	Significant activity comparable to doxorubicin; apoptosis and necrosis	[190,191]
TRP-1,2,3-triazole hybrids	Cancer cell lines	Promising in vitro anticancer potency; favorable ADMET profile	[197]
Spirooxindole–indenoquinoxalines	SU-DHL-4, SU-DHL-6, OCI-Ly10	IC_50_ = 2.9–4.8 μM (DLBCL); TrpRS inhibition	[198]
Spiro-fused pyrrolo[3,4-*a*]pyrrolizine-tryptanthrins	MCF-7, MDA-MB-231, HeLa, PC-3	IC_50_ = 6.6–18 μM; non-toxic to HEK-293; EGFR binding (−10.2 to −11.5 kcal/mol)	[196]
Spiro-indenoquinoxaline-based pyrazoles	HepG2, MCF-7	IC_50_ = 1.45 μM (HepG2); dual EGFR/VEGFR-2 inhibition; G1-phase arrest	[36]

## Data Availability

No new data were created or analyzed in this study. Data sharing is not applicable to this article.

## Supplementary Materials

The following supporting information can be downloaded at https://www.mdpi.com/article/10.3390/molecules31173032/s1. Table S1. Antimicrobial, antiviral, antiparasitic, antitubercular, and antidiabetic effects of tetracyclic ketoximes and related compounds. Table S2. Alternative (non-kinase) mechanisms of therapeutic action for tetracyclic ketoximes and related tryptanthrins. Refs. [21,22,23,27,29,30,34,35,95,167,168,169,198,203,205,208,209,210,211,220,221,222,226,227,228,229,231,233,237,238,239,240,280,281,284,285,287,288,289,297,298,299,300,303,305,306,307,308,328,329,330,331,332,333] are cited in the Supplementary Materials.
