# Supplementary material for: Chemistry and Biological Activity of 11H-Indeno[1,2-b]quinoxalin-11-ones and Tryptanthrins, Their Oximes, and Related Analogues
Source: Molecules. 2026 Aug 28;31(17):3032. doi: 10.3390/molecules31173032 (PMC13567725; doi:10.3390/molecules31173032)
Supplement: Supplementary file 1 [file molecules-31-03032-s001.zip › molecules-4498043-supplementary.pdf]

Supplementary Materials
for Review

Chemistry and Biological Activity of 11*H*-Indeno[1,2-*b*]quinoxalin-11-ones and Tryptanthrins, Their Oximes, and Related Analogues

Igor A. Schepetkin [1,*], Mark B. Plotnikov [2], Anastasia R. Kovrizhina [3] and Andrei I. Khlebnikov [3,*]

[1] Department of Plant Science and Plant Pathology, Montana State University, Bozeman, MT 59717, USA

[2] Department of Pharmacology, Goldberg Research Institute of Pharmacology and Regenerative Medicine, Tomsk National Research Medical Center, Russian Academy of Sciences, Tomsk 634028, Russia; mbp2001@mail.ru

[3] Kizhner Research Center, Tomsk Polytechnic University, Tomsk 634050, Russia; anaskowry@gmail.com

* Correspondence: igor@montana.edu (I.A.S.); aikhl@chem.org.ru (A.I.K.)

Table S1. Antimicrobial, antiviral, antiparasitic, antitubercular, and antidiabetic effects of tetracyclic ketoximes and related compounds.

Compd.	Pathogen / Model	Key Activity Data and Proposed Mechanism(s)	Ref.
**Antimicrobial / Antifungal**			
TRP-Ox and 4-aza derivatives	*Escherichia coli* (growth, viability, mutagenesis)	Viability reduction by factors of 10^{-2} to $<10^{-6}$ at 0.2–40 μM; 6-oximo derivatives most bactericidal; DNA intercalation; disruption of replication and transcription	[1]
TRP analogues	*Xanthomonas oryzae* pv. *oryzae* (Xoo); TMV; citrus canker model	EC_{50} = 1.2 μg/mL vs. Xoo (>25-fold more potent than bismerthiazol and thiodiazole copper); ROS accumulation; bacterial apoptosis; biofilm disruption; T2SS/T6SS secretion system inhibition	[2]
TRP derivatives (broad panel)	*Physalospora piricola*; 14 phytopathogenic fungi; TMV	Broad-spectrum fungicidal activity; selectivity to *P. piricola* surpassing conventional agrochemicals; anti-TMV *in vivo*; TMV CP disk decomposition	[3]
Spiro-indenoquinoxaline heterocycles	Gram-positive bacteria (*S. aureus*)	Enhanced membrane permeation; 50% inhibitory effect; selectivity for Gram-positive pathogens	[4]
Indenoquinoxaline thiocarbohydrazones	*S. aureus*, *C. albicans*	Promising antimicrobial activity with concurrent radical-scavenging profiles; molecular modeling (B3LYP/6-31G) supports DNA/protein interaction	[5]
Pd(II)–quinoxaline Schiff base complex	*S. aureus*, *C. albicans*	Antimicrobial + antioxidant (ABTS, FRAP assays); square-planar coordination; enhanced bioactivity vs. free ligand	[6]
**Antiviral**			
TRP	HCoV-NL63 (LLC-MK2 cells)	IC_{50} = 1.5 μM (virus yield); virucidal IC_{50} = 60 nM; blocks early and late replication stages; inhibition of viral RNA genome synthesis; papain-like protease 2 (PLpro2) activity blockade	[7]
TRP derivatives	SARS-CoV-2 (Vero cells); Mpro; PLpro	Fe(III/II) chelation; suppression of viral replication in vitro; high docking affinity for Mpro and PLpro; iron chelation relevant to COVID-19 pathogenesis	[8]
TRP	IAV: wild-type H1N1, H3N2	Protective effects against both wild-type and resistant IAV strains (CPE reduction assay); multi-target antiviral; network pharmacology identifies TRP as key anti-IAV agent	[9,10]
TRP derivative B1	Potato Virus Y (PVY) VPg protein	K_d = 0.7 μM (MST); 4.0 μM (ITC); suppresses PVY gene expression in vivo; binds VPg from 3 other Potyviruses	[11]
**Antiparasitic / Antiprotozoal**			
TRP derivatives	*Toxoplasma gondii*	Low nanomolar parasite growth inhibition; low host cell cytotoxicity; alcohol analogs most promising; C-6 modification critical for activity; oxime/hydrazone analogs enhance inhibition	[12,13]
TRP	*Leishmania infantum*; *L. amazonensis*	IC_{50} = 8 μM (*L. infantum*), 11 μM (*L. amazonensis*) promastigotes; active against amastigotes; mitochondrial membrane depolarization; apoptosis-like death	[14]
Indolo[2,1-*b*]quinazoline-6,12-diones	*L. donovani*	Active at concentrations <100 ng/mL; low toxicity to macrophage and neuronal lines; electron transfer to carbonyl/oxime region	[15]

TRP analogs (8-substituted)	*Trypanosoma brucei in vitro*	EC_{50} = 0.4 μM (most potent; 8-position electron-withdrawing group); electron-withdrawing substituents at C-8 enhance activity	[16]
Imidazoquinolinone C1	*Trypanosoma cruzi*	IC_{50} epi = 1.5 μM; IC_{50} amas = 1.7 μM; IC_{50} try = 34.9 μM; low host cell toxicity; reduction by intracellular thiol pool → oxidative stress → parasite apoptosis; antioxidant co-treatment enhances activity 3-fold	[17]
TRP derivatives (NT1, T8)	*Plasmodium falciparum*	IC_{50} = 30 nM (NT1; SI = 156); nanomolar activity (30–100 nM) retained against resistant strains; gametocyte maturation blocked; NT1 reduces exflagellation	[18]
TRP	*P. falciparum* 3D7 and Dd2	IC_{50} = 2.5 μM (3D7), 3.1 μM (Dd2); CC_{50} > 10 μM; no hemolysis at 100 μg/mL; antiplasmodial	[19]
Quinoxaline analogs	*Schistosoma mansoni*	IC_{50} ≤ 0.3 μM (adult *S. mansoni*); SI ≥ 8.9; moderate WBR *in vivo* (9–46% at 400 mg/kg)	[20]
		**Antitubercular**	
TRP analogs	Mtb (MABA assay; acute murine TB)	Maintained *in vitro* Mtb activity; improved solubility and oral bioavailability vs. parent; failed to suppress Mtb replication *in vivo* (up to 400 mg/kg/day × 4 weeks); illuminates SAR path but highlights PK disconnect; first systematic ADME profiling of TRP class	[21]
2-(Propylthio)-5*H*-[1,3,4]thiadiazolo[2,3-*b*]quinazolin-5-one	*M. tuberculosis*	MIC = 6.5 μg/mL (100% growth inhibition); very low oral toxicity (GHS category 5); InhA (enoyl-ACP reductase) inhibition; stable binding within InhA active site	[22]
C-6 keto-modified TRP	MDR *M. tuberculosis*; InhA	Promising activity against MDR-TB; high InhA affinity; InhA inhibition; favorable predicted PK profiles	[23]
Spiropyrrolidine-tethered indenoquinoxaline	*M. tuberculosis* H37Rv	MIC = 1.6 μg/mL; ionic-liquid-accelerated multicomponent 1,3-dipolar cycloaddition	[24]
		**Antidiabetic / Metabolic**	
Hydrazinyl thiazole–linked indenoquinoxaline hybrids	*α*-Amylase; *α*-glucosidase	IC_{50} = 0.3–77 μM (*α*-amylase); IC_{50} = 1.1–92 μM (*α*-glucosidase); outperform acarbose (IC_{50} = 13.5 μM); DPPH SC_{50} = 7.58–125.86 μM	[25]
Pd(II)–quinoxaline Schiff base complex	*α*-Amylase (enzyme inhibition + MD simulation)	IC_{50} = 326.5 μg/mL; mixed-mode inhibition kinetics; stable ligand–enzyme interactions (100 ns MD)	[6]

Abbreviations: ABTS, 2,2′-azino-bis(3-ethylbenzothiazoline-6-sulfonic acid); ADMET, absorption, distribution, metabolism, excretion, and toxicity; ACP, acyl carrier protein; BLG, Banlangen; CP, coat protein; CPE, cytopathic effect; CQ, chloroquine; DPPH, 2,2-diphenyl-1-picrylhydrazyl; EC_{50}, half-maximal effective concentration; FRAP, ferric reducing antioxidant power; GHS, Globally Harmonized System; HCoV-NL63, human coronavirus NL63; IAV, influenza A virus; IC_{50}, half-maximal inhibitory concentration; InhA, enoyl-acyl carrier protein reductase; ITC, isothermal titration calorimetry; Kd, dissociation constant; MABA, microplate Alamar blue assay; MD, molecular dynamics; MDR, multidrug resistant; MM/GBSA, molecular mechanics/generalized Born surface area; Mpro, main protease; MST, microscale thermophoresis; NTS, newly transformed schistosomula; PK, pharmacokinetic; PLpro, papain-like protease; PVY, Potato Virus Y; QM, quantum mechanical; ROS, reactive oxygen species; SAR, structure–activity relationship; SC_{50}, half-maximal scavenging concentration; SI, selectivity index; T2SS/T6SS, type II/VI secretion systems; TMV, tobacco mosaic virus; VPg, viral genome-linked protein; WBR, worm burden reduction.

Table S2. Alternative (non-kinase) mechanisms of therapeutic action for tetracyclic ketoximes and related tryptanthrins.

Compd.	Model / System	Key Findings and Molecular Target / Pathway	Ref.
TRP	MCD-induced NASH mice; LPS-induced sepsis mice	Inhibits NLRP3, NLRC4, and AIM2 inflammasomes; ameliorates NASH and sepsis *in vivo*; PYD binding; disrupts ASC oligomerization and caspase-1 maturation	[26]
TRP	BV2 microglia (LPS-stimulated); mouse SCI model	M1→M2 microglial polarization; ↓ neuroinflammation; suppressed ER stress–related neuronal apoptosis; ↑ functional recovery cGAS/STING/NF-κB axis inactivation	[27]
TRP	HaCaT keratinocytes; fibroblasts infected with *T. benhamiae* DSM6916	↑ HBD2 and HBD3 antimicrobial peptide expression; ↓ pro-inflammatory cytokines; prevented dermatophyte-induced cellular damage; immune modulation via AMP upregulation; TLR2 pathway	[28]
TRP	HMC-1 human mast cells; NC/Nga mice; splenocytes	↓ TSLP production and mRNA expression; ↓ IL-4, IFN-γ, TNF; ↓ histidine decarboxylase; ameliorated clinical atopic dermatitis symptoms; ↓ RIP2/caspase-1/NF-κB pathway	[29]
TRP	HMC-1 cells (TSLP-stimulated)	↓ Mast cell proliferation; ↑ p53, PARP, caspase-3; ↓ MDM2, Ki67; ↓ IL-13 and TNF; MDM2/p53 axis modulation; TSLP receptor downregulation	[30]
Pityriazepin and indole metabolites	Recombinant AhR reporter cell lines	Potent AhR agonism; competitive binding; direct stimulation of AhR-dependent DNA binding; species-specific potency	[31]
Indigo naturalis (TRP as key component)	Plaque psoriasis patients	PASI 75 response in 56.3% vs. 0% placebo; gene expression signature returned toward normal; ↓ IL-17 pathway; IL-17 pathway suppression; TRP-mediated IL-17 inhibition in keratinocyte assay	[32]
6-Oximo and 4-aza TRP derivatives	*E. coli* DNA; computational DFT modeling	Preferential intercalation into GC-rich DNA (ΔE ≈ 2–4 kcal/mol GC vs. TA); disruption of replication and transcription; DNA intercalation; GC-selective π-stacking	[1,37]
TRP sulfonate (TPTS)	Calf thymus DNA (UV spectroscopy)	$K_{bind} = 1.10 \times 10^4$ M^{-1}; moderate hypochromicity; significant viscosity increase; Intercalative DNA binding mode	[38]
Aminoalkylamino indolo[2,1-*b*]quinazolin-6,12-diones	Synthetic DNA triplex structures	↑ Triplex thermal stability by up to 6 °C; position-8 F or N-CH_3 substitution enhances stabilization additively; DNA triplex stabilization via side-chain orientation	[39]
Benzo[*b*]tryptanthrin	HCT15 colon; MCF7/adr breast cancer cells	Non-intercalative ATP-competitive dual topo I/II catalytic inhibitor; reverses adriamycin resistance; ↓ MDR1 expression; topoisomerase I/II catalytic inhibition; MDR1 downregulation	[40,41]
Mostotrin (TRP derivative)	DNA fluorescence quenching assay; tumor cell lines	DNA binding confirmed by fluorescence quenching; 5–10× higher cytotoxicity vs. parent TRP; LD_{50} = 375 mg/kg;	[42]

		DNA binding; improved cellular uptake via enhanced aqueous solubility	
TRP	HepG2; Huh7 liver cancer cells; xenograft	Directly binds and inhibits GSTP1; induces ROS accumulation, DDR, NF-κB–mediated SASP; sensitizes to senolytic ABT263; GSTP1/ROS/DDR/NF-κB/SASP axis → cellular senescence	[43]
Nano-encapsulated CY-1-4 (TRP derivative)	B16-F10 melanoma (*in vitro* and *in vivo*)	IDO inhibition + ICD induction as single agent; ↓ IDO expression in tumor tissue; regulated lymphocyte subsets; ↓ 58% tumor burden; dual IDO inhibition and immunogenic cell death	[44]
CIQ (8-cyanoindolo[2,1-*b*]quinazoline-6,12-dione)	MDA-MB-231; MCF-7 breast cancer cells	↑ p-ERK, p-JNK, p-p38; caspase-dependent apoptosis; ↓ invasion; ↓ MMP-2 and Snail; ↑ γ-H2AX (DNA damage); pro-apoptotic MAPK (ERK/JNK/p38) activation; ROS generation	[45]
8-Fluorotryptanthrin	K-562/4; HCT-116; HCT-116p53ko	Higher cytotoxicity than parent TRP; cell cycle arrest at G_1→S transition at nanomolar concentrations; cell cycle blockade at G_1/S phase	[46]
TRP	*V. splendidus* AJ01	↑ Biofilm but ↓ motility, hemolytic activity, and flagellar gene expression; competitive binding to $LuxO_1$	[47]
TRP	Toxigenic *V. cholerae*	↓ Biofilm maturation at sub-MIC (2 μg/mL); synergistic with ciprofloxacin; non-cytotoxic; LuxO quorum-sensing regulator targeting	[48]
Fluorinated/piperazine TRP analogs	*X. oryzae* pv. *oryzae*; citrus canker model	EC_{50} = 1.26 μg/mL (>25× more potent than bismerthiazol); ↓ T2SS/T6SS pathways; ↓ membrane transport; secretion system disruption (T2SS/T6SS; proteomics-confirmed)	[43]
TRP	*C. jejuni* (*in vitro*; chick infection model)	Bactericidal activity; narrow antimicrobial spectrum; low resistance emergence potential; ↓ cecal colonization in chicks; direct bactericidal mechanism; MIC far lower than other herbal compounds tested	[49]
TRP	*C. neoformans*; *T. rubrum*	MIC = 2 μg/mL (*Cryptococcus*); cell cycle arrest at G1/S; synergy with FK506 and CsA; cell cycle regulators (CLN1, MBS1, PCL1, WHI5); Pmc1 calcium transporter; PKA signaling	[50]
Spirooxindole–indenoquinoxaline	ecTrpRS; *S. aureus* (MRSA); DLBCL cell lines	IC_{50} = 74 nM (ecTrpRS); MIC_{90} = 4 μg/mL (*S. aureus*); IC_{50} = 2.9–4.8 μM (DLBCL); bacterial autolysis induction; TrpRS inhibition; dual antibacterial/anticancer activity	[51]
5-Substituted TRPs	20S proteasome	Unprecedented inhibition of 20S proteasome activity	[52]
TRP	*M. smegmatis* (spontaneous resistant mutants)	Resistance via MmpS5-MmpL5 efflux upregulation; MSMEG_1963 (EmbR) high-level resistance; MSMEG_5597 (TetR) low-level resistance;<br>Efflux-mediated resistance (not target-site alteration); MFS transporter involvement	[53]
TRP	BALB-C mice (excisional wound model)	Wound closure rate up to 94% (highest dose); ↑ VEGF and MMP-9 on days 3 and 7; histopathological improvement; VEGF/MMP-9–mediated tissue repair and angiogenesis	[54]

Abbreviations: AD, atopic dermatitis; AhR, aryl hydrocarbon receptor; AMP, antimicrobial peptide; ASC, apoptosis-associated speck-like protein containing a CARD; BMDMs, bone marrow-derived macrophages; cGAS, cyclic GMP-AMP synthase; CsA, cyclosporine A; DDR, DNA damage response; DLBCL, diffuse large B-cell lymphoma; DSS, dextran sulfate sodium; ecTrpRS, Escherichia coli tryptophanyl-tRNA synthetase; ER, endoplasmic reticulum; FICI, fractional inhibitory concentration index; GluR6, glutamate receptor 6; GSTP1, glutathione S-transferase P1; HBD, human β-defensin; ICD, immunogenic cell death; IDO, indoleamine 2,3-dioxygenase; IL, interleukin; MCD, methionine- and choline-deficient diet; MDR1, multidrug resistance protein 1; MFS, major facilitator superfamily; MIC, minimum inhibitory concentration; MLK3, mixed lineage kinase 3; MMP, matrix metalloproteinase; MRSA, methicillin-resistant Staphylococcus aureus; NASH, non-alcoholic steatohepatitis; NF-κB, nuclear factor-κB; NLRP3, nod-like receptor family pyrin domain containing 3; PARP, poly(ADP-ribose) polymerase; PASI, psoriasis area and severity index; PKA, protein kinase A; PSD-95, postsynaptic density protein 95; PYD, pyrin domain; RCT, randomized controlled trial; RIP2, receptor-interacting protein 2; ROS, reactive oxygen species; SASP, senescence-associated secretory phenotype; SCI, spinal cord injury; STING, stimulator of interferon genes; T2SS/T6SS, type II/VI secretion systems; TLR, Toll-like receptor; TNF, tumor necrosis factor-α; topo, topoisomerase; TrpRS, tryptophanyl-tRNA synthetase; TSLP, thymic stromal lymphopoietin; VEGF, vascular endothelial growth factor.

References

1. Bandekar, P.P.; Roopnarine, K.A.; Parekh, V.J.; Mitchell, T.R.; Novak, M.J.; Sinden, R.R. Antimicrobial activity of tryptanthrins in Escherichia coli. *J. Med. Chem.* **2010**, *53*, 3558-3565, doi:10.1021/jm901847f.
2. Zhang, G.L.; Wang, Z.C.; Li, C.P.; Chen, D.P.; Li, Z.R.; Li, Y.; Ouyang, G.P. Discovery of tryptanthrin analogues bearing F and piperazine moieties as novel phytopathogenic antibacterial and antiviral agents. *Pest. Manag. Sci.* **2024**, *80*, 1026-1038, doi:10.1002/ps.7834.
3. Hao, Y.N.; Guo, J.C.; Wang, Z.W.; Liu, Y.X.; Li, Y.Q.; Ma, D.J.; Wang, Q.M. Discovery of tryptanthrins as novel antiviral and anti-pytopathogenic-fungus agents. *J. Agric. Food Chem.* **2020**, *68*, 5586-5595, doi:10.1021/acs.jafc.0c02101.
4. Kumar, N.; Inwati, G.K.; Ahmed, E.M.; Lal, C.; Makwana, B.; Yadav, V.K.; Islam, S.; Ahn, H.J.; Yadav, K.K.; Jeon, B.H. Modified 7-chloro-11H-indeno[1,2-b]quinoxaline heterocyclic system for biological activities. *Catalysts* **2022**, *12*, doi:10.3390/catal12020213.
5. Abd El Salam, H.A.; El-Bendary, M.A.; Ibrahim, M.; El-Samahy, F.A. Synthesis, molecular modeling and biological evaluation of indeno[1,2-b]quinoxaline derivatives as antifungal and antibacterial agents. *Egypt. J. Chem.* **2020**, *63*, 2577-2590, doi:10.21608/ejchem.2020.21366.2275.
6. Periyannan, M.; Selvi, A.; Rajavel, R. Palladium (II)-quinoxaline complex as a potent antioxidant, antimicrobial, and antidiabetic agent: design, synthesis, in vitro and in silico evaluation. *Int. Res. J. Multidiscip. Technovation.* **2026**, *8*, 73-93, doi:org/10.54392/irjmt2635.
7. Tsai, Y.C.; Lee, C.L.; Yen, H.R.; Chang, Y.S.; Lin, Y.P.; Huang, S.H.; Lin, C.W. Antiviral action of tryptanthrin isolated from *Strobilanthes cusia* leaf against human coronavirus NL63. *Biomolecules* **2020**, *10*, doi:10.3390/biom10030366.
8. Veselá, K.; Melková, Z.; Abramenko, N.; Kejík, Z.; Kaplánek, R.; Dytrych, P.; Sinica, A.; Vozniuk, O.; Martásek, P.; Jakubek, M. Preparation of tryptanthrin derivates bearing a thiosemicarbazone moiety to inhibit SARS-CoV-2 replication. *Separations* **2023**, *10*, doi:10.3390/separations10020073.

9. Cai, C.P.; Xu, L.; Fang, J.F.; Dai, Z.; Wu, Q.H.; Liu, X.Y.; Wang, Q.; Fang, J.S.; Liu, A.L.; Du, G.H. In silico prediction and bioactivity evaluation of chemical ingredients against influenza A virus from *Isatis tinctoria* L. *Front. Pharmacol.* **2021**, *12*, doi:10.3389/fphar.2021.755396.
10. Xu, L.J.; Jiang, W.; Jia, H.; Zheng, L.S.; Xing, J.G.; Liu, A.L.; Du, G.H. Discovery of multitarget-directed ligands against influenza A virus from compound Yizhihao through a predictive system for compound-protein interactions. *Front. Cell. Infect. Microbiol.* **2020**, *10*, doi:10.3389/fcimb.2020.00016.
11. Wu, Z.L.; Zhang, C.; Huang, Y.Q.; Tao, N.; Wang, T.; Cai, X.B.; Wang, Z.C.; Li, X.Y. Tryptanthrin derivative B1 binds viral genome-linked protein (VPg) of potato virus Y. *J. Agric. Food Chem.* **2024**, *72*, 5699-5709, doi:10.1021/acs.jafc.4c01306.
12. Krivogorsky, B.; Grundt, P.; Yolken, R.; Jones-Brando, L. Inhibition of *Toxoplasma gondii* by indirubin and tryptanthrin analogs. *Antimicrob. Agents Chemother.* **2008**, *52*, 4466-4469, doi:10.1128/aac.00903-08.
13. Krivogorsky, B.; Nelson, A.C.; Douglas, K.A.; Grundt, P. Tryptanthrin derivatives as *Toxoplasma gondii* inhibitors-structure-activity-relationship of the 6-position. *Bioorg. Med. Chem. Lett.* **2013**, *23*, 1032-1035, doi:10.1016/j.bmcl.2012.12.024.
14. Garcia, A.R.; Silva-Luiz, Y.P.G.; Alviano, C.S.; Alviano, D.S.; Vermelho, A.B.; Rodrigues, I.A. The natural alkaloid tryptanthrin induces apoptosis-like death in *Leishmania* spp. . *Trop. Med. Infect. Dis.* **2022**, *7*, doi:10.3390/tropicalmed7060112.
15. Bhattacharjee, A.K.; Skanchy, D.J.; Jennings, B.; Hudson, T.H.; Brendle, J.J.; Werbovetz, K.A. Analysis of stereoelectronic properties, mechanism of action and pharmacophore of synthetic indolo 2,1-b quinazoline-6,12-dione derivatives in relation to antileishmanial activity using quantum chemical, cyclic voltammetry and 3-D-QSAR CATALYST procedures. *Bioorg. Med. Chem.* **2002**, *10*, 1979-1989, doi:10.1016/s0968-0896(02)00013-5.
16. Scovill, J.; Blank, E.; Konnick, M.; Nenortas, E.; Shapiro, T. Antitrypanosomal activities of tryptanthrins. *Antimicrob. Agents Chemother.* **2002**, *46*, 882-883, doi:10.1128/aac.46.3.882-883.2002.
17. Abadi, A.H. 5-Substituted 2-bromoindolo[3,2-b]quinoxalines.: A class of potential antitumor agents with cdc25 phosphatase inhibitory properties. *Arch. Pharm.* **1998**, *331*, 352-358, doi:10.1002/(sici)1521-4184(199811)331:11<352::Aid-ardp352>3.0.Co;2-2.
18. Onambele, L.A.; Riepl, H.; Fischer, R.; Pradel, G.; Prokop, A.; Aminake, M.N. Synthesis and evaluation of the antiplasmodial activity of tryptanthrin derivatives. *Int. J. Parasitol. Drugs Drug. Resist.* **2015**, *5*, 48-57, doi:10.1016/j.ijpddr.2015.03.002.
19. Abd Karim, H.A.; Ilmi, H.; Widyawaruyanti, A.; Ismail, N.H.; Zohdi, R.M.; Osman, C.P. Phytochemical analysis and evaluation of antiplasmodial and cytotoxic activities of *Wrightia dubia* and *Wrightia pubescens* traditionally used for malaria treatment in Southeast Asia. *Fitoterapia* **2025**, *186*, doi:10.1016/j.fitote.2025.106794.
20. Debbert, S.L.; Hintz, M.J.; Bell, C.J.; Earl, K.R.; Forsythe, G.E.; Häberli, C.; Keiser, J. Activities of quinoxaline, nitroquinoxaline, and [1,2,4]triazolo[4,3-a]quinoxaline analogs of MMV007204 against *Schistosoma mansoni*. *Antimicrob. Agents Chemother.* **2021**, *65*, doi:10.1128/aac.01370-20.
21. Hwang, J.M.; Oh, T.; Kaneko, T.; Upton, A.M.; Franzblau, S.G.; Ma, Z.K.; Cho, S.N.; Kim, P. Design, synthesis, and structure-activity relationship studies of tryptanthrins as antitubercular agents. *J. Nat. Prod.* **2013**, *76*, 354-367, doi:10.1021/np3007167.
22. Duca, G.; Pogrebnoi, S.; Boldescu, V.; Aksakal, F.; Uncu, A.; Valica, V.; Uncu, L.; Negres, S.; Nicolescu, F.; Macaev, F. Tryptanthrin analogues as inhibitors of enoyl-acyl carrier protein reductase: activity against *Mycobacterium tuberculosis*, toxicity, modeling of enzyme binding. *Curr. Top. Med. Chem.* **2019**, *19*, 609-619, doi:10.2174/1568026619666190304125740.

23. Kamal, A.; Reddy, B.V.S.; Sridevi, B.; Ravikumar, A.; Venkateswarlu, A.; Sravanthi, G.; Sridevi, J.P.; Yogeeswari, P.; Sriram, D. Synthesis and biological evaluation of phaitanthrin congeners as anti-mycobacterial agents. *Bioorg. Med. Chem. Lett.* **2015**, *25*, 3867-3872, doi:10.1016/j.bmcl.2015.07.057.
24. Arumugam, N.; Almansour, A.; Kumar, R.S.; Alaqeel, S.I.; Krishna, V.S.; Sriram, D. Anti-tubercular activity of novel class of spiropyrrolidine tethered indenoquinoxaline heterocyclic hybrids. *Bioorg. Chem.* **2020**, *99*, doi:10.1016/j.bioorg.2020.103799.
25. Hameed, S.; Khan, K.M.; Salar, U.; Özil, M.; Baltas, N.; Saleem, F.; Qureshi, U.; Taha, M.; Ul-Haq, Z. Hydrazinyl thiazole linked indenoquinoxaline hybrids: potential leads to treat hyperglycemia and oxidative stress; multistep synthesis, α-amylase, and antioxidant activities. *Int. J. Biol. Macromol.* **2022**, *221*, 1294-1312, doi:10.1016/j.ijbiomac.2022.09.102.
26. Ren, L.T.; Yang, H.J.; Wang, H.B.; Qin, S.L.; Zhan, X.Y.; Li, H.; Wei, Z.Y.; Fang, Z.; Li, Q.; Liu, T.T.; et al. Tryptanthrin suppresses multiple inflammasome activation to regulate NASH progression by targeting ASC protein. *Phytomedicine* **2024**, *131*, doi:10.1016/j.phymed.2024.155758.
27. Fan, Z.W.; Jia, M.X.; Zhou, J.; Zhu, Z.L.; Wu, Y.M.; Lin, X.W.; Qian, Y.M.; Lian, J.S.; Hua, X.; Dong, J.H.; et al. Pharmacological targeting cGAS/STING/NF-κB axis by tryptanthrin induces microglia polarization toward M2 phenotype and promotes functional recovery in a mouse model of spinal cord injury. *Neural Regen. Res.* **2025**, *20*, 3287-3301, doi:10.4103/nrr.Nrr-d-23-01256.
28. Hesse-Macabata, J.; Morgner, B.; Elsner, P.; Hipler, U.C.; Wiegand, C. Tryptanthrin promotes keratinocyte and fibroblast responses *in vitro* after infection with *Trichophyton benhamiae* DSM6916. *Sci. Rep.* **2020**, *10*, doi:10.1038/s41598-020-58773-2.
29. Han, N.R.; Moon, P.D.; Kim, H.M.; Jeong, H.J. Tryptanthrin ameliorates atopic dermatitis through down-regulation of TSLP. *Arch. Biochem. Biophys.* **2014**, *542*, 14-20, doi:10.1016/j.abb.2013.11.010.
30. Han, N.R.; Kim, H.M.; Jeong, H.J. Tryptanthrin reduces mast cell proliferation promoted by TSLP through modulation of MDM2 and p53. *Biomed. Pharmacother.* **2016**, *79*, 71-77, doi:10.1016/j.biopha.2016.01.046.
31. Mexia, N.; Gaitanis, G.; Velegrald, A.; Soshilov, A.; Denison, M.S.; Magiatis, P. Pityriazepin and other potent AhR ligands isolated from *Malassezia furfur* yeast. *Arch. Biochem. Biophys.* **2015**, *571*, 16-20, doi:10.1016/j.abb.2015.02.023.
32. Cheng, H.M.; Wu, Y.C.; Wang, Q.M.; Song, M.; Wu, J.; Chen, D.; Li, K.; Wadman, E.; Kao, S.T.; Li, T.C.; et al. Clinical efficacy and IL-17 targeting mechanism of Indigo naturalis as a topical agent in moderate psoriasis. *BMC Complement. Altern. Med.* **2017**, *17*, doi:10.1186/s12906-017-1947-1.
33. Johansen, T.H.; Drejer, J.; Watjen, F.; Nielsen, E.O. A novel non-NMDA receptor antagonist shows selective displacement of low-affinity [^{3}H]kainate binding. *Eur. J. Pharmacol.* **1993**, *246*, 195-204, doi:10.1016/0922-4106(93)90031-4.
34. Verdoorn, T.A.; Johansen, T.H.; Drejer, J.; Nielsen, E.O. Selective block of recombinant glur6 receptors by NS-102, a novel non-NMDA receptor antagonist. *Eur. J. Pharmacol.* **1994**, *269*, 43-49, doi:10.1016/0922-4106(94)90024-8.
35. Tian, H.; Zhang, Q.G.; Zhu, G.X.; Pei, D.S.; Guan, Q.H.; Zhang, G.Y. Activation of c-Jun NH2-terminal kinase 3 is mediated by the GluR6•PSD-95•MLK3 signaling module following cerebral ischemia in rat hippocampus. *Brain Res.* **2005**, *1061*, 57-66, doi:10.1016/j.brainres.2005.09.001.
36. Carulla, P.; Bribián, A.; Rangel, A.; Gavín, R.; Ferrer, I.; Caelles, C.; del Río, J.A.; Llorens, F. Neuroprotective role of PrPC against kainate-induced epileptic seizures and cell death depends on the modulation of JNK3 activation by GluR6/7-PSD-95 binding. *Mol. Biol. Cell.* **2011**, *22*, 3041-3054, doi:10.1091/mbc.E11-04-0321.
37. Terryn, R.J., III; German, H.W.; Kummerer, T.M.; Sinden, R.R.; Baum, J.C.; Novak, M.J. Novel computational study on π-stacking to understand mechanistic interactions of tryptanthrin analogues with DNA. *Toxicol. Mech. Methods* **2014**, *24*, 73-79, doi:10.3109/15376516.2013.859194.

38. Zhao, L.F.; Liu, Y.C.; Qin, Q.P.; Ya, W.Z.; Duan, H.C. Tryptanthrin sulfonate: Crystal structure, cytotoxicity and DNA binding studies. In Proceedings of the 2nd International Conference on Chemical Engineering and Advanced Materials (CEAM 2012), Guangzhou, China, Jul 13-15, 2012, 2012; pp. 1694-1699.
39. Chen, G.S.; Bhagwat, B.V.; Liao, P.Y.; Chen, H.T.; Lin, S.B.; Chern, J.W. Specific stabilization of DNA triple helices by indolo[2,1-b]quinazolin-6,12-dione derivatives. *Bioorg. Med. Chem. Lett.* **2007**, *17*, 1769-1772, doi:10.1016/j.bmcl.2006.12.079.
40. Jun, K.Y.; Park, S.E.; Liang, J.L.; Jahng, Y.; Kwon, Y. Benzo[b]tryptanthrin inhibits MDR1, topoisomerase activity, and reverses adriamycin resistance in breast cancer cells. *ChemMedChem* **2015**, *10*, 827-835, doi:10.1002/cmdc.201500068.
41. Yu, S.T.; Chern, J.W.; Chen, T.M.; Chiu, Y.F.; Chen, H.T.; Chen, Y.H. Cytotoxicity and reversal of multidrug resistance by tryptanthrin-derived indoloquinazolines. *Acta Pharmacol. Sin.* **2010**, *31*, 259-264, doi:10.1038/aps.2009.198.
42. Popov, A.; Klimovich, A.; Styshova, O.; Moskovkina, T.; Shchekotikhin, A.; Grammatikova, N.; Dezhenkova, L.; Kaluzhny, D.; Deriabin, P.; Gerasimenko, A.; et al. Design, synthesis and biomedical evaluation of mostotrin, a new water soluble tryptanthrin derivative. *Int. J. Mol. Med.* **2020**, *46*, 1335-1346, doi:10.3892/ijmm.2020.4693.
43. Zhang, Y.X.; Xiao, B.Y.; Yuan, S.Y.; Ding, L.L.; Pan, Y.F.; Jiang, Y.Y.; Sun, S.H.; Ke, X.S.; Cai, L.L.; Jia, L.J. Tryptanthrin targets GSTP1 to induce senescence and increases the susceptibility to apoptosis by senolytics in liver cancer cells. *Redox Biol.* **2024**, *76*, doi:10.1016/j.redox.2024.103323.
44. Yang, C.Y.; He, B.; Zheng, Q.; Wang, D.K.; Qin, M.M.; Zhang, H.; Dai, W.B.; Zhang, Q.; Meng, X.B.; Wang, X.Q. Nano-encapsulated tryptanthrin derivative for combined anticancer therapy via inhibiting indoleamine 2,3-dioxygenase and inducing immunogenic cell death. *Nanomedicine* **2019**, *14*, 2423-2440, doi:10.2217/nnm-2019-0074.
45. Chang, C.S.; Bai, L.Y.; Chiu, C.F.; Hu, J.L.; Weng, J.R. Discovery of the tryptanthrin-derived indoloquinazoline as an anti-breast cancer agent via ERK/JNK activation. *Environ. Toxicol.* **2024**, doi:10.1002/tox.24226.
46. Popov, A.M.; Degenkova, L.G.; Moskovkina, T.V.; Grammatikova, N.E.; Kuzmich, A.S.; Chernikov, O.V.; Schekotichin, A.E.; Stonik, V.A. Biological activity and probable mechanisms of action of derivatives of tryptanthrin and mostotrin alkaloids. *Dokl. Biochem. Biophys.* **2022**, *507*, 363-366, doi:10.1134/s1607672922340105.
47. Yang, H.R.; Li, Y.; Shi, W.B.; Zhang, W.W. Characterization of tryptanthrin as an antibacterial reagent inhibiting Vibrio splendidus. *Appl. Microbiol. Biotechnol.* **2024**, *108*, doi:10.1007/s00253-024-13158-7.
48. Narendrakumar, L.; Theresa, M.; Chandrika, S.K.; Thomas, S. Tryptanthrin, a potential biofilm inhibitor against toxigenic *Vibrio cholerae*, modulating the global quorum sensing regulator, LuxO. *Biofouling* **2019**, *35*, 1093-1103, doi:10.1080/08927014.2019.1696315.
49. Iwata, T.; Watanabe-Yanai, A.; Tamamura-Andoh, Y.; Arai, N.; Akiba, M.; Kusumoto, M. Tryptanthrin reduces *Campylobacter jejuni* colonization in the chicken gut by a bactericidal mechanism. *Appl. Environ. Microbiol.* **2023**, *89*, doi:10.1128/aem.01701-22.
50. Lin, C.J.; Chang, Y.L.; Yang, Y.L.; Chen, Y.L. Natural alkaloid tryptanthrin exhibits novel anticryptococcal activity. *Med. Mycol.* **2021**, *59*, 545-556, doi:10.1093/mmy/myaa074.
51. Ren, W.; Zhao, Q.; Yu, M.; Guo, L.; Chang, H.M.; Jiang, X.; Luo, Y.F.; Huang, W.; He, G. Design and synthesis of novel spirooxindole-indenoquinoxaline derivatives as novel tryptophanyl-tRNA synthetase inhibitors. *Mol. Divers.* **2020**, *24*, 1043-1063, doi:10.1007/s11030-019-10011-2.
52. Amara, R.; Awad, H.; Chaker, D.; Bentabed-Ababsa, G.; Lassagne, F.; Erb, W.; Chevallier, F.; Roisnel, T.; Dorcet, V.; Fajloun, Z.; et al. Conversion of isatins to tryptanthrins, heterocycles

endowed with a myriad of bioactivities. *Eur. J. Org. Chem.* **2019**, *2019*, 5302-5312, doi:10.1002/ejoc.201900352.

53. Frolova, S.G.; Klimina, K.M.; Kumar, R.; Vatlin, A.A.; Salunke, D.B.; Kendrekar, P.; Danilenko, V.N.; Maslov, D.A. Identification of mutations conferring tryptanthrin resistance to *Mycobacterium smegmatis*. *Antibiotics* **2021**, *10*, doi:10.3390/antibiotics10010006.
54. Kutlu, Z.; Halici, Z.; Gedikli, S.; Diyarbakir, B.; Civelek, M.S. A lead target molecule for excisional wound healing: tryptanthrin compound. *Iran. J. Pharm. Res.* **2022**, *21*, doi:10.5812/ijpr-127665.
